# The heat shock response, determined by QuantiGene multiplex, is impaired in HD mouse models and not caused by HSF1 reduction

**DOI:** 10.1038/s41598-021-88715-5

**Published:** 2021-04-27

**Authors:** Casandra Gomez-Paredes, Michael A. Mason, Bridget A. Taxy, Aikaterini S. Papadopoulou, Paolo Paganetti, Gillian P. Bates

**Affiliations:** 1 Huntington’s Disease Centre, Department of Neurodegenerative Disease and UK Dementia Research Institute at UCL, Queen Square Institute of Neurology, University College London, London, WC1N 3BG UK; 2 Laboratory for Biomedical Neurosciences, Neurocenter of Southern Switzerland, Ente Ospedaliero Cantonale and Faculty of Biomedical Sciences, Università Della Svizzera Italiana, Lugano, Switzerland

**Keywords:** Molecular biology, Protein folding, Chaperones, Diseases, Huntington's disease

## Abstract

Huntington’s disease (HD) is a devastating neurodegenerative disorder, caused by a CAG/polyglutamine repeat expansion, that results in the aggregation of the huntingtin protein, culminating in the deposition of inclusion bodies in HD patient brains. We have previously shown that the heat shock response becomes impaired with disease progression in mouse models of HD. The disruption of this inducible arm of the proteostasis network is likely to exacerbate the pathogenesis of this protein-folding disease. To allow a rapid and more comprehensive analysis of the heat shock response, we have developed, and validated, a 16-plex QuantiGene assay that allows the expression of *Hsf1* and nine heat shock genes, to be measured directly, and simultaneously, from mouse tissue. We used this QuantiGene assay to show that, following pharmacological activation in vivo, the heat shock response impairment in tibialis anterior, brain hemispheres and striatum was comparable between zQ175 and R6/2 mice. In contrast, although a heat shock impairment could be detected in R6/2 cortex, this was not apparent in the cortex from zQ175 mice. Whilst the mechanism underlying this impairment remains unknown, our data indicated that it is not caused by a reduction in HSF1 levels, as had been reported.

## Introduction

Huntington’s disease (HD) is a progressive neurodegenerative disorder, of autosomal dominant inheritance, caused by the expansion of a CAG repeat, within exon 1 of the huntingtin (*HTT*) gene^1,2^. This encodes an abnormally long polyglutamine tract that leads to the formation of insoluble aggregates in brains and other tissues from HD patients as well as in mouse models of the disease^3–8^. Abnormal intracellular interactions of these aggregates with other proteins destabilise the integrity of the proteome, affecting essential cellular pathways related to transcriptional regulation, mitochondrial function, DNA repair, neuronal circuitry and protein homeostasis, among others, and resulting in neuronal dysfunction and death^9–11^.

The heat shock response is a protective cellular mechanism that forms one arm of the stress-induced protein quality control system. It assists in maintaining the functionality and stability of the proteome during protein synthesis, protein folding as well as by removing aberrantly-folded proteins^12^. It is induced under circumstances of environmental stress leading to proteotoxicity. The master regulator of the heat shock response is heat shock factor 1 (HSF1) that tightly regulates transcriptional activity of the heat shock genes, encoding heat shock proteins or chaperones, which are the main effectors of the response^13^. The activation, inhibition and degradation of HSF1 are regulated by post-translational modifications and interactions with other chaperones. HSF1 becomes activated in response to stress, is released from its HSP90 inhibitory complex, translocates to the nucleus, trimerizes, becomes hyperphosphorylated and binds to the promoters of the heat shock genes^14–16^.

The manipulation of heat shock proteins has been reported to provide beneficial effects in the suppression of huntingtin toxicity^17,18^. The heat shock response, that induces the coordinated expression of multiple heat shock genes, is an attractive therapeutic target, and the activation of HSF1 has been the subject of numerous studies using HD models^19–24^. We previously reported that HSF1 activation and heat shock gene induction, by the pulsed treatment with the HSP90 inhibitor NVP-HSP990^25^, ameliorated some motor phenotypes and reduced the aggregate load in brain and muscle of R6/2 mice. However, these effects were transient, because the ability to mount a heat shock response became impaired with disease progression^26^. This impairment was demonstrated in R6/2 mice^27^ that are transgenic for a genomic fragment encoding the exon 1 HTT protein and the *Hdh*Q150 knockin model^28^ in which an expanded CAG repeat has been inserted into the endogenous mouse gene. It was not related to the levels or activation of HSF1, but there was a decreased HSF1 occupancy at heat shock gene promoters which correlated with the hypoacetylation of histone H4^26^.

However, a recent study reported that the levels of HSF1 are decreased in the zQ175 knockin mouse model of HD^29,30^, in which mouse exon 1 has been replaced with a mutant version of human exon 1, as well as in HD cell models and HD patient cells and tissues; and that this correlated with lower chaperone levels^31^. This prompted us to conduct an analysis of the heat shock response in zQ175 mice. We developed a QuantiGene multiplex assay to measure the induction of multiple heat shock genes directly from tissue lysates. We employed the brain-penetrant HSP90 inhibitor, NVP-HSP990, to induce a heat shock response in the zQ175 mice and their wild-type counterparts, and used the QuantiGene assay, to measure heat shock gene expression in brain hemispheres, cortex, striatum and tibialis anterior and compared this to the impairment detected in R6/2 mice. We conducted a comprehensive characterisation of HSF1 antibodies to enable us to quantify the levels of basal and activated HSF1 with disease progression. We confirmed that zQ175 mice develop an age-dependent defect in the heat shock response in brain and muscle, but our data provided no evidence to indicate that this impairment was caused by a reduction in the levels of HSF1.

## Results

### Basal and activated levels of HSF1 do not change in zQ175 mice with disease progression

In order to investigate whether the basal or activated levels of HSF1 were altered in zQ175 mice with disease progression, we first conducted a comprehensive analysis of HSF1 antibodies. To ensure that we were detecting the HSF1 protein on western blots, we included HSF1 knockout lysates as a negative control and lysates from wild-type mice that had been treated with the HSP90 inhibitor, NVP-HSP990, as a positive control. NVP-HSP990 is a potent brain-penetrant HSP90 inhibitor that induces the heat shock response, resulting in the hyperphosphorylation of HSF1 which can be observed as a hypershift on western blots. All HSF1 antibodies (Supplementary Table S1 online) were tested using lysates that had been prepared in four different lysis buffers, with two alternative blocking solutions, at a series of dilutions, and the most promising were further tested by comparing in-house and precast gels. In most cases, these antibodies did not detect HSF1, and in all cases they recognised alternative proteins (Supplementary Fig. S1 online). Only two of the 12 antibodies tested, Proteintech 51034-1-AP and Bethyl A303-176A, detected a band that was absent in the HSF1 knockout lysates and that was hypershifted in the NVP-HSP990 treated samples.

**Figure 1:**
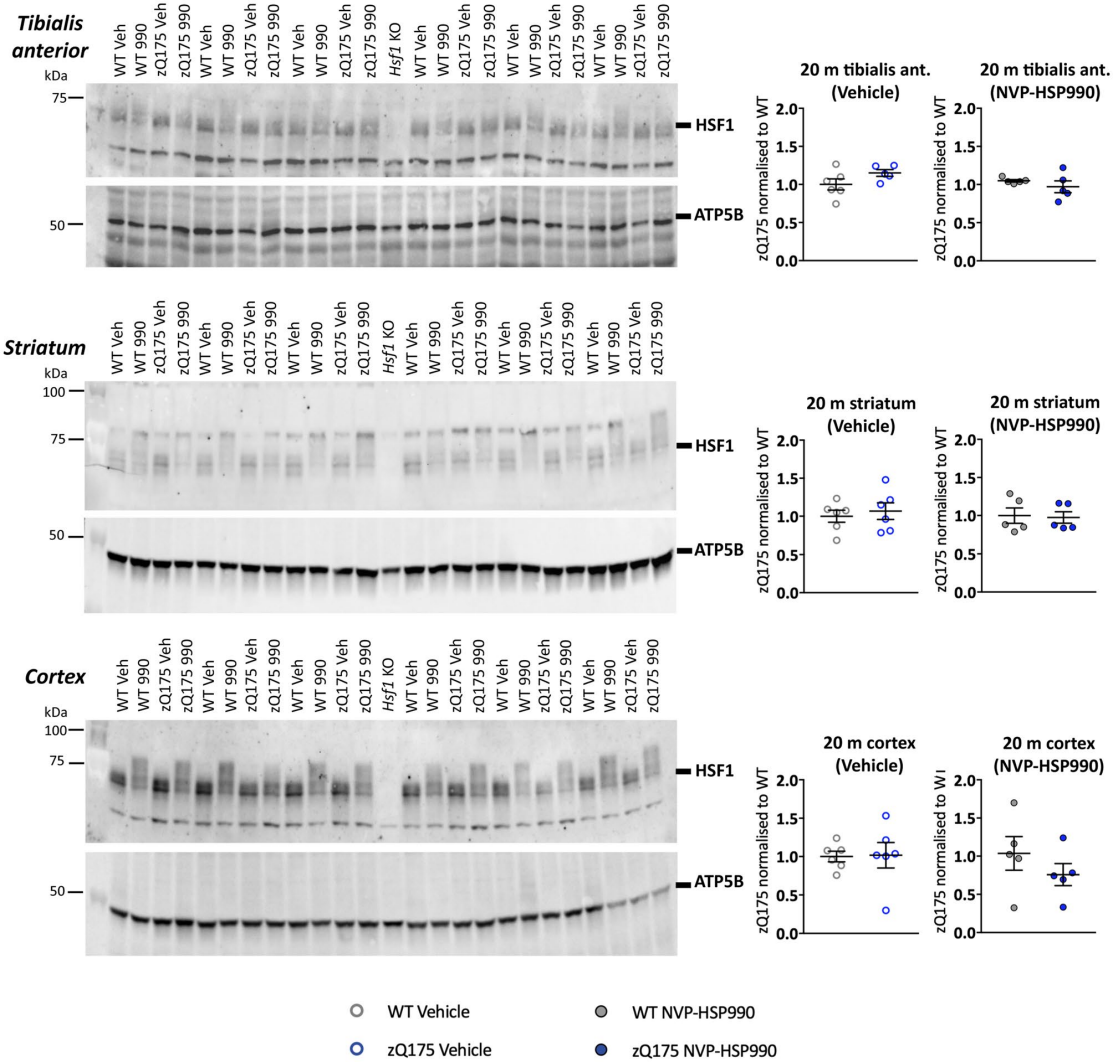
Neither basal nor activated levels of HSF1 change with disease progression in zQ175 tibialis anterior, striatum or cortex. On the left-hand side, western blot images showing HSF1 protein (top) or ATP5B (bottom) levels in tibialis anterior, striatum and cortex of zQ175 and wild-type mice treated with NVP-HSP990 or vehicle (*n* = 5–6/genotype/treatment) at 20 months of age. HSF1 antibody 51034-1-AP (Proteintech) was used in cortex and tibialis anterior and antibody A303-176A (Bethyl Laboratories) in striatum. Quantification was performed separately for the vehicle and NVP-HSP990 treated samples and normalised to wild-type in each case (right-hand side). There were no statistically significant differences between the genotypes for either treatment. Statistical analysis was by unpaired Student’s *t*-test. Mean ± SEM. The test statistics can be found in Supplementary Table S6 online. Uncropped blots are presented in Supplementary Figs. S2–S4 online. *Veh* vehicle, *990* NVP-HSP990, *WT* wild-type, *m* months, *KO* knockout, *ant.* anterior.

Wild-type and zQ175 animals were studied at 3, 12 and 20 months of age, spanning the entire disease course to end-stage disease. Animals were dosed with 12 mg/kg NVP-HSP990 or vehicle (2% methylcellulose in 0.9% saline solution) (*n* = 6 mice/genotype/treatment/age) and cortex, striatum and tibialis anterior were harvested 4 h post-dosing. At this time point, the hyperphosphorylation of HSF1 can be observed as a hypershift on western blots^26^. To determine whether HSF1 was altered with disease progression in zQ175 mice, we used the tissues from the 20-month cohort for protein analysis. Tissue samples were lysed using optimised experimental conditions and subjected to protein analysis by western blot (Fig. 1). There was no difference in the basal levels of HSF1 (vehicle) or in the activated levels, as indicated by the hypershift after NVP-HSP990 treatment, between wild-type and zQ175 mice at 20 months of age. Therefore, consistent with our previous analysis of the R6/2 and *Hdh*Q150 models^26^, we found no evidence to support the proposal that the heat shock impairment that occurs with disease progression in mouse models of HD was caused by a reduction in HSF1.

### Development and validation of a QuantiGene multiplex assay for measuring the expression of heat shock genes

Investigations of the heat shock response generally focus on the expression of *Hspa1a/b* (HSP70) and one or two other heat shock genes using real-time quantitative PCR (qPCR). The QuantiGene multiplex system utilises branched DNA technology to amplify signals from captured genes of interest^32,33^ and allows the expression of multiple genes to be determined simultaneously, directly from the tissue lysate. Before designing a QuantiGene multiplex assay to measure the expression of all heat shock genes, we validated the method with a small plex that allowed a direct comparison of heat shock gene expression with qPCR. This initial multiplex assay (8-plex) contained probes to detect three major heat shock genes: *Hspa1a/b* (HSP70) *Hspb1* (HSP25) and *Dnajb1* (HSP40), the master regulator of the heat shock response *Hsf1* and several housekeeping genes (*Atp5b*, *Rpl13a*, *Canx* and *Eif4a2*)^34^. We administered NVP-HSP990 (12 mg/kg) or vehicle to wild-type mice at nine weeks of age and collected brain hemispheres and tibialis anterior muscles at 4 h after dosing. One hemisphere and one tibialis anterior were used for the QuantiGene multiplex assay, and the other hemisphere and tibialis anterior were used for qPCR.

**Figure 2:**
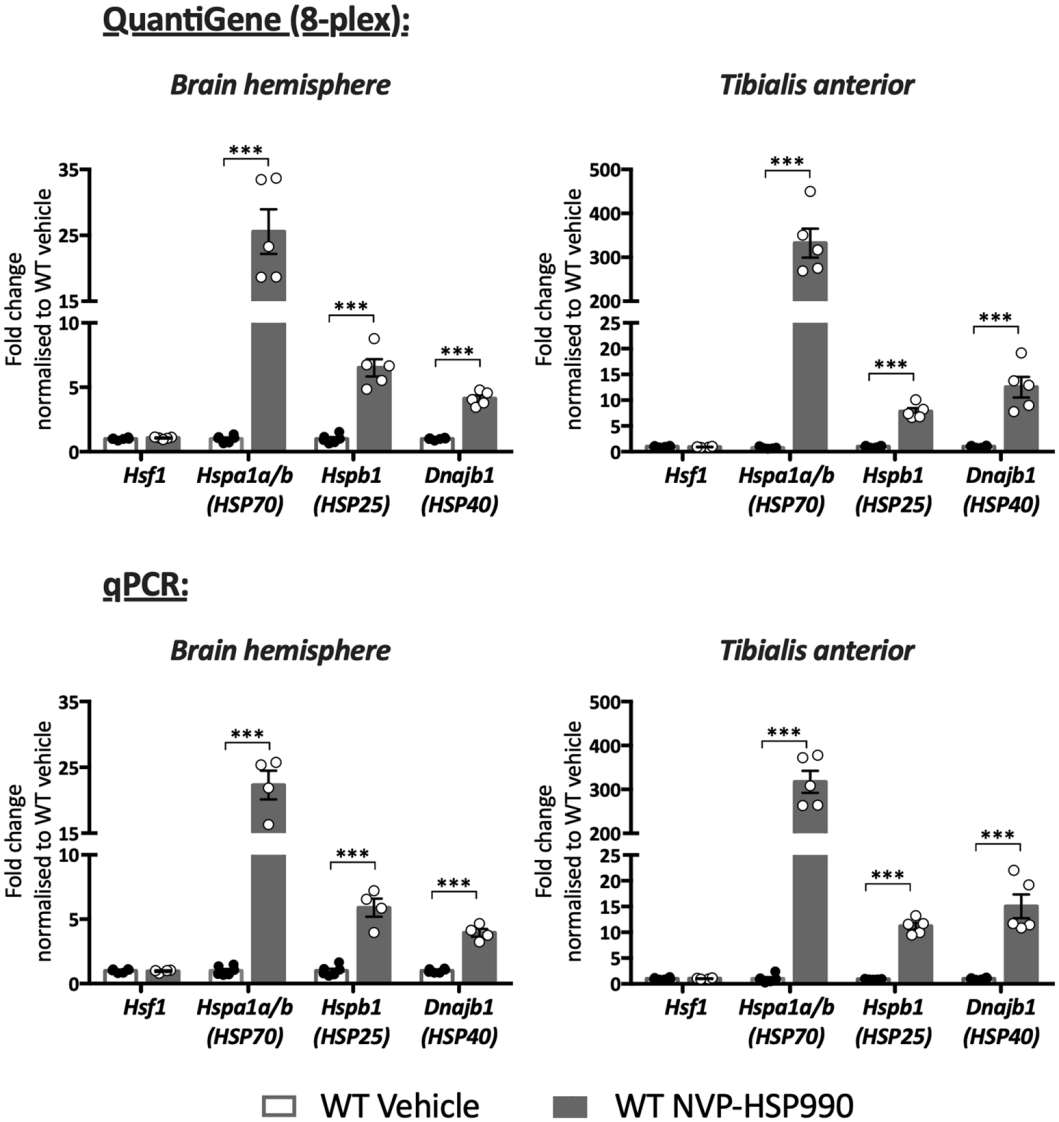
Validation of QuantiGene multiplex assay to measure the expression of heat shock genes in mouse brain hemispheres and tibialis anterior muscle. Increase in the expression levels of *Hsf1*, *Hspa1a/b*, *Hspb1* and *Dnajb1* in wild-type mice was determined by QuantiGene (8-plex) (top graphs) and qPCR (bottom graphs) after treatment with NVP-HSP990 as compared to vehicle. The induction of the heat shock response resulted in an increase in the expression level of *Hspa1a/b*, *Hspb1* and *Dnajb1*, but not in *Hsf1* as would be expected. There was no difference in the fold changes in gene expression as measured by QuantiGene and qPCR. *n* = 4–6/treatment. Statistical analysis was by unpaired Student’s *t*-test. Mean ± SEM. ***p ≤ 0.001. Test statistics can be found in Supplementary Table S7 online. *WT* wild-type.

The QuantiGene plex was optimised for sample input, to ensure that we were able to detect basal and induced levels of the heat shock genes, as well as to test the stability of the housekeeping genes, in both brain and muscle tissues. This was achieved by performing serial dilutions according to the manufacturer’s recommendations. Tissue dilutions were selected that allowed adequate median fluorescent intensities (MFI) with no signal saturation in any of the heat shock or housekeeping genes (Supplementary Figs. S5, S6, Supplementary Table S2 online). The brain hemispheres and tibialis anterior muscles were homogenised for QuantiGene analysis, RNA was extracted for qPCR and gene expression was determined. Highly comparable data were generated by both techniques, with similar results for the fold change in the expression of all genes in both brain and muscle (Fig. 2).

### Kinetics of pharmacological induction of the heat shock response by NVP-HSP990

Before using a QuantiGene plex to measure the heat shock response in zQ175 mice, we assessed the kinetics of the pharmacological activation of the heat shock response after dosing with NVP-HSP990. Wild-type mice at nine weeks of age received an acute dose of NVP-HSP990 (12 mg/kg) or vehicle and brain hemispheres were collected at 4, 8, 12, 16 or 20 h after dosing (*n* = 4/treatment/time point). Samples were processed for QuantiGene (8-plex) and the level of heat shock gene induction was determined (Supplementary Fig. S7 online)). The highest expression level occurred at 4 h post-dosing for *Hspa1a/b* and between 4 and 8 h for *Hspb1* and *Dnajb1*, progressively returning to baseline levels by 20 h. Therefore, although the kinetics of induction for these three genes did not proceed at the same rate, 4 h after dosing was an appropriate time point to use for the following RNA-based experiments.

### The effect of disease progression on the induction of heat shock genes in the tibialis anterior, striatum and cortex of zQ175 mice

Although the zQ175 mice had been used to determine whether a reduction in HSF1 might be responsible for an impairment in the heat shock response in HD^31^, whether such an impairment existed had not been investigated in this mouse model. Therefore, we set out to use QuantiGene to monitor the induction of the heat shock response in zQ175 brain and muscle tissues during the entire course of the disease. We used the tibialis anterior, striatum and cortex from wild-type and zQ175 mice at 3, 12 and 20 months of age that had been dosed with 12 mg/kg NVP-HSP990 or vehicle. Therefore, for the 20-month cohort, these tissues were from the same set of mice as had been used to generate the HSF1 western blot data in Fig. 1 (one cortical hemisphere, one striata and one tibialis anterior muscle were processed for protein work and the other ones for QuantiGene analysis).

In order to have a more comprehensive picture of the impairment of the heat shock response in zQ175 mice, we designed a QuantiGene multiplex assay (16-plex), containing a more extensive set of heat shock genes, selected on the basis of microarray gene expression and RNAseq data previously generated by our group^22,26^. The 16-plex included *Hsf1*, the heat shock genes *Hspa1a/b* (HSP70), *Dnaja1* (HSP40), *Dnajb1* (HSP40), *Hspb1* (HSP25), *Hspd1* (HSP60), *Hspe1* (HSP10), *Hsph1* (HSP110), *Hsp90aa1* (HSP90α) and *Hsp90ab1* (HSP90β), and the housekeeping genes *Atp5b*, *Eif4a2*, *Rpl13a*, *Canx*, *Gapdh* and *Sdha*. For the housekeeping genes, we performed a dilution series of zQ175 and wild-type tissues, taken at 3 and 20 months of age, to determine the optimal dilutions to allow adequate MFI with no signal saturation (Supplementary Figs. S8, S9, S10 online). For the heat shock genes, dilution of wild-type lysates was used to ensure that an adequate MFI could be detected with no signal saturation, and with signals that were over the lower limit of detection (Supplementary Figs. S11, S12, S13 online). The dilutions that were selected are presented in Supplementary Table S2 online.

**Figure 3:**
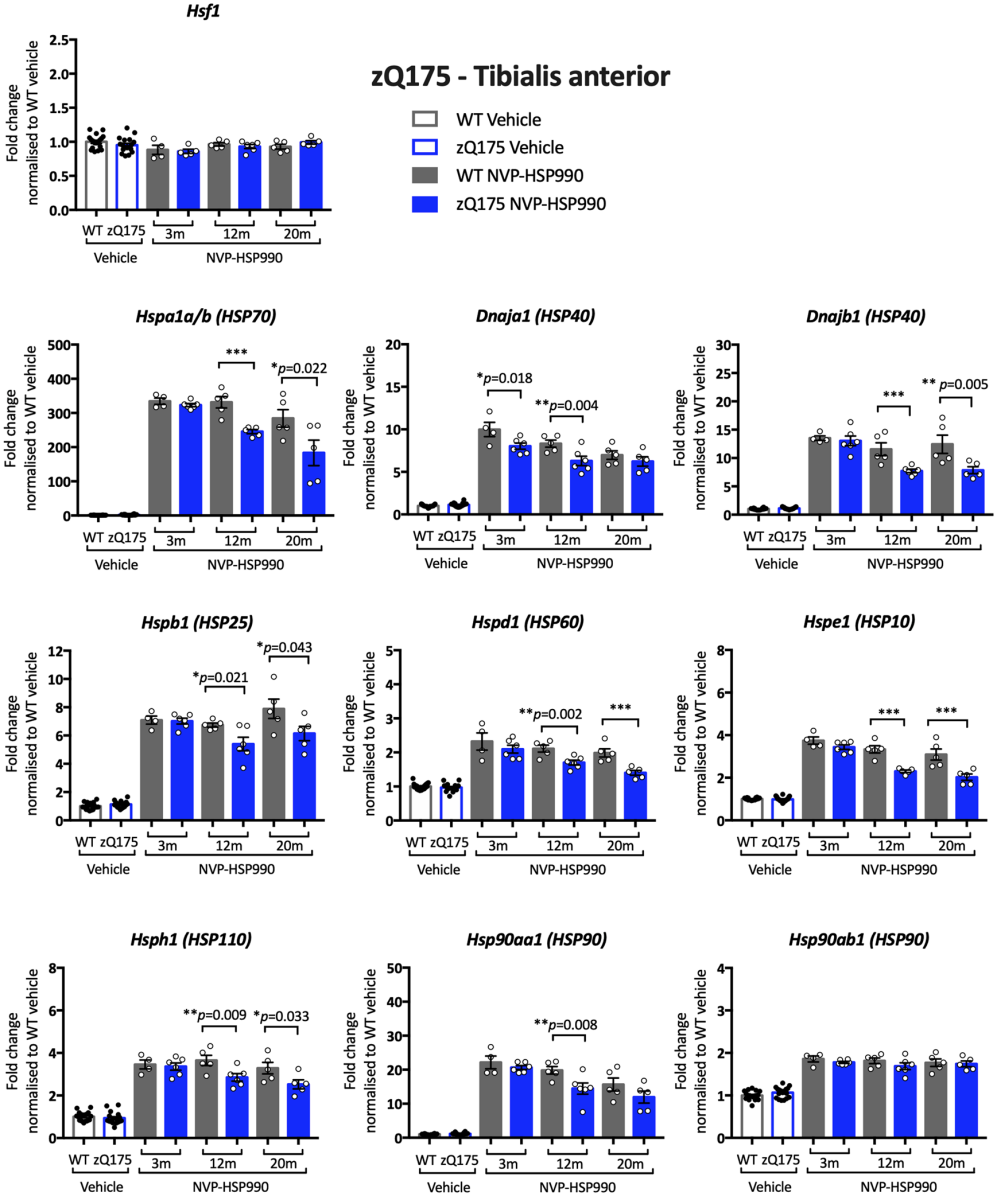
Analysis of the heat shock response in the tibialis anterior of zQ175 mice with disease progression. The expression of heat shock genes was measured using the QuantiGene 16-plex assay in the tibialis anterior of wild-type and zQ175 mice at 3, 12 and 20 months of age that had been treated with vehicle or NVP-HSP990. The NVP-HSP990 treated samples were normalised to the corresponding age-matched wild-type vehicle treated samples. For simplicity, the wild-type vehicle samples and the zQ175 vehicle samples from the three ages were combined. Five NVP-HSP990 treated samples were excluded from the analysis, because the tibialis anterior heat shock gene expression levels were comparable to those treated with vehicle. These were: at 3 months, two wild type; at 12 months, one wild-type; at 20 months, one wild-type and one zQ175. *N* = 4–6/genotype/treatment/age. Statistical analysis was by two-way ANOVA with Bonferroni correction for multiple comparisons. Mean ± SEM. ***p ≤ 0.001. Test statistical values can be found in Supplementary Table S9 online. *WT* wild-type, *m* months.

**Figure 4:**
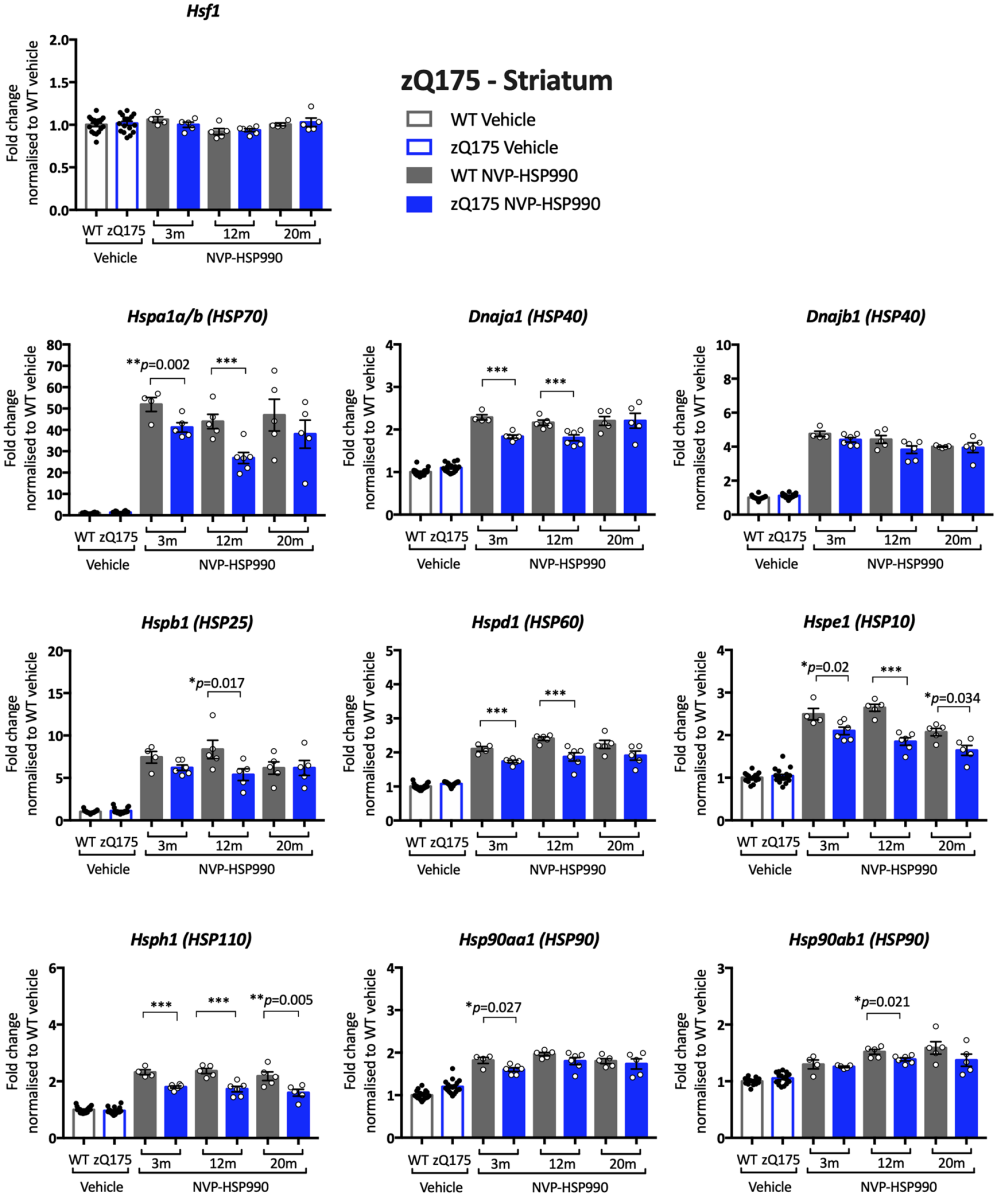
Analysis of the heat shock response in the striatum of zQ175 mice with disease progression. The expression of heat shock genes was measured using the QuantiGene 16-plex assay in the striatum of wild-type and zQ175 mice at 3, 12 and 20 months of age that had been treated with vehicle or NVP-HSP990. The NVP-HSP990 treated samples were normalised to the corresponding age-matched wild-type vehicle treated samples. For simplicity, the wild-type vehicle samples and the zQ175 vehicle samples from the three ages were combined. Five NVP-HSP990 treated samples were excluded from the analysis, because the tibialis anterior heat shock gene expression levels were comparable to those treated with vehicle. These were: at 3 months, two wild type; at 12 months, one wild type; at 20 months, one wild-type and one zQ175. *N* = 4–6/genotype/treatment/age. Statistical analysis was by two-way ANOVA with Bonferroni correction for multiple comparisons. Mean ± SEM. ***p ≤ 0.001. Test statistical values can be found in Supplementary Table S10 online. *WT* wild-type, *m* months.

The QuantiGene 16-plex was then applied to compare the expression of *Hsf1* and heat shock genes in the tibialis anterior, striatum and cortex of wild-type and zQ175 mice treated with NVP-HSP990 or vehicle at 3, 12 and 20 months of age. The levels of *Hsf1* expression remained unaltered after NVP-HSP990 treatment as compared to vehicle, in all wild-type and zQ175 tissues at all ages (Figs. 3, 4 and Supplementary Fig. S14 online).

The expression levels of heat shock genes, were generally much higher after heat shock induction in tibialis anterior, than in brain tissues (Figs. 3, 4 and Supplementary Fig. S14 online), as previously observed^22^. Comparison of the induction of the heat shock genes in tibialis anterior between zQ175 and wild-type mice, indicated that the heat shock response was impaired. The induction of *Hspa1a/b*, *Dnaja1*, *Dnajb1*, *Hspb1*, *Hspd1*, *Hspe1*, *Hsph1* and *Hsp90aa1* was lower in zQ175 tibialis as compared to wild type by 12 months of age in all cases (Fig. 3). The fold decrease in induction, as illustrated in Fig. 3, may not reflect the extent of the impairment, because differences in the kinetics of induction, between heat shock genes, means that the peak of induction will not have been reached in all cases by four hours post-dosing.

A reduction in heat shock gene induction was observed in the striatum for *Hspa1a/b*, *Dnaja1*, *Hspd1*, *Hspe1* and *Hsph1*, which, in all cases, could be detected at 3 months of age (Fig. 4). The earlier appearance of the impairment in striatum as compared to tibialis anterior may reflect the earlier onset of pathology in brain than muscle in both R6/2 and knock-in mouse models of HD^35^. However, we did not detect any consistent evidence for a decreased induction in the levels of *Dnajb1* or *Hspb1* (Supplementary Table S10) the induction of which had been found to be impaired in the brains of both R6/2 and *Hdh*Q150 mice^26^. In contrast to tibialis anterior and striatum, there was little evidence of a heat shock response impairment in the zQ175 cortex. A decrease in the induction of *Hspa1a/b*, *Hspe1* and *Hsph1* was observed but only at 12 months of age (Supplementary Fig. S14 online).

### Comparison of the heat shock response impairment between R6/2 and zQ175 mice

**Figure 5:**
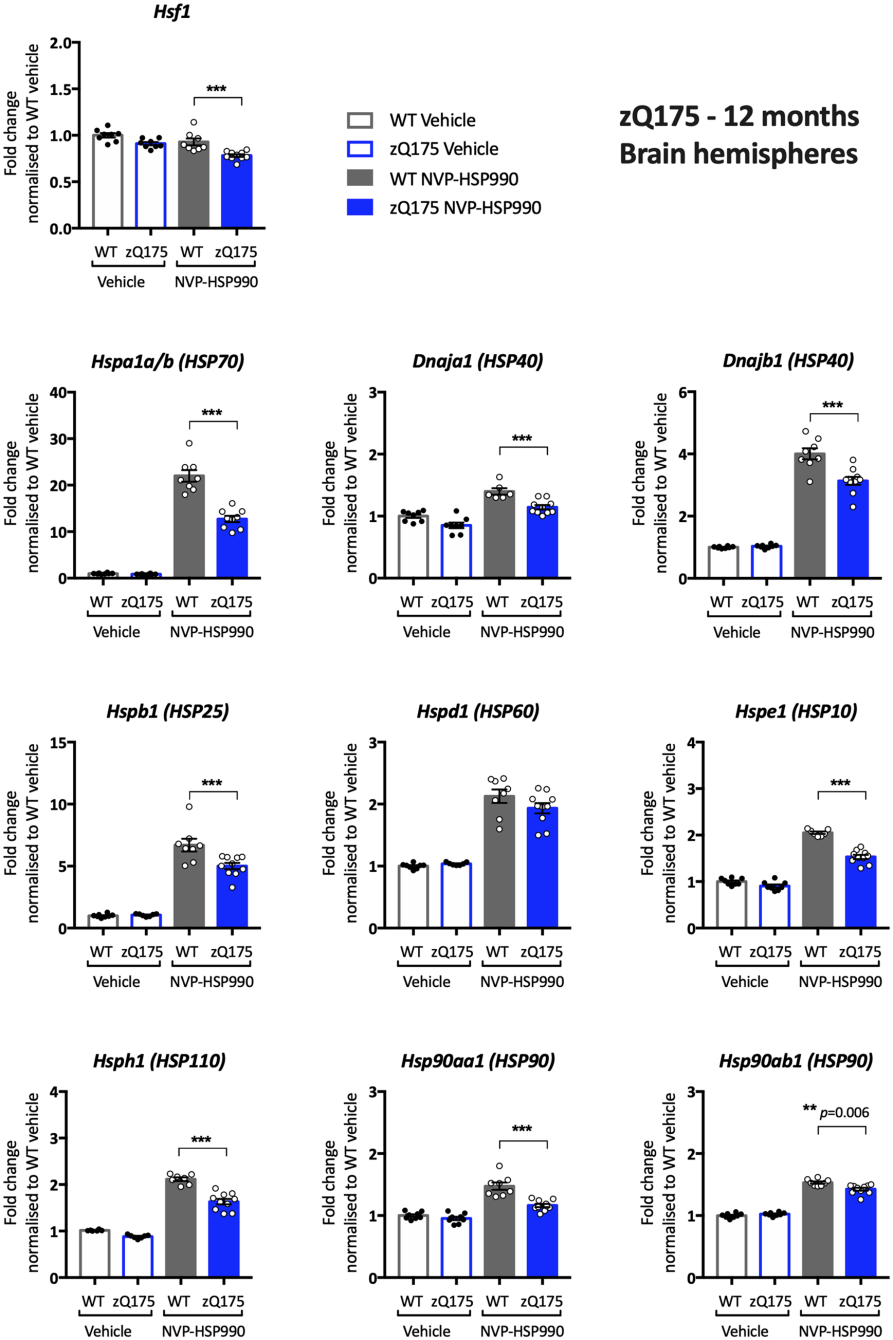
Analysis of the heat shock response in the brain hemispheres of zQ175 mice at 12 months of age. The expression of heat shock genes was measured using the QuantiGene 16-plex assay in the brain hemispheres of wild-type and zQ175 mice at 12 months of age that had been treated with vehicle or NVP-HSP990. The NVP-HSP990 treated samples were normalised to the corresponding wild-type vehicle treated samples. *N* = 6–10/genotype/treatment. Statistical analysis was by two-way ANOVA with Bonferroni correction for multiple comparisons. Mean ± SEM. ***p ≤ 0.001. Test statistical values can be found in Supplementary Table S12 online. *WT* wild-type.

**Figure 6:**
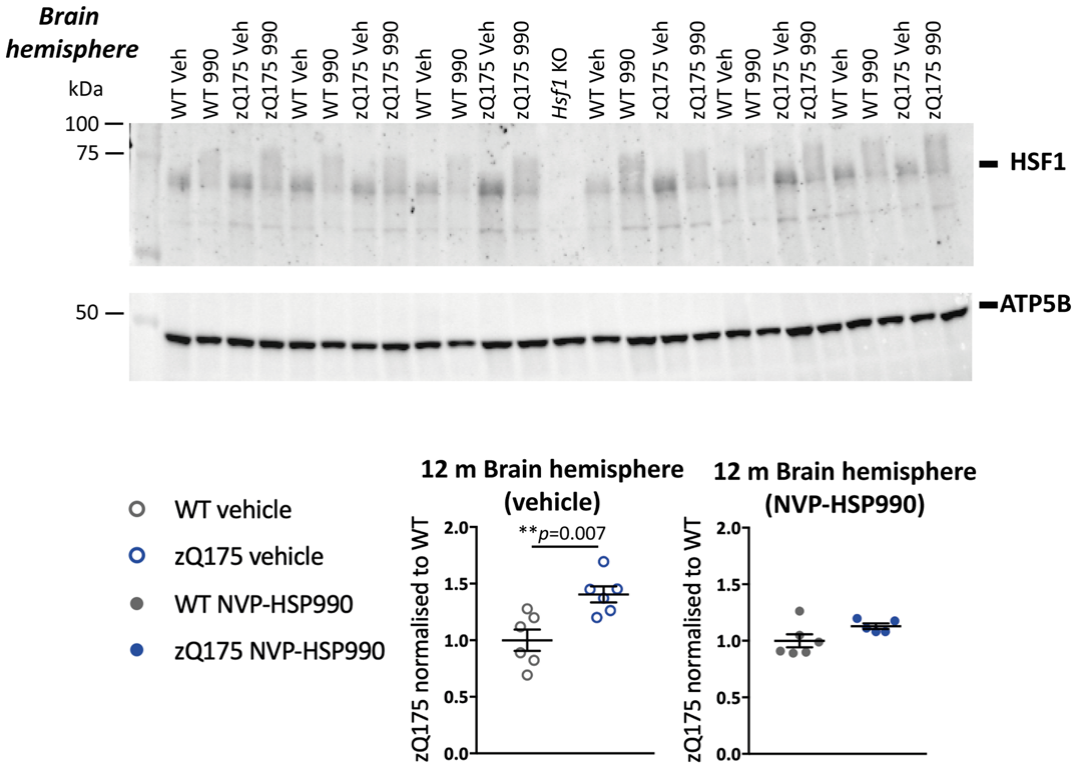
Neither basal nor activated levels of HSF1 are decreased with disease progression in zQ175 brain hemispheres. Western blot images showing HSF1 protein (top) or ATP5B (bottom) levels in brain hemispheres from zQ175 and wild-type mice treated with NVP-HSP990 or vehicle (*n* = 5–6/genotype/treatment) at 12 months of age probed with HSF1 antibody 51034-1-AP (Proteintech). Quantification was performed separately for the vehicle and NVP-HSP990 treated samples and normalised to wild-type in each case. HSF1 levels were higher in zQ175 brains than in wild type under basal conditions. There was no difference in HSF1 levels after induction with NVP-HSP990 between the genotypes. Statistical analysis was by unpaired Student’s *t* test. Mean ± SEM. The test statistics can be found in Supplementary Table S13 online. Uncropped blots are presented in Supplementary Fig. S17 online. *Veh* vehicle, *990* NVP-HSP990, *WT* wild-type, *m* months, *KO* knockout, *ant.* anterior.

Our previous analysis of the heat shock response in R6/2 and *Hdh*Q150 knockin mice had used qPCR to measure the induction of *Hspa1a/b*, *Dnajb1* and *Hspb1* in brain hemispheres. Therefore, to determine whether there was a difference in the impairment of the heat shock response in the brains of zQ175 mice, as compared to R6/2 and *Hdh*Q150, we used the QuantiGene plex to measure the induction of heat shock genes in one brain hemisphere from zQ175 and wild type mice at 12 months of age that had been treated with 12 mg/kg NVP-HSP990 or vehicle. Lysate dilutions were performed to optimise the assay conditions for the housekeeping genes (Supplementary Fig. S15 online) and heat shock genes (Supplementary Fig. S16 online). As with R6/2 and *Hdh*Q150, we found a decrease in the induction of *Hspa1a/b*, *Dnajb1* and *Hspb1* in zQ175 as compared to wild-type mice (Fig. 5). The induction of *Dnaja1*, *Hspe1*, *Hsph1, Hsp90aa1* and *Hsp90ab1* was also lower in zQ175 brains (Fig. 5). To determine whether a decrease in HSF1 protein could be responsible for this impairment the second hemisphere was used for western blotting. We found that the basal level of HSF1 was higher in zQ175 brains than in wild type, and that there was no difference in the level of activated HSF1 at 4 h post-induction between the two genotypes (Fig. 6).

**Figure 7:**
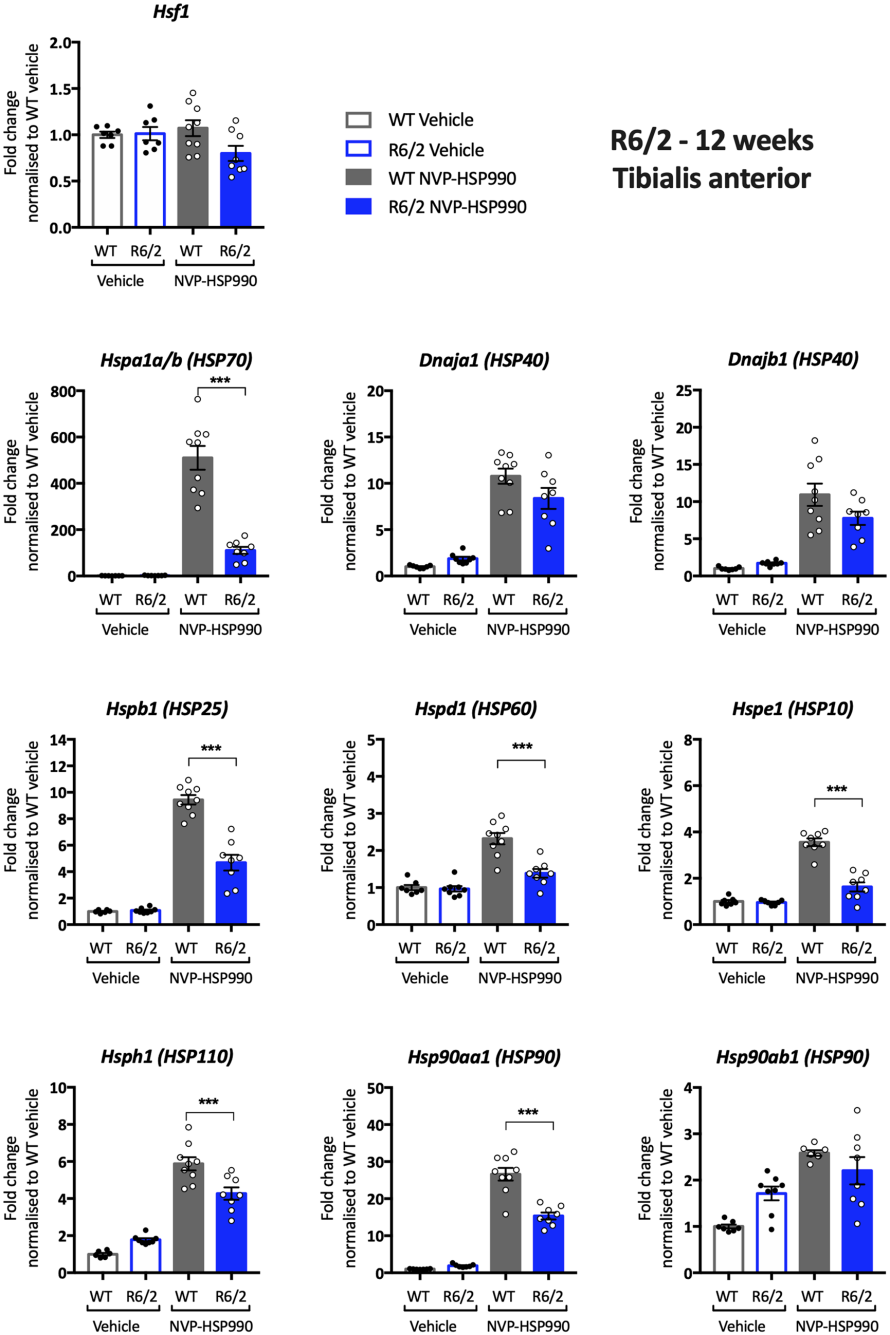
Analysis of the heat shock response in the tibialis anterior of R6/2 mice at 12 weeks of age. The expression of heat shock genes was measured using the QuantiGene 16-plex assay in the tibialis anterior of wild-type and R6/2 mice at 11–12 weeks of age that had been treated with vehicle or NVP-HSP990. The NVP-HSP990 treated samples were normalised to the corresponding wild-type vehicle treated samples. *N* = 6–9/genotype/treatment. Statistical analysis was by two-way ANOVA with Bonferroni correction for multiple comparisons. Mean ± SEM. ***p ≤ 0.001. Test statistical values can be found in Supplementary Table S14 online. *WT* wild-type.

**Figure 8:**
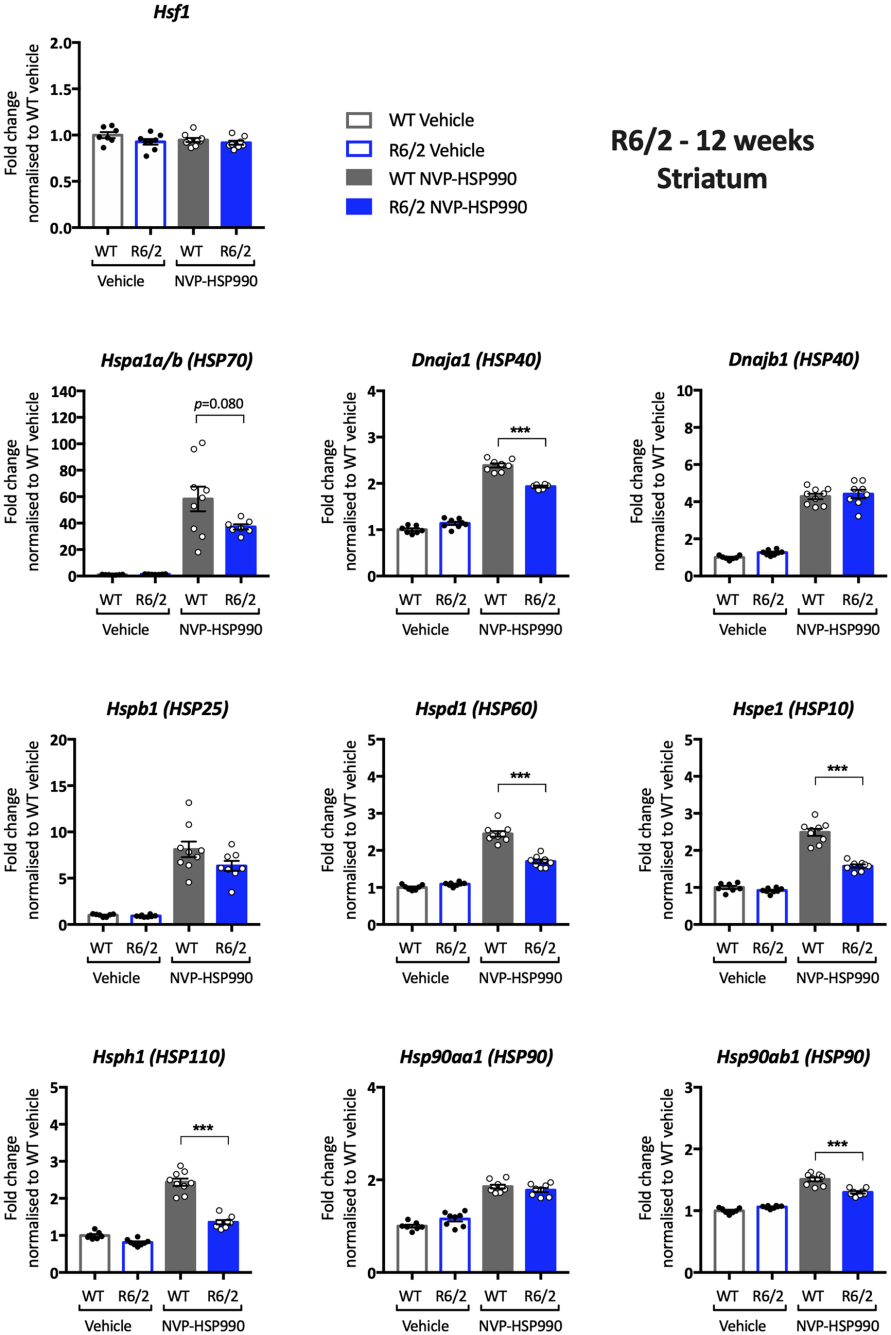
Analysis of the heat shock response in the striatum of R6/2 mice at 12 weeks of age. The expression of heat shock genes was measured using the QuantiGene 16-plex assay in the striatum of wild-type and R6/2 mice at 11–12 weeks of age that had been treated with vehicle or NVP-HSP990. The NVP-HSP990 treated samples were normalised to the corresponding wild-type vehicle treated samples. *N* = 6–9/genotype/treatment. Statistical analysis was by two-way ANOVA with Bonferroni correction for multiple comparisons. Mean ± SEM. ***p ≤ 0.001. Test statistical values can be found in Supplementary Table S15 online. *WT* wild-type.

Having established that there was no reason to doubt that the heat shock response was similarly impaired in brain hemispheres from zQ175, R6/2 and *Hdh*Q150 mice, we investigated the pattern of heat shock impairment in the tibialis anterior, striatum and cortex from R6/2 mice at 11–12 weeks of age. Lysate dilutions were performed to optimise the assay conditions for the housekeeping genes (Supplementary Figs. S18–20 online) and heat shock genes (Supplementary Figs. S21–S23 online). A robust impairment in the heat shock response was found in the tibialis for all heat shock genes except for *Dnaja1* and *Dnajb1* (Supplementary Table S14 online). The fold-change decrease in induction was generally greater for R6/2 (Fig. 7) than for zQ175 mice (Fig. 3), which may reflect differences in muscle pathology between the two models. The pattern of impairment in the striatum was remarkably similar for R6/2 (Fig. 8) and zQ175 mice (Fig. 4), in both cases, the genes showing an impaired induction (at more than one time point for zQ175) were: *Hspa1a/b*, *Dnaja1*, *Hspd1*, *Hspe1* and *Hsph1*. The only difference in the pattern of induction occurred in the cortex for whilst there was little evidence of a heat shock impairment in zQ175 mice (Supplementary Fig. S14 online), the induction of *Hspa1a/b* (p = 0.053), *Hspd1*, *Hspe1* and *Hdph1* was impaired in the R6/2 cortex (Supplementary Fig. S24 online).

## Discussion

Our discovery, that the heat shock response becomes impaired with disease progression in HD utilised the R6/2 transgenic and *Hdh*Q150 knockin mouse models. When investigating the basis of this impairment, we found no difference in the level of HSF1, or the activation of HSF1, between HD and wild-type mice at end-stage disease. However, there was less HSF1 bound to heat shock gene promoters and this correlated with the hypoacetylation of histone H4^26^. In contrast, an alternative study reported that HSF1 levels were decreased in the cortex, striatum and muscle of the zQ175 knockin mouse model of HD at 12 months of age^31^. As the heat shock response in zQ175 mice had not been described, and to better understand the discrepancy between these results, we have conducted a comprehensive analysis of the heat shock response in zQ175 mice at 3, 12 and 20 months of age, spanning the entire course of the disease. We found that the heat shock response was impaired in tibialis anterior, brain hemispheres and striatum, the pattern of which was comparable to that in R6/2 mice. There were no differences in the basal or induced levels of HSF1 in the brain or muscle between wild-type and zQ175 mice, and we found no evidence to indicate that the heat shock response impairment in HD is caused by a reduction in HSF1.

Heat shock gene expression has previously been measured by qPCR^22,26,36^, microarray analysis^26^ or RNAseq^22^; methods that are impractical for the analysis of the large number of samples needed to study the heat shock response in more than one tissue of a mouse model over the course of disease (e.g. n ≥ 6/genotype/tissue/age/treatment). This is because qPCR is labour intensive, with the consequence that the number of heat shock genes analysed is generally restricted to no more than three^22,26,36^, and transcriptome-wide approaches are unnecessary to study the induction of a defined number of genes in a large number of samples. To circumvent these limitations, we developed a QuantiGene multiplex assay. QuantiGene technology allows multiplexing of up to 80 genes, the expression of which can be determined directly from tissue lysates, without the need to extract RNA^32,33^. In order to validate the use of QuantiGene, we designed an 8-plex assay that contained *Hsf1* and three heat shock genes: *Hspa1a/b*, *Dnajb1* and *Hspb1*, for which, we had extensive experience of qPCR analysis^22,26,36^. The fold change in the induced expression of these genes was highly comparable between QuantiGene and qPCR.

We next proceeded to design a 16-plex assay that contained the nine heat shock genes that we had previously found to be activated by microarray analysis in mouse brain upon NVP-HSP990 treatment^22,26^, as well as six housekeeping genes. In order to determine the optimal lysate dilution, the median fluorescence intensity (MFI) was measured for each of the genes of interest for serial dilutions of the tissue lysate. This was to ensure that signals were neither saturated nor below the limit of detection at a given lysate dilution. For cost considerations, the dilution series were performed on pooled samples, initially in duplicate, but we now run triplicates to provide tighter data. We found that the lowest dilutions often gave highly variable readings between samples, in which case, these reading were not used in the regression analysis. The basal expression of *Hspa1a/b* is very low, and it was important to ensure that levels were above the limit of detection. None of the heat shock genes or housekeeping genes reached saturation at the dilutions chosen.

Although the zQ175 model had been used to investigate the mechanism underlying the impairment in the heat shock response, a reduction in heat shock gene expression, upon HSF1 activation in zQ175 mice, had not been demonstrated. Our previous qPCR analysis had restricted our investigation of the heat shock response to *Hspa1a/b*, *Dnajb1* and *Hspb1*, and the use of the QuantiGene plex allowed us to expand the study to include *Dnaja1*, *Hspd1*, *Hspe1*, *Hsph1*, *Hsp90aa1* and *Hsp90ab1*. We found clear evidence of an impairment in the induction of seven of the eight heat shock genes in the tibialis anterior of zQ175 mice as compared to wild-type littermates (Fig. 3). To better compare our analysis of the heat shock response in zQ175 brain with that in R6/2 mice, we analysed the heat shock response in brain hemispheres from zQ175 mice at 12 months of age (Fig. 5). In keeping with R6/2 data^26^, the induction of *Hspa1a/b*, *Dnajb1* and *Hspb1* decreased in zQ175 mice as compared to wild type, as well as for five of the additional heat shock genes included on the plex, indicating that the heat shock response is impaired in the zQ175 brain. The pattern of impairment was relatively comparable between the zQ175 striatum at 3 and 12 months (Fig. 4), and the R6/2 striatum at 12 weeks of age (Fig. 8), in both cases including *Hspa1a/b*, *Dnaja1*, *Hspd1*, *Hspe1* and *Hsph1*. Although the extent of induction of four heat shock genes was decreased in R6/2 cortex (Supplementary Fig. S24 online), there was no compelling evidence for a heat shock impairment in this brain region for zQ175 mice (Supplementary Fig. S14 online), which may reflect differences in cortical pathology between these two models. The use of the QuantiGene plex had increased the repertoire of heat shock genes that could be studied, and the induction of *Hspd1*, *Hspe1* and *Hsph1* was consistently found to be impaired in both brain and muscle from zQ175 and R6/2 mice. The fold difference in the induction between the HD and wild-type signals might not represent the extent of an impairment, as induction kinetics differ between heat shock genes and the maximal induction may occur at different times post-dosing.

In order to quantify HSF1 levels by western blotting, we characterised 12 commercial HSF1 antibodies, including the antibodies from Enzo Life Sciences and Bethyl Laboratories used by Gomez-Pastor et al.^31^. In order to be certain that a given antibody detected HSF1, we included lysates from HSF1 knockout mice as a negative control and from NVP-HSP990 treated mice as a positive control, in which activation of HSF1 can be observed as a hypershift on western blots. All antibodies were tested using a panel of brain tissues that had been lysed in four different lysis buffers, using two different blocking solutions and a range of antibody dilutions. The inclusion of our positive and negative controls was crucial. Ten of the antibodies detected multiple proteins, none of which were absent in the HSF1 knockout lysate. This included the Enzo Life Sciences antibody and two of the Bethyl Laboratory antibodies. The only two antibodies that passed the selection criteria and recognised HSF1 in brain and muscle lysates were 51034-1-AP (Proteintech) and A303-176A (Bethyl Laboratories). Both of these antibodies also cross-reacted with other proteins. Using these reagents there was no difference in the basal or activated levels of HSF1 in the zQ175 muscle or brain as compared to wild-type counterparts.

The failure of the proteostasis network to maintain aggregation-prone proteins in their soluble-state is a major pathogenic mechanism underlying HD and other protein-folding diseases, for which the development of therapeutics to boost proteostasis capacity by targeting HSF1, has been proposed^37–39^. However, this strategy is complicated for HD because the ability to activate a heat shock response becomes diminished with disease progression. Understanding the mechanistic basis of this impairment is important for two reasons. First, the inability of a cell to mount a heat shock response would leave it vulnerable to multiple environmental stressors (e.g. viral infection, fever, inflammation) and would be likely to exacerbate the disease process. Second, targeting this impairment would be necessary to complement any therapeutic strategies based on improving protein folding capacity via HSF1 activation. The current study supports our previous work showing that the disease-related attenuation of the heat shock response is not caused by decreased levels of HSF1, but rather through an alternative disease mechanism modulating the ability of HSF1 to activate heat shock gene expression^40,41^. The tetra-acetylation of histone H4 is a strong modulator of HSF1 binding to target genes^40^, and insights into the process by which histone H4 becomes hypoacetylated in HD may shed light on the mechanism underlying the heat shock response impairment.

## Methods

### Mouse breeding and maintenance

All procedures were performed in accordance with the Animals (Scientific Procedures) Act 1986, complied with ARRIVE guidelines and were approved by the University College London Ethical Review Process Committee. zQ175 knockin mice were generated by replacing exon 1 of mouse *Htt* with exon 1 from human *HTT*, carrying a highly expanded CAG repeat^29,30^, from which the neo selectable marker had been removed (delta neo)^42^. These were either bred in-house by backcrossing males to C57Bl/6J females (Charles River) or obtained from the CHDI Foundation colony at the Jackson Laboratory (Bar Harbor, Maine) on a C57Bl/6J background. R6/2 mice^27^ were bred by backcrossing R6/2 males to C57BL/6JOlaHsd × CBA/CaOlaHsd F1 females (B6CBAF1/OlaHsd, Envigo, Netherlands). The wild-type animals used for the QuantiGene validation and the heat shock response kinetics experiments were (CBA/Ca × C57Bl/6J)F1 mice (B6CBAF1/OlaHsd, Envigo). *Hsf1* knockout mice were obtained from the Jackson Laboratory (Bar Harbor, Maine) (stock number 010543; C;129-*Hsf1*^*tm1ljb*^/J^43^. They were maintained by backcrossing *Hsf1*^+*/−*^ heterozygous males to B6CBAF1/OlaHsd females and *Hsf1* knockout mice obtained by intercrossing heterozygous males and females.

Mouse husbandry was as previously described^44^. Within each colony, genetically modified and wild-type mice were group housed with up to five mice per cage, dependent on gender, but genotypes were mixed. Mice were housed in individually ventilated cages with Aspen Chips 4 Premium bedding (Datesand) and with environmental enrichment which included chew sticks and a play tunnel (Datesand). They had unrestricted access to food (Teklad global 18% protein diet, Envigo) and water. The temperature was regulated at 21 °C ± 1 °C and animals were kept on a 12 h light/dark cycle. The animal facility was barrier-maintained and quarterly non-sacrificial FELASA screens found no evidence of pathogens.

### Genotyping and CAG repeat sizing

DNA was extracted from ear biopsy as previously described^45^ and DNA concentration was quantified using a Nanodrop (Thermo Fisher Scientific). R6/2 and zQ175 mice were genotyped as previously described^44^. Genotyping for *Hsf1*: a 25 µL reaction contained 20–50 ng DNA, 1 × GoTaq Flexi Buffer (Promega), 2.0 mM MgCl_2_, 0.2 mM dNTP (Thermo Fisher Scientific), 0.5 µM for each forward primer [5′-AGACCTGTCCTGTGTGCCTAGC and 5′-AGGACATAGCGTTGGCTACCCGT], 0.5 µM for each reverse primer [5′-CAGGTCAACTGCCTACACAGACC and 5′-GCCTGCTATTGTCTTCCCAATCC] and 0.025 U/µL GoTaq2 polymerase. Cycling conditions were 4 min at 95 °C, 35 × (25 s at 95 °C, 20 s at 60 °C, 45 s at 72 °C), 1 min at 72 °C.

CAG repeat sizing for R6/2 and zQ175 mice was performed as previously described^44^. The mean CAG repeat size ± SD for all zQ175 mice used in this study was 198.22 ± 6.39 and for R6/2 mice was 187 ± 1.67.

### NVP-HSP990 formulation and dosing

NVP-HSP990 (2-amino-7,8-dihydro-6H-pyrido[4,3-d]pyrimidin-5-one)^25^ was obtained from Novartis Pharma AG. NVP-HSP990 was formulated as a suspension in 2% methylcellulose (Sigma), diluted in 0.9% saline solution (Severn Biotech) and sonicated twice at high frequency in an ultrasonic bath. Both vehicle and NVP-HSP990 solutions were freshly prepared for every dosing experiment and a single dose (12 mg/kg) was administered by oral gavage. Thorough mixing was carried out between doses to maintain NVP-HSP990 as an even suspension. For all dosing experiments, mice were always randomised with respect to litter of origin and closely age-matched. At the corresponding time points after treatment, mice were sacrificed by a schedule 1 procedure, dissected, tissues were snap-frozen in liquid nitrogen and stored at − 80 °C.

### Antibodies

The source, catalogue number, and antibody dilutions are detailed in Supplementary Table S1 online.

### SDS-PAGE and western blotting

Mouse tissues were lysed in one of the following ice-cold buffers: RIPA (150 mM NaCl, 1% NP40/IGEPAL, 0.5% Na deoxycholate, 0.1% SDS, 50 mM Tris–HCl pH 8.0), HEPES (50 mM HEPES pH 7.0, 150 mM NaCl, 10 mM EDTA, 10% NP40/IGEPAL, 0.5% Na deoxycholate, 0.1% SDS), KCL (50 mM Tris HCl pH 8.0, 10% glycerol, 5 mM EDTA, 150 mM KCl), Triton (50 mM Tris–HCl pH 8.0, 150 mM NaCl, 10% glycerol, 1% Triton X-100, 10 mM EDTA), supplemented with cOmplete protease inhibitors (Roche, 1 mini tablet per 10 mL), 1 mM PMSF (Sigma), 1 mM DTT (Sigma) and in some cases, combinations of PhosSTOP phosphatase inhibitors (Roche), Halt phosphatase inhibitor cocktail (Thermo Fisher Scientific) or 50 mM NaF and 1 mM Na orthovanadate (New England Biolabs). Brain tissues were homogenised using a Polytron homogeniser or motorised pestle. Muscles were homogenised in a Ribolyser at 6.5 m/s for 1 min for four times with a 10 min break between each. After lysis, samples were twice sonicated in a Q125 Sonicator (QSonica) followed by centrifugation for 10 min at 13,000 × *g* at 4 °C. The supernatant was collected, and the protein concentration was quantified via the BCA assay (Thermo Fisher Scientific). 40 μg protein was denatured in Laemmli loading buffer for 5 min at 90 °C. The samples were fractionated using the Criterion system (Bio-Rad) in 10% Criterion TGX precast gels at 100 V until sufficient separation was achieved and then proteins were transferred onto a 0.45 µm nitrocellulose membrane (Bio-Rad) in transfer buffer (25 mM Tris Base, 192 mM glycine, 20% v/v methanol). In some cases, polyacrylamide gels were made in-house as previously described using the Bio-Rad Mini-PROTEAN Tetra-cell system^6^. Membranes were blocked in BSA (Sigma) or non-fat dried milk (Marvel) in concentrations ranging from 0.5 to 5% in TBS (Sigma) for at least 1 h at RT and later incubated with the primary antibodies at selected dilutions in the appropriate blocking solution at 4 °C overnight. The optimised conditions for the Proteintech 51034-1-AP and Bethyl A303-176A antibodies were: tissue homogenisation in ice-cold RIPA buffer with cOmplete protease inhibitor (Roche) and PhosSTOP phosphatase inhibitors (Roche), 1 mM PMSF and 1 mM DTT. Membranes were blocked in 0.5% non-fat dried milk in TBS and antibodies were incubated at 1.0 µg in 6 mL (brain) or 10 mL (tibialis anterior) 0.5% non-fat dried milk in TBS at 4 °C overnight. The ATP5B primary antibody, used as a loading control, was diluted in 0.5% non-fat dried milk in TBS at 1:50,000 dilution and membranes were incubated for approximately 2–3 h at RT. In all cases, blots were washed at least three times for 10 min in 0.02% Tween-TBS (TBS-T) and then incubated with secondary antibodies in 0.02% TBS-T for 1 h at RT. After washes, the blots were exposed using Clarity Western ECL Substrate (Bio-Rad) in a ChemiDoc system (Bio-Rad) and later quantified with Image Lab software (Bio-Rad).

### RNA isolation, cDNA synthesis and qPCR

Mouse brain and muscle samples were homogenised in Qiazol lysis buffer (Qiagen) as per the manufacturer’s recommendations via a Polytron homogeniser probe (brain samples) or Ribolyser instrument for three times at 6.5 m/s for 1 min, placing samples on ice for 5 min between ribolysations (muscle samples). RNA was extracted and cDNA synthesised as previously published^46^ and quantified using a Nanodrop (Thermo Fisher Scientific). The primers and probes for the qPCR reactions are presented in Supplementary Table S3 online and qPCR was performed as previously described^46^ and data normalised to reference genes following the 2^−ΔΔCt^ method^47^.

### Tissue homogenisation and QuantiGene gene expression assays

Samples for QuantiGene experiments were homogenised using Polytron homogeniser, motorised pestle (for brain regions) or using liquid nitrogen and pre-chilled pestle and mortar (for muscle) using the QuantiGene reagents from Thermo Fisher Scientific, following the manufacturer’s recommendations and as previously described^46^ using the dilutions listed in Supplementary Table S2 online. Information pertaining to probe regions and accession numbers for the QuantiGene multiplex assays used in this study can be found in Supplementary Tables S4 and S5 online. The median fluorescent intensity (MFI) was read in a Magpix (Luminex) using the xPonent software.

### Statistical analysis

Data were screened for outliers using the ROUT test (GraphPad Prism v7) and outliers were removed from the analysis. Statistical analysis was performed with SPSS (v26) using either an unpaired Student’s *t* test or two‐way ANOVA with Bonferroni post hoc test. Graphs were prepared using GraphPad Prism (v7). *P* values less than 0.05 were considered statistically significant.

## Supplementary Information

Supplementary Information.

## Data Availability

The datasets generated and/or analysed during the current study are available from the corresponding author on reasonable request.
