# Supplementary material for: The heat shock response, determined by QuantiGene multiplex, is impaired in HD mouse models and not caused by HSF1 reduction
Source: Sci Rep. 2021 Apr 27;11:9117. doi: 10.1038/s41598-021-88715-5 (PMC8079691; doi:10.1038/s41598-021-88715-5)
Supplement: Supplementary file 1 — Supplementary Information. [file 41598_2021_88715_MOESM1_ESM.pdf]

The heat shock response, determined by QuantiGene multiplex, is impaired in HD mouse models and not caused by HSF1 reduction

Casandra Gomez-Paredes[1], Michael A. Mason[1], Bridget A. Taxy[1],

Aikaterini S. Papadopoulou[1], Paolo Paganetti[2] and Gillian P. Bates[1]*

[1]Huntington's Disease Centre, Department of Neurodegenerative Disease and UK Dementia Research Institute at UCL, Queen Square Institute of Neurology, University College London, London WC1N 3BG, UK.

[2]Laboratory for Biomedical Neurosciences, Neurocenter of Southern Switzerland, Ente Ospedaliero Cantonale and Faculty of Biomedical Sciences, Università della Svizzera Italiana, Lugano, Switzerland.

*Corresponding Author: gillian.bates@ucl.ac.uk

Supplementary Information

Supplementary Table S1. List of antibodies used and tested in the study.

Supplier	Antibody	Catalogue number	Dilution / concentration used	Other dilutions tested	Host	Primary or secondary
Bethyl Laboratories	HSF1	A303-176A	0.16 µg/mL	-	Rabbit polyclonal	Primary
Proteintech	HSF1	51034-1-AP	0.16 µg/mL<br>0.1 µg/mL (TA)	-	Rabbit polyclonal	Primary
Santa Cruz	HSF1 (10H8)	sc-13516	-	1:200-1:500	Rat monoclonal	Primary
Cell Signaling Technology	HSF1	#4356T	-	1:1000	Rabbit polyclonal	Primary
Invitrogen	HSF1	PA3-017	-	1:1000-1:2000	Rabbit polyclonal	Primary
Santa Cruz	HSF1 (E-4)	sc-17757	-	1:500-1:1000	Mouse monoclonal	Primary
Abcam	HSF1 (10H8)	ab61382	-	1:1000-1:2000	Rat monoclonal	Primary
Abcam	HSF1	ab2923	-	1:500	Rabbit polyclonal	Primary
Bethyl Laboratories	HSF1	A303-174AT	-	1:2000-1:3000	Rabbit polyclonal	Primary
Bethyl Laboratories	HSF1	A303-175AT	-	1:1000	Rabbit polyclonal	Primary
Enzo Life Sciences	HSF1	ADI-SPA-901-F	-	1:1000-1:2000	Rabbit polyclonal	Primary
Enzo Life Sciences	HSF1 (10H8)	ADI-SPA-950-F	-	1:1000-1:2000	Rat monoclonal	Primary
Abcam	ATP5B	ab14730	1:50000	-	Mouse monoclonal	Primary
Abcam	Anti-rat IgG H&L (HRP)	ab6734	1:2000	-	Rabbit polyclonal	Secondary
Agilent Dako	Anti-mouse IgG/HRP	P 0447	1:2000	-	Goat polyclonal	Secondary
Agilent Dako	Anti-rabbit IgG/HRP	P 0448	1:5000	-	Goat polyclonal	Secondary

TA = tibialis anterior

Supplementary Figure S1. Optimisation of commercial HSF1 antibodies

a) Representative western blots of ab2923, ab61382, Bethyl A303-174AT and Bethyl A303-175AT against lysates prepared in various lysis buffers as indicated. The predicted size of the HSF1 band as detected by these antibodies was: 83 kDa (ab2923), 57 kDa (ab61382), ~70 kDa (Bethyl A303-174AT) and ~70 kDa (Bethyl A303-175AT). All antibodies detected multiple proteins, none of which was absent in the HSF1 knockout lanes. None of these antibodies detected HSF1.

b) Representative western blots of Cell Signalling #4356T and Invitrogen PA3-017 against lysates prepared in various lysis buffers as indicated. The predicted size of the HSF1 band as detected by these antibodies was: ~80 kDa (Cell Signalling #4356T) and 83 kDa (Invitrogen PA3-017). The signals were weak, and the Invitrogen PA3-017 antibody detected multiple proteins. None of the bands were absent in the HSF1 knockout lanes. These antibodies did not detect HSF1.

c) Representative western blots of sc-17757 and sc-13516 against lysates prepared in various lysis buffers as indicated. The predicted size of the HSF1 band as detected by these antibodies was: 89-90 kDa (sc-17757) and 89-90 kDa (sc-13516). The sc-17757 antibody detected multiple proteins. None of the bands were absent in the HSF1 knockout lanes. The sc-13516 antibody appeared to detect a hypershift in lysates from mice that had been treated with NVP-HSP990, although a band was present in the HSF1 knockout lane. Therefore, this antibody was investigated using in-house and precast gels, but the HSF1 band in the HSF1 knockout lanes persisted and this antibody was discarded.

d) Representative western blots of Enzo-ADI-SPA-901 and Enzo-ADI-SPA-950 against lysates prepared in various lysis buffers as indicated. The predicted size of the HSF1 band as detected by these antibodies was: 80-95 kDa (Enzo-ADI-SPA-901) and ~85 kDa (Enzo-ADI-SPA-950). Both antibodies appeared to detect a hypershift in lysates from mice that had been treated with NVP-HSP990, although a band was present in the HSF1 knockout lanes. Therefore, these antibodies were investigated using in-house and precast gels, but the HSF1 bands in the HSF1 knockout lanes persisted and these antibodies were discarded.

990 = NVP-HSP990; het = heterozygous; WT = wild-type; KO = knockout; Na orth = Na orthovanadate; Halt inhib = Halt phosphatase inhibitor cocktail (Thermo Fisher Scientific)

a)

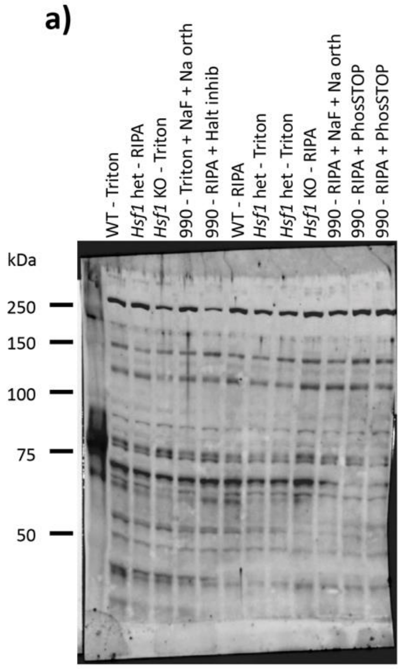

ab2923 (Abcam)
Diluted 1:500 in 2.5 % non-fat milk
Nitrocellulose membrane
Precast gel

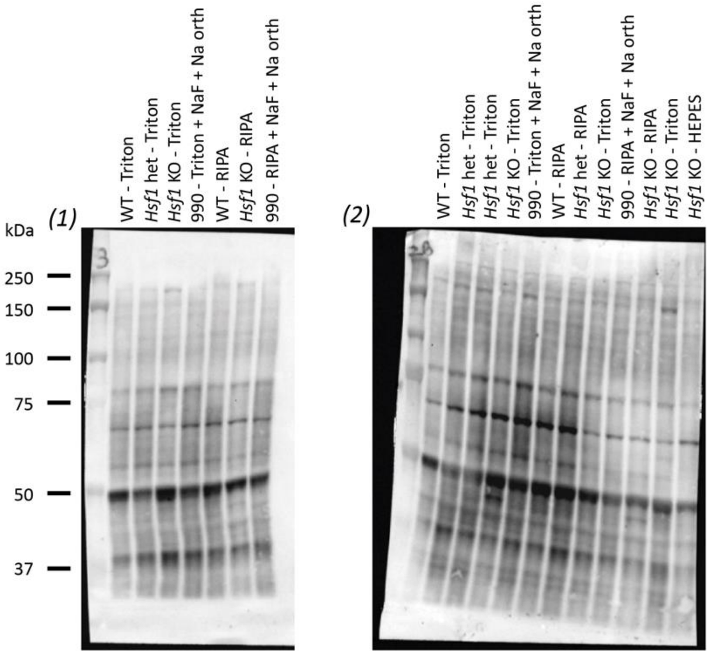

ab61382 (Abcam)
(1) Diluted 1:1000 in 5 % non-fat milk
(2) Diluted 1:1000 in 2.5 % BSA
Nitrocellulose membranes
Precast gels

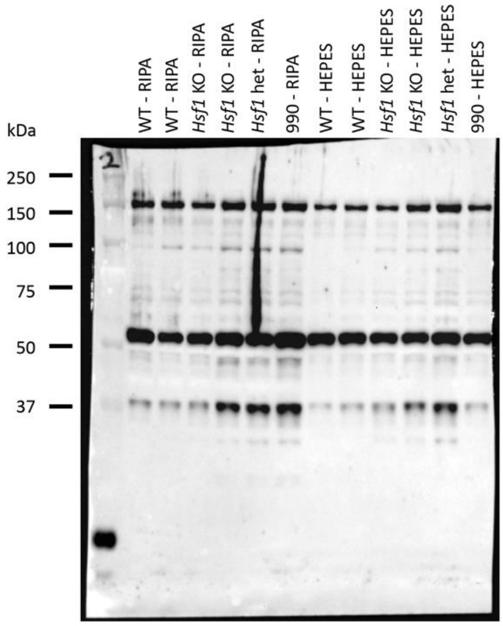

A303-174AT (Bethyl)
Diluted 1:2000 in 5 % non-fat milk
Nitrocellulose membrane
Precast gel

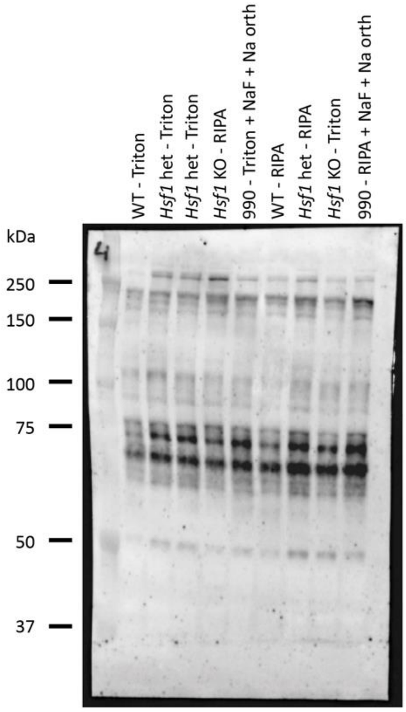

A303-175AT (Bethyl)
Diluted 1:1000 in 2.5 % non-fat milk
Nitrocellulose membrane
Precast gel

b)

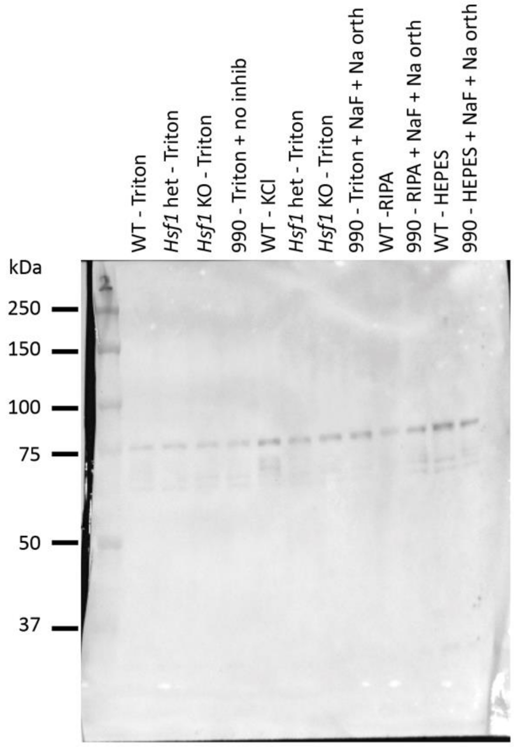

Cell Signalling #4356T
Diluted 1:1000 in 5 % non-fat milk
Nitrocellulose membrane
Precast gel

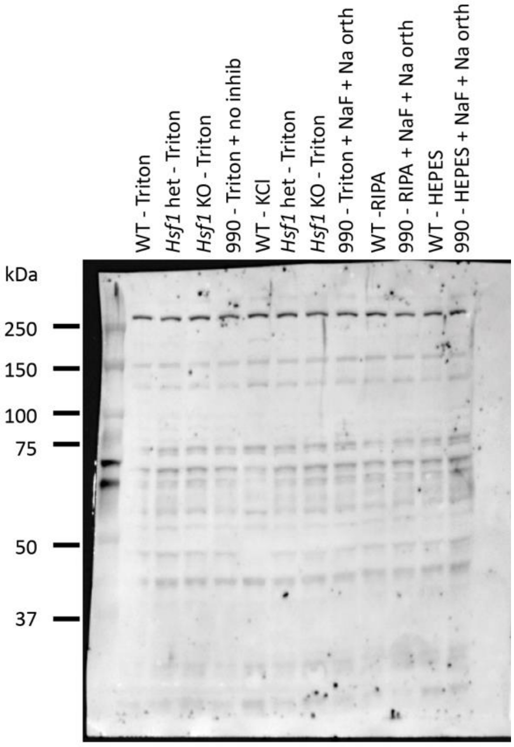

Invitrogen PA3-017
Diluted 1:2000 in 5 % non-fat milk
Nitrocellulose membrane
Precast gel

c)

sc-17757 (Santa Cruz)

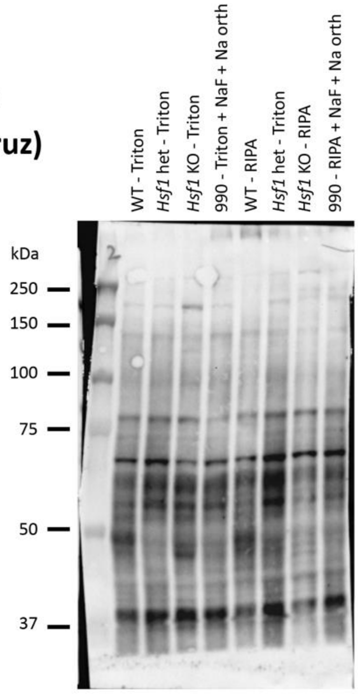

1:500 in 5 % milk
Nitrocellulose membrane
Precast gel

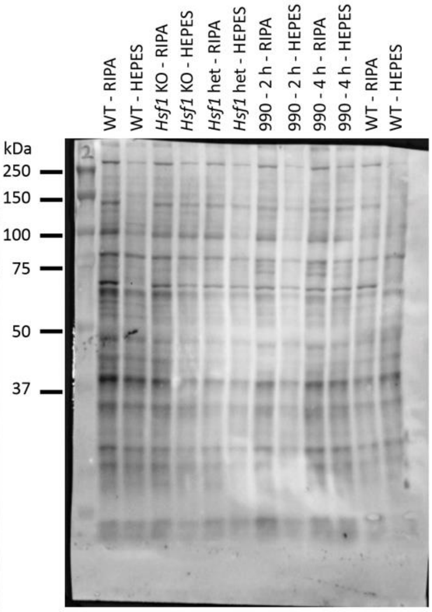

1:1000 in 3 % BSA
Nitrocellulose membrane
Precast gel

sc-13516 (Santa Cruz)

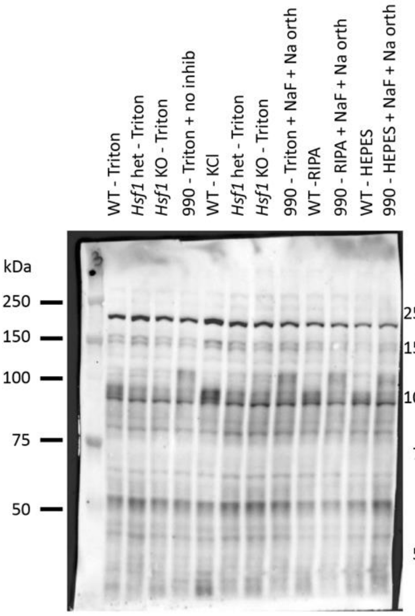

1:200 in 5 % milk
Nitrocellulose membrane
Precast gel

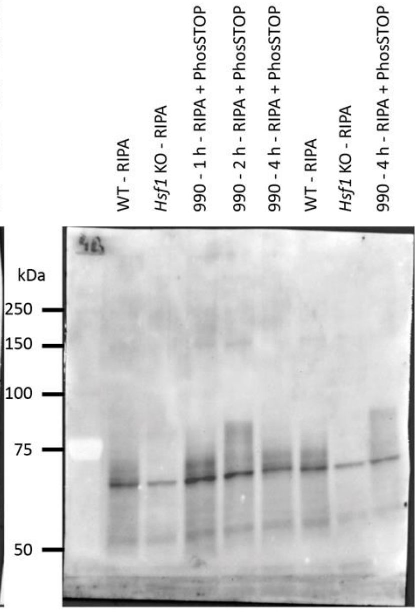

1:400 in 0.5 % milk
Nitrocellulose membrane
Precast gel

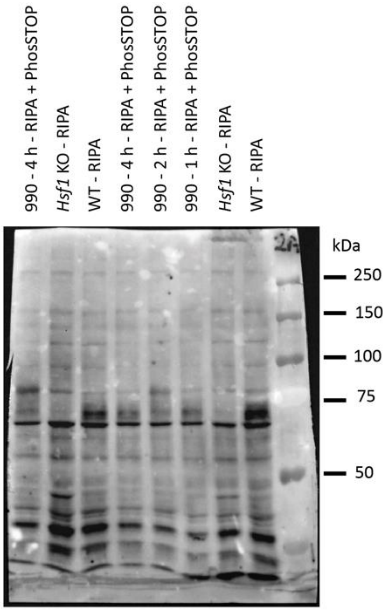

1:400 in 0.5 % milk
Nitrocellulose membrane
In-house made gel

d)

Enzo Life Sciences - ADI-SPA-901-F

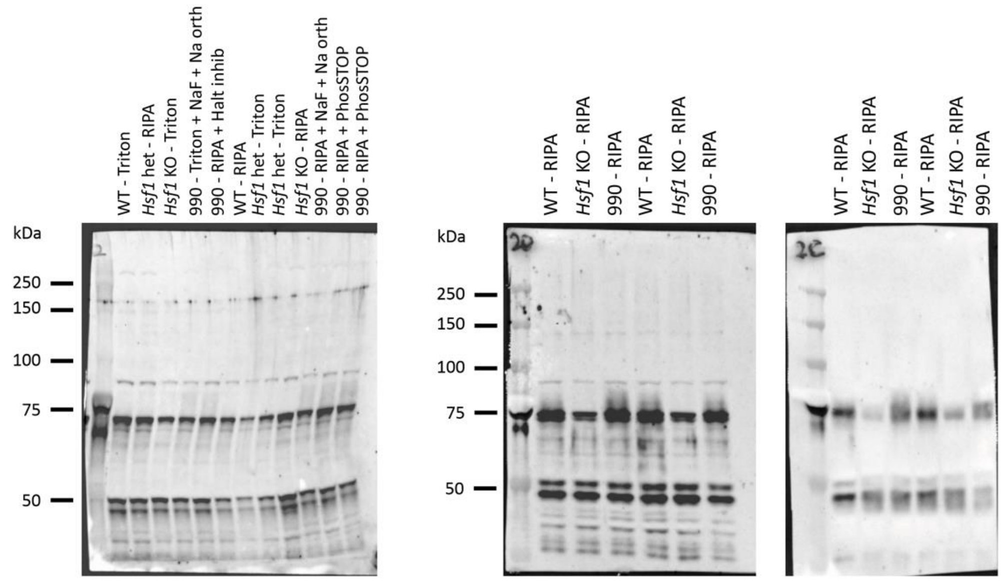

1:1500 in 2.5 % milk
Nitrocellulose membrane
Precast gel

1:2000 in 0.5 % milk
Nitrocellulose membrane
In-house made gel

1:2000 in 0.5 % milk
Nitrocellulose membrane
Precast gel

Enzo Life Sciences - ADI-SPA-950-F

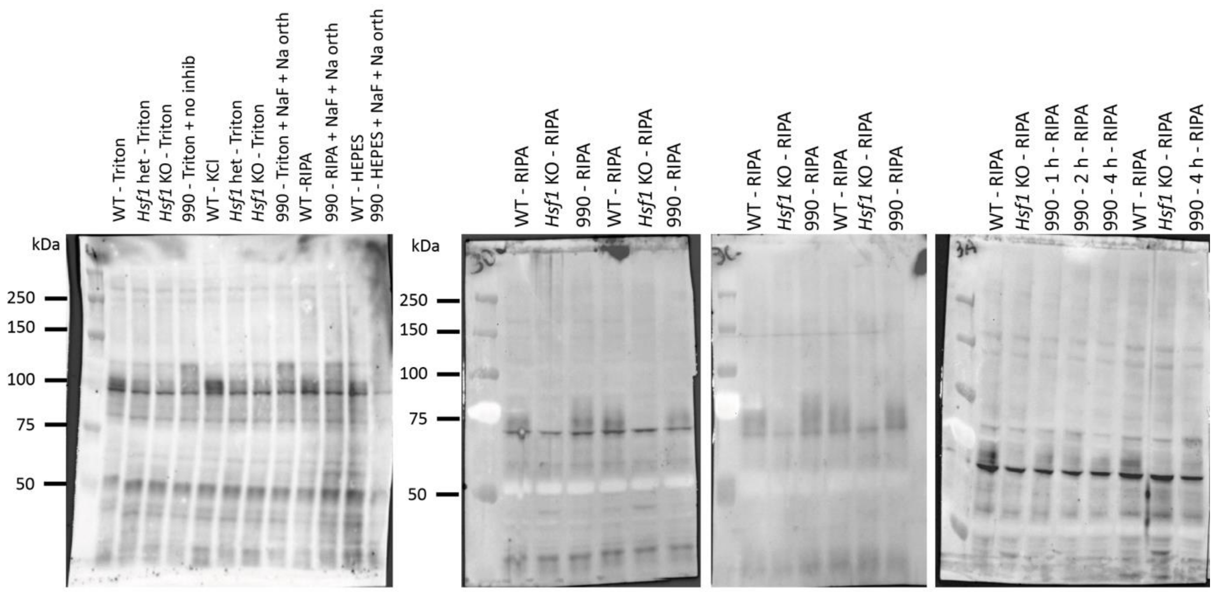

1:1000 in 5 % milk
Nitrocellulose membrane
Precast gel

1:2000 in 0.5 % milk
Nitrocellulose membrane
In-house made gel

1:2000 in 0.5 % milk
Nitrocellulose membrane
Precast gel

1:2000 in 0.5 % milk
Nitrocellulose membrane
In-house made gel

Supplementary Figure S2.

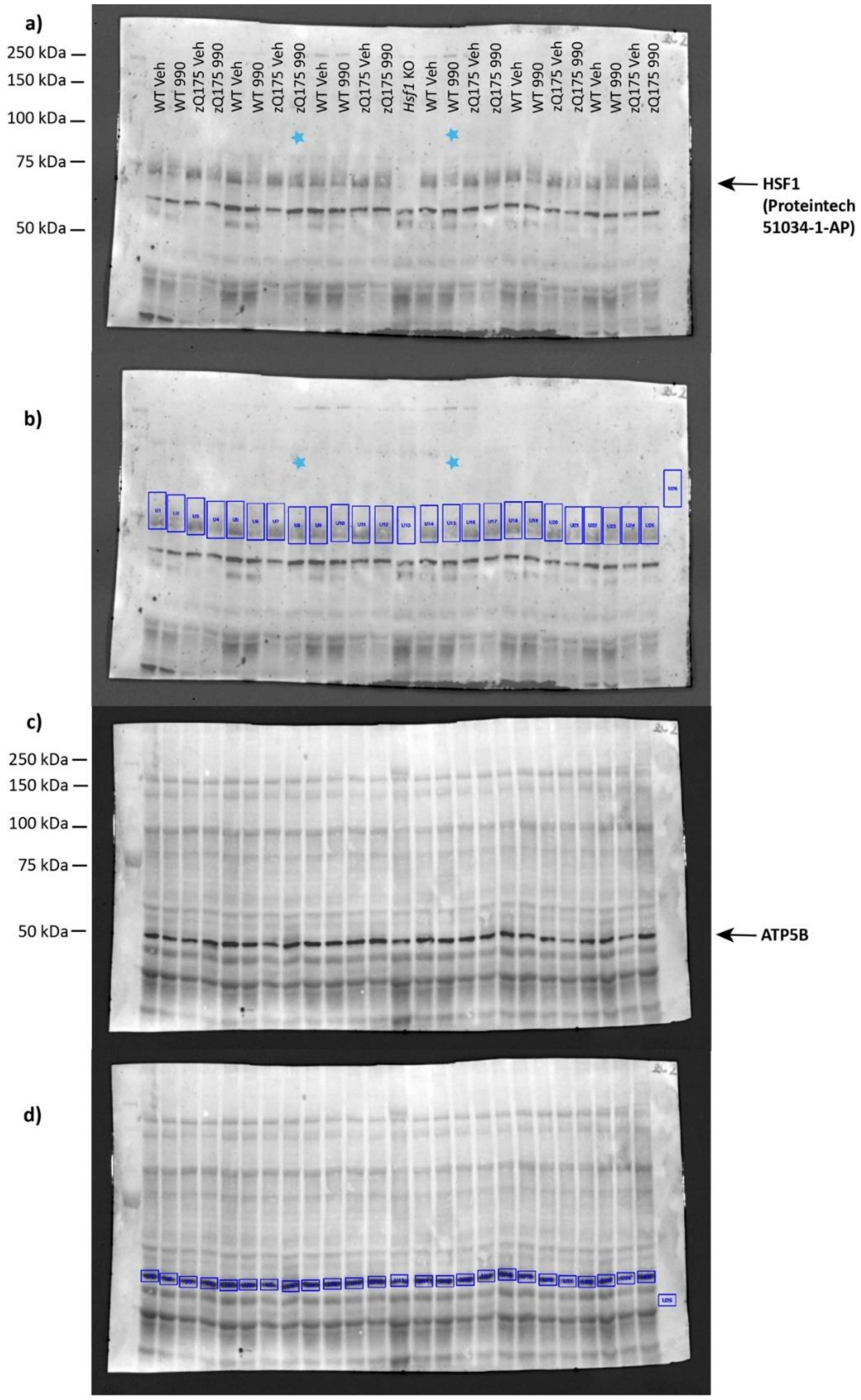

Supplementary Figure S2. Full-length western blots of HSF1 in tibialis anterior
Western blot (a) immunoprobed with HSF1 Proteintech 51034-1-AP antibody and (b) regions selected for quantification from the HSF1 antibody probed blot; (c) immunoprobed with ATP5B and (d) regions selected for quantification from the ATP5B probed blot. *Samples excluded from quantification due to poor induction of the heat shock response, as explained in figure legend of Fig. 3. Veh = vehicle, 990 = NVP-HSP990; WT = wild-type.

Supplementary Figure S3.

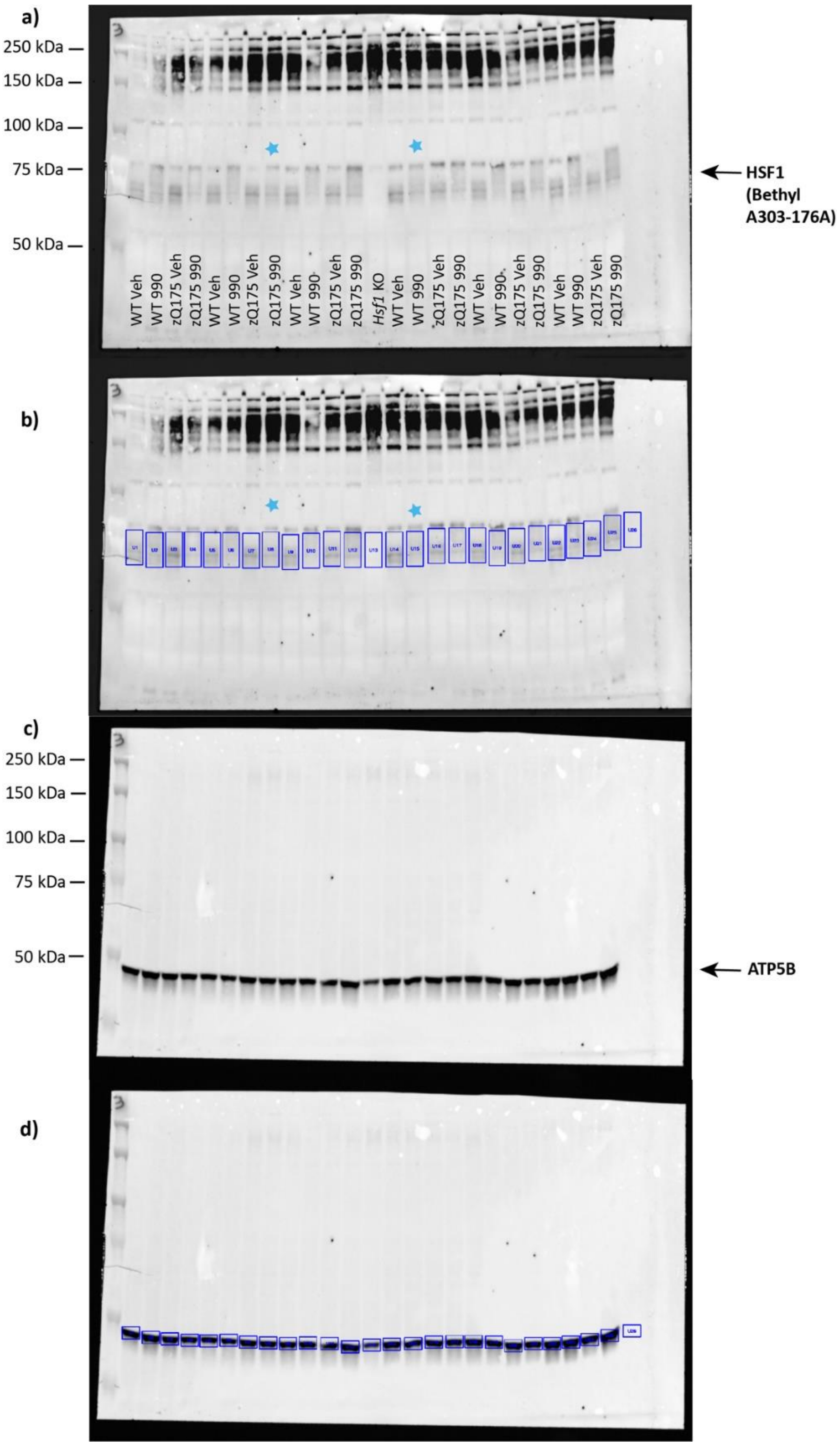

Supplementary Figure S3. Full-length western blots of HSF1 in striatum

Western blots (a) immunoprobed with HSF1 Bethyl A303-176A antibody and (b) regions selected for quantification from the HSF1 antibody probed blot; (c) immunoprobed with ATP5B and (d) regions selected for quantification from the ATP5B probed blot. *Samples excluded from quantification due to poor induction of the heat shock response, as explained in figure legend of Fig. 4. Veh = vehicle, 990 = NVP-HSP990; WT = wild-type.

Supplementary Figure S4.

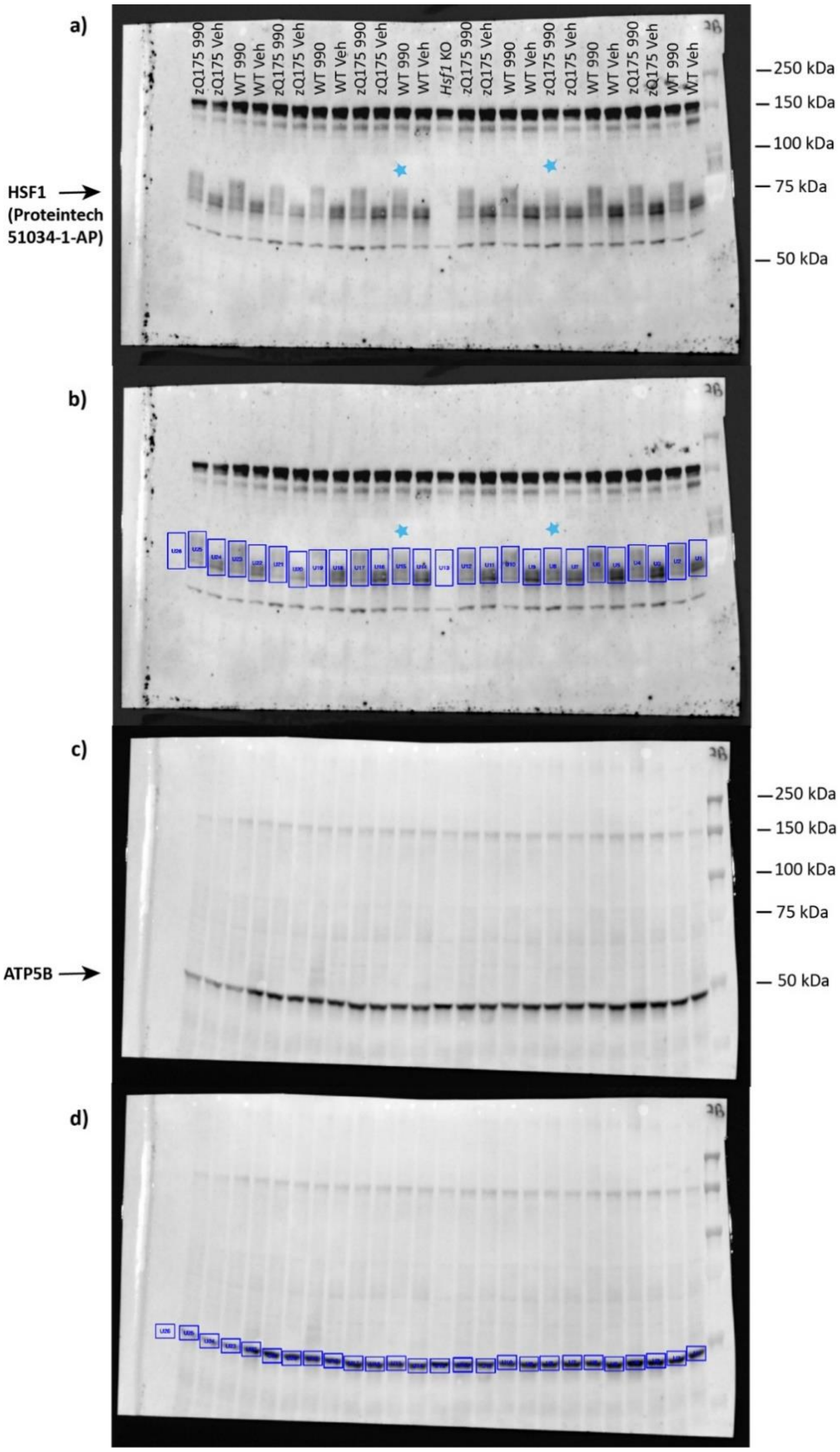

Supplementary Figure S4. Full-length western blots of HSF1 in cortex
Western blots (a) immunoprobed with HSF1 Proteintech 51034-1-AP antibody and (b) regions selected for quantification from the HSF1 antibody probed blot; (c) immunoprobed with ATP5B and (d) regions selected for quantification from the ATP5B probed blot. *Samples excluded from quantification due to poor induction of the heat shock response, as explained in figure legend of Supplementary Fig. S14. Veh = vehicle, 990 = NVP-HSP990; WT = wild-type.

Supplementary Table S2. Tissue lysate dilutions used for QuantiGene multiplex assays.

QuantiGene assay	Tissue	Dilution of starting material (10 mg/300 μL)	Final input (μg/μL)
**16-plex**	Cortex	1:6	5.5
	Striatum	1:4	8.3
	Tibialis anterior	1:4	8.3
	Brain hemispheres	1:9	3.7
**8-plex**	Brain hemispheres	1:9	3.7
	Tibialis anterior	1:10	3.3

Supplementary Table S3. TaqMan assays from Thermo Fisher Scientific or designed in-house.

Type	Gene Symbol	Gene name	Accession Number	TaqMan assay ID (Thermo Fisher Scientific)
HK	*Canx*	*Calnexin*	*NM_007597*	*Mm00500330_m1*
HK	*Rpl13a*	*Ribosomal Protein L13a*	*NM_009438*	*Mm01612987_g1*
HK	*Atp5b*	*ATP synthase subunit Beta*	*NM_016774*	*Mm00443967_g1*
HK	*Eif4a2*	*Eukaryotic translation initiation factor 4A2*	*NM_013506*	*Mm01730183_gH*
GOI	*Hspa1a/b*	*Heat shock protein 1A/B*	*NM_010479*	*Mm01159846_s1*
GOI	*Hspb1*	*Heat shock protein 1*	*NM_013560*	*Mm00834384_g1*
GOI	*Dnajb1*	*DnaJ (Hsp40) homolog, subfamily B, member 1*	*NM_018808*	*Mm00444519_m1*

Type	Gene Symbol	Gene name	ID	Sequence (5' - 3')	Supplier
GOI	*Hsf1*	*Heat shock factor 1*	**Forward primer**	*CGAGTGGGAACAGCTTCCA*	*Primers/probes designed in-house*
			**Reverse primer**	*ACTTGGGCAGCACCTCCTT*	
			**Probe**	*TTTGACCAGGGCCAGTT*	

GOI = Gene of interest; HK = Housekeeping gene

Supplementary Table S4. Probes in the QuantiGene 8-plex assay

Type	Gene Symbol	Gene name	Accession Number	Probe set region
HK	*Canx*	*Calnexin*	*NM_007597*	*76-727*
HK	*Rpl13a*	*Ribosomal Protein L13a*	*NM_009438*	*2-467*
HK	*Atp5b*	*ATP synthase subunit Beta*	*NM_016774*	*22-409*
HK	*Eif4a2*	*Eukaryotic translation initiation factor 4A2*	*NM_013506*	*710-1271*
GOI	*Hsf1*	*Heat shock factor 1*	*NM_008296*	*1712-2263*
GOI	*Hspa1a/b*	*Heat shock protein 1A/B*	*NM_010479*	*2186-2721*
GOI	*Hspb1*	*Heat shock protein 1*	*NM_013560*	*103-555*
GOI	*Dnajb1*	*DnaJ (Hsp40) homolog, subfamily B, member 1*	*NM_018808*	*569-1125*

GOI = Gene of interest; HK = Housekeeping gene

Supplementary Figure S5.

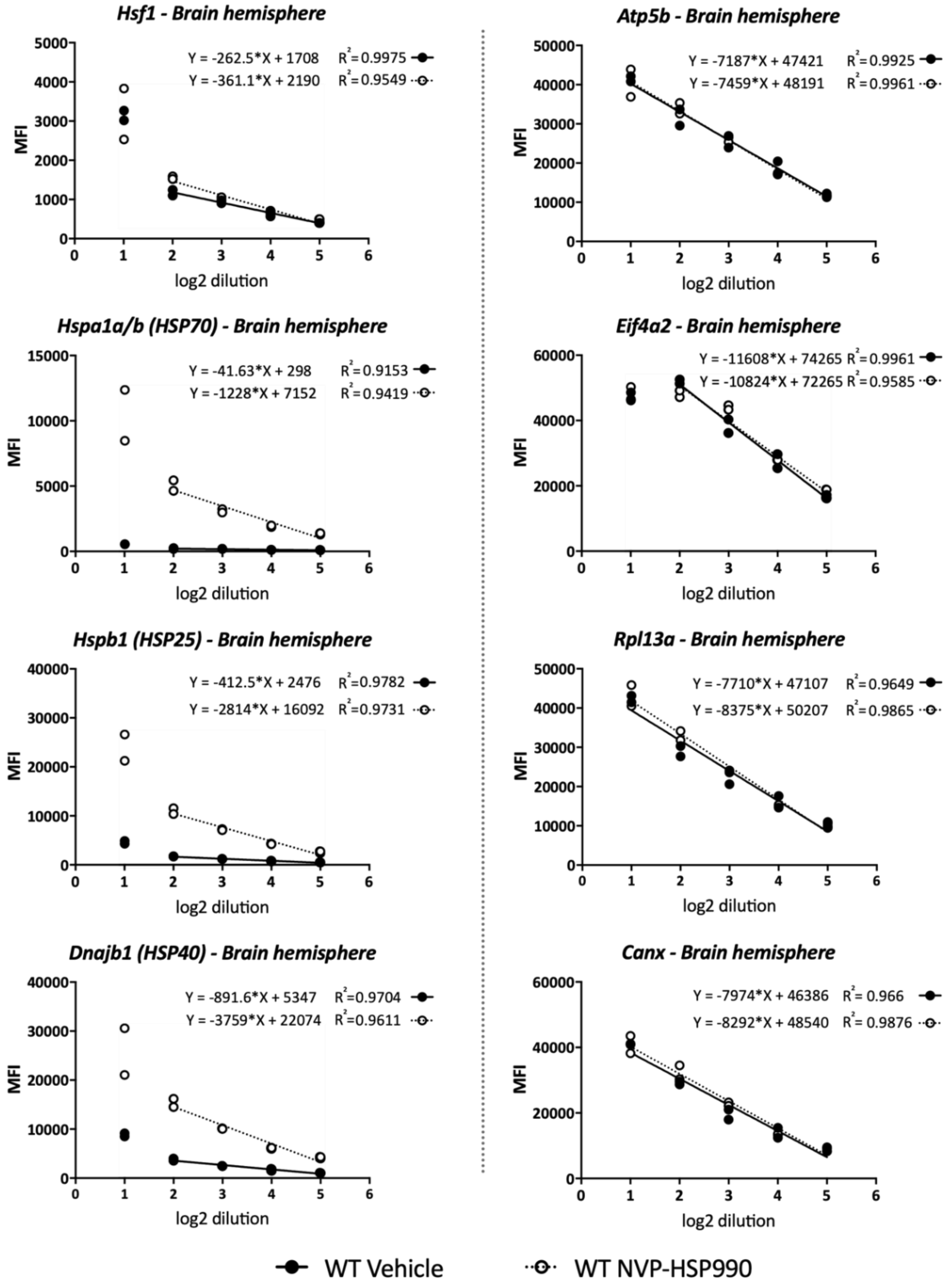

Supplementary Figure S5. Optimisation of the QuantiGene 8-plex assay for use with wild-type brain tissue

Brain samples were pooled (*n* = 5-6 / treatment), subjected to a two-fold serial dilution and analysed in duplicate. The MFI signals for the corresponding brain lysate dilutions from vehicle or NVP-HSP990 treated wild-type mice are shown. The genes of interest (left-hand side) have linear regression lines with different slopes for the three heat shock genes. The expression of *Eif4a2* is saturated at the 1:2 dilution, otherwise, the housekeeping genes (right-hand side) are stable between the two treatment groups. MFI = median fluorescent intensity, WT = wild-type.

Supplementary Figure S6.

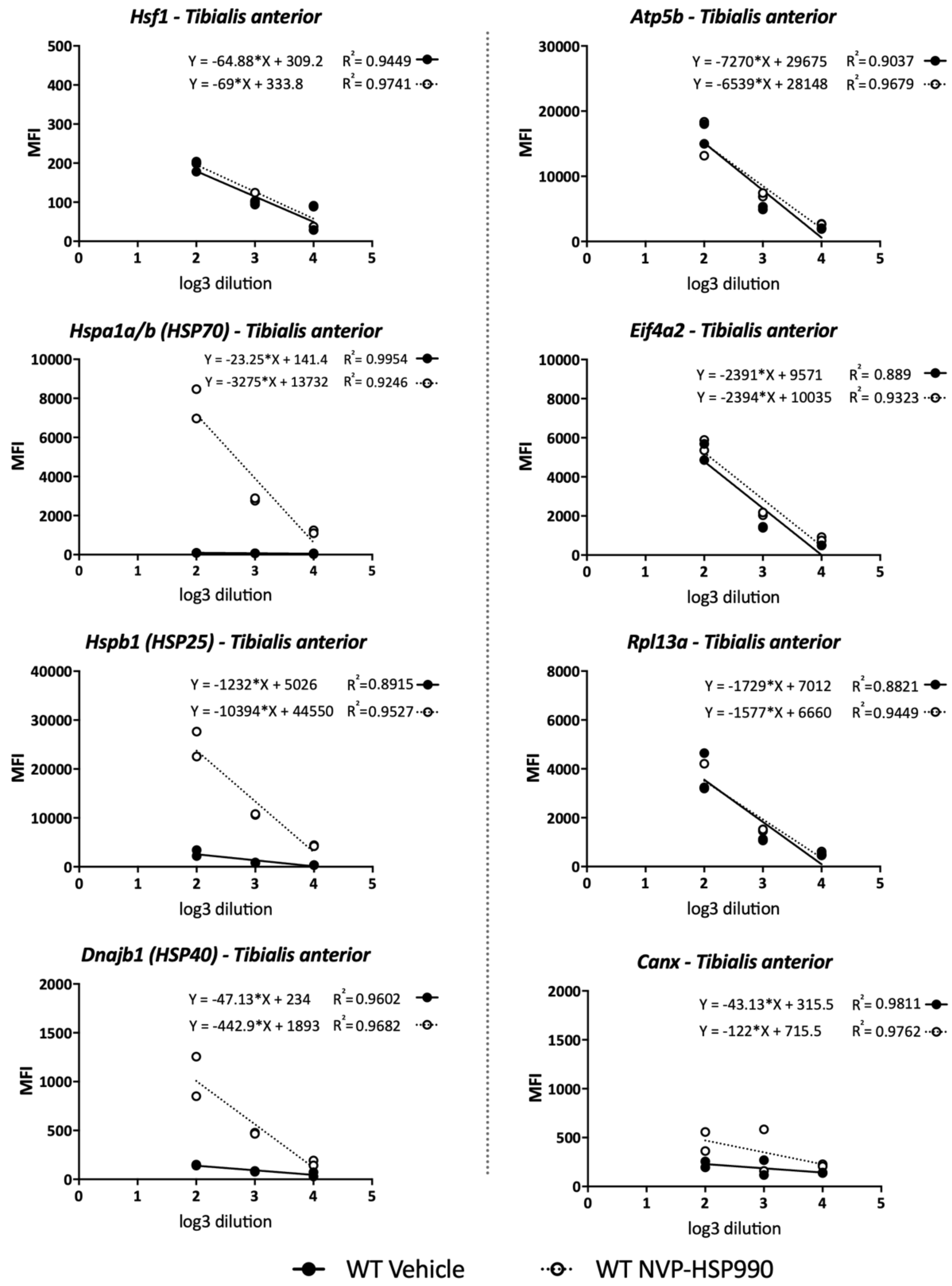

Supplementary Figure S6. Optimisation of the QuantiGene 8-plex assay for use with wild-type muscle tissue

Tibialis anterior samples were pooled (*n* = 5-6 / treatment), subjected to a three-fold serial dilution and analysed in duplicate. The MFI signals for the corresponding muscle lysate dilutions from vehicle or NVP-HSP990 treated wild-type mice are shown. The genes of interest (left-hand side) have linear regression lines with different slopes for the three heat shock genes. The expression of the housekeeping genes (right-hand side) is stable between the two treatment groups. MFI = median fluorescent intensity, WT = wild-type.

Supplementary Figure S7.

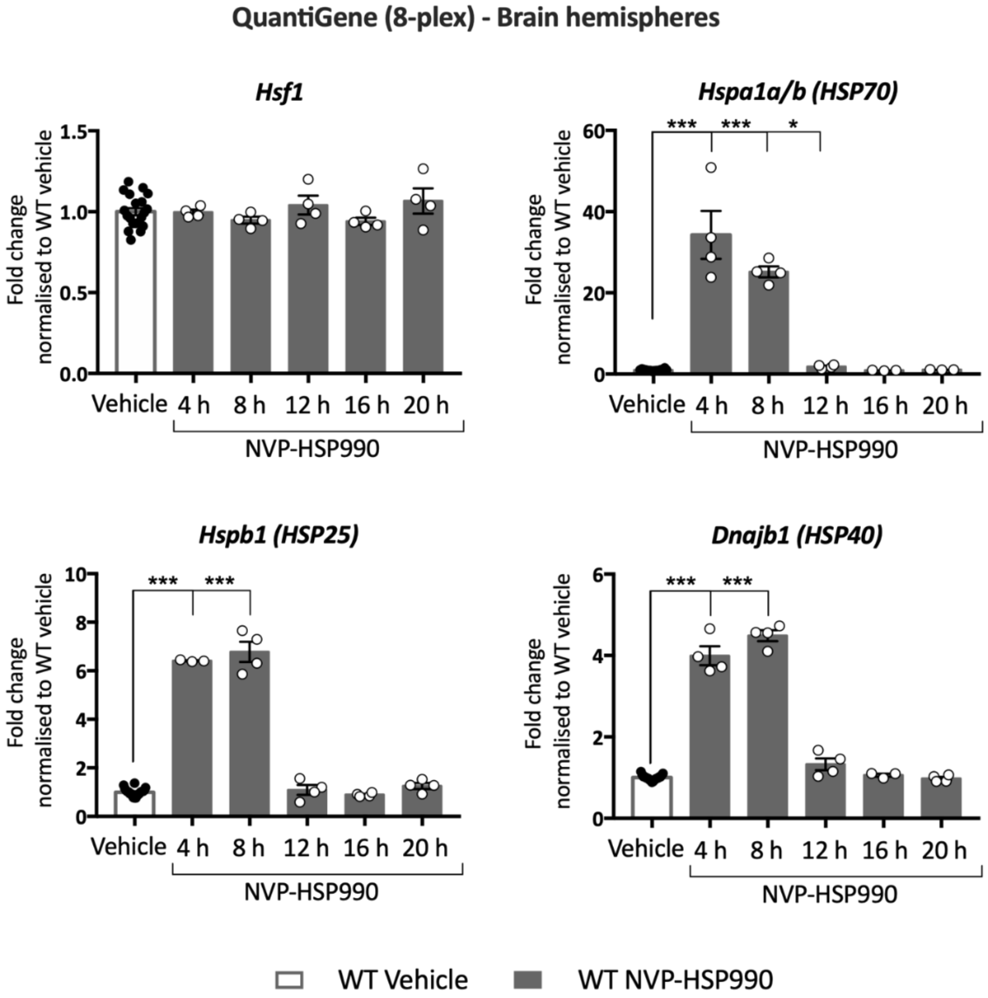

Supplementary Figure S7. Kinetics of the pharmacological induction of the heat shock response with NVP-HSP990

The expression of *Hsf1*, *Hspa1a/b*, *Hspb1* and *Dnajb1* in brain hemispheres from wild-type mice was measured with the QuantiGene 8-plex assay. These hemispheres had been collected at 4, 8, 12, 16 and 20 hours after treatment with either vehicle or NVP-HSP990. All vehicle samples from all time points were plotted together in the graphs for each gene. The expression of the heat shock genes was significantly increased by 4 hours post-dosing with NVP-HSP990 and maintained to 8 hours. By 12 hours (with exception of *Hspa1a/b*), the expression had returned to baseline. n = 3-4 / treatment / time point. Statistical analysis was by unpaired Student's t-test. Mean ± SEM. *** $p \leq 0.001$, * $p \leq 0.05$. Test statistical values can be found in Supplementary Table S8 online. WT = wild-type.

Supplementary Table S5. Probes in the QuantiGene 16-plex assay

Type	Gene Symbol	Gene name	Accession Number	Probe set region
HK	*Canx*	*Calnexin*	*NM_007597*	*1195-1720*
HK	*Rpl13a*	*Ribosomal Protein L13a*	*NM_009438*	*2-467*
HK	*Atp5b*	*ATP synthase subunit Beta*	*NM_016774*	*22-409*
HK	*Eif4a2*	*Eukaryotic translation initiation factor 4A2*	*NM_013506*	*710-1271*
HK	*Sdha*	*Succinate dehydrogenase complex flavoprotein subunit A*	*NM_023281*	*76-727*
HK	*Gapdh*	*Glyceraldehyde 3-phosphate dehydrogenase*	*NM_001001303*	*735-1001*
GOI	*Hsf1*	*Heat shock factor 1*	*NM_008296*	*1712-2263*
GOI	*Hspa1a/b*	*Heat shock protein 1A/B*	*NM_010479*	*2186-2721*
GOI	*Hspb1*	*Heat shock protein 1*	*NM_013560*	*103-555*
GOI	*Dnaja1*	*DnaJ (Hsp40) homolog, subfamily A, member 1*	*NM_008298*	*474-1101*
GOI	*Dnajb1*	*DnaJ (Hsp40) homolog, subfamily B member 1*	*NM_018808*	*569-1125*
GOI	*Hspd1*	*Heat shock protein 1 (chaperonin)*	*NM_010477*	*1732-2170*
GOI	*Hspe1*	*Heat shock protein 1 (chaperonin 10)*	*NM_008303*	*259-657*
GOI	*Hsph1*	*Heat shock protein 105*	*NM_013559*	*1985-2430*
GOI	*Hsp90aa1*	*Heat shock protein 90, alpha (cytosolic), class A member 1*	*NM_010480*	*245-989*
GOI	*Hsp90ab1*	*Heat shock protein 90, alpha (cytosolic), class B member 1*	*NM_008302*	*1039-1542*

GOI = Gene of interest; HK = Housekeeping gene

Supplementary Figure S8. Optimisation of the QuantiGene 16-plex assay based on the MFI signals of the housekeeping genes for use with zQ175 and wild-type tibialis anterior at 3 months and 20 months of age

Tibialis anterior samples were pooled (*n* = 6 / genotype / treatment / age) and subjected to a two-fold serial dilution and analysed in duplicate. The MFI signals for the corresponding muscle lysate dilutions from vehicle or NVP-HSP990-treated zQ175 and wild-type mice are shown. MFI = median fluorescent intensity, WT = wild-type; m = months.

Supplementary Figure S9. Optimisation of the QuantiGene 16-plex assay based on the MFI signals of the housekeeping genes for use with zQ175 and wild-type striatum at 3 months and 20 months of age

Striatal samples were pooled (*n* = 6 / genotype / treatment / age) and subjected to a two-fold serial dilution and analysed in duplicate. The MFI signals for the corresponding striatal lysate dilutions from vehicle or NVP-HSP990-treated wild-type mice are shown. MFI = median fluorescent intensity, WT = wild-type; m = months.

Supplementary Figure S10. Optimisation of the QuantiGene 16-plex assay based on the MFI signals of the housekeeping genes for use with zQ175 and wild-type cortex at 3 months and 20 months of age

Cortical samples were pooled (*n* = 6 / genotype / treatment / age) and subjected to a two-fold serial dilution and analysed in duplicate. The MFI signals for the corresponding cortical lysate dilutions from vehicle or NVP-HSP990-treated wild-type mice are shown. MFI = median fluorescent intensity, WT = wild-type; m = months.

Supplementary Figure S8.

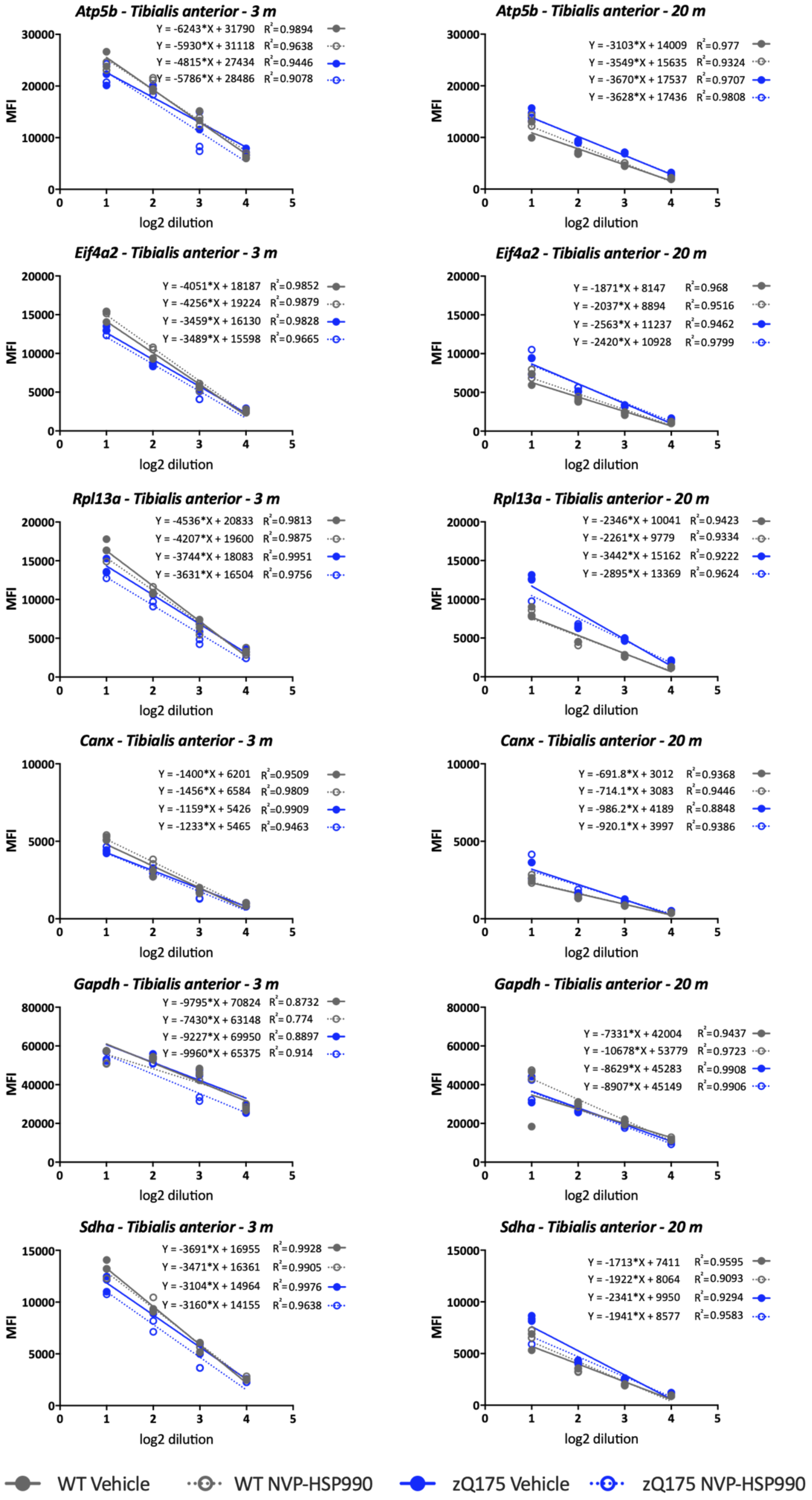

Supplementary Figure S9.

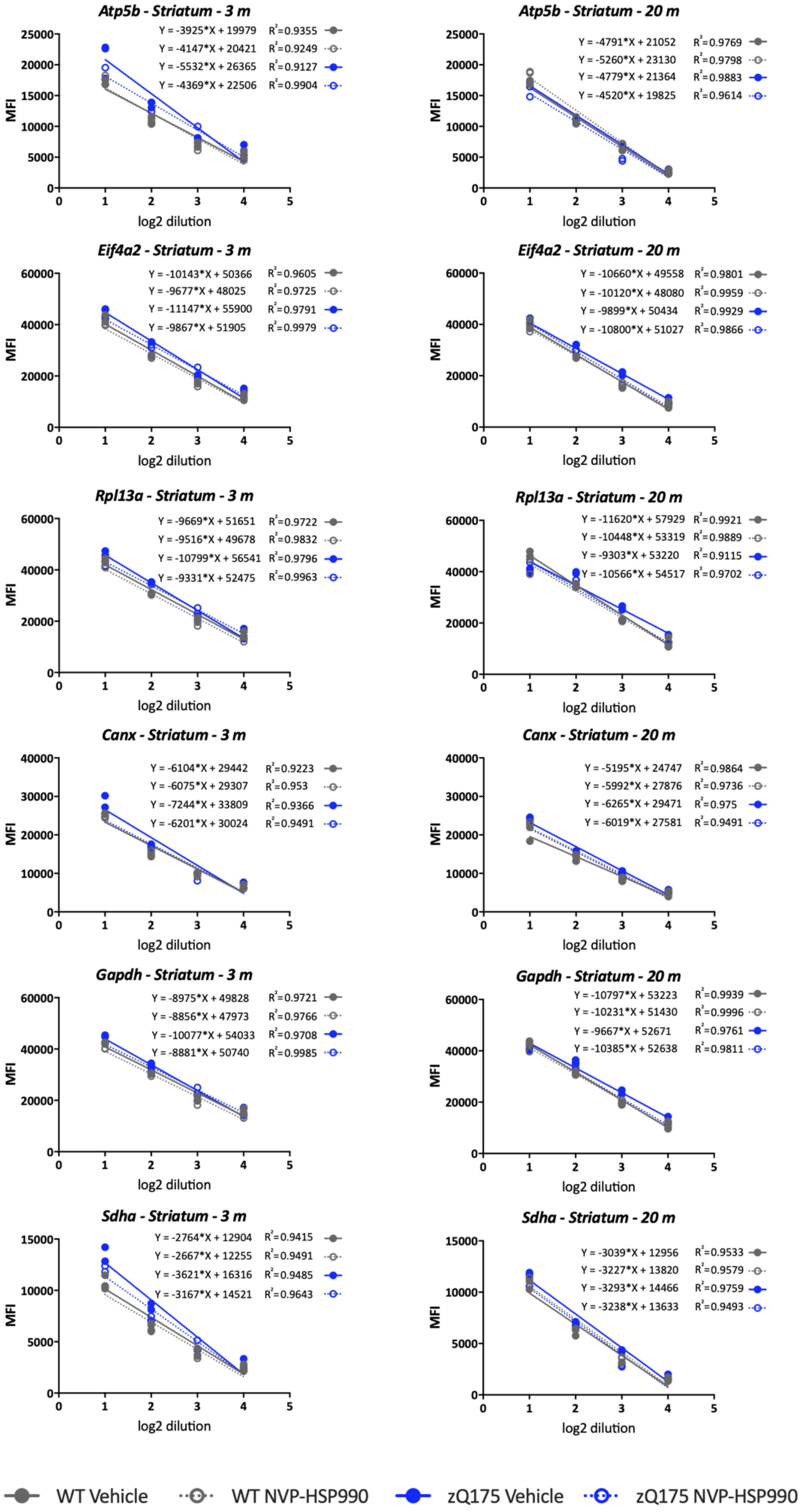

Supplementary Figure S10.

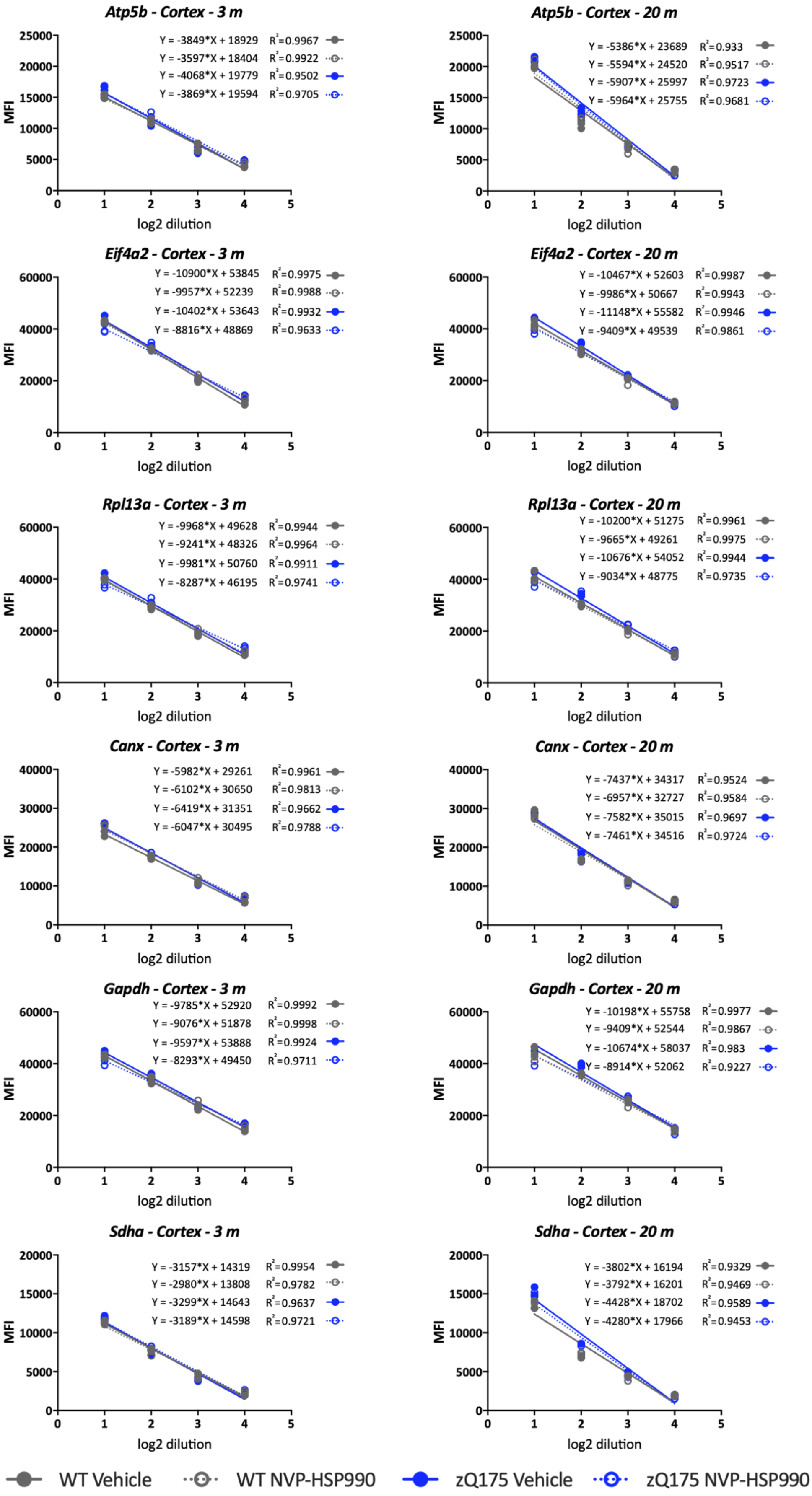

Supplementary Figure S11.

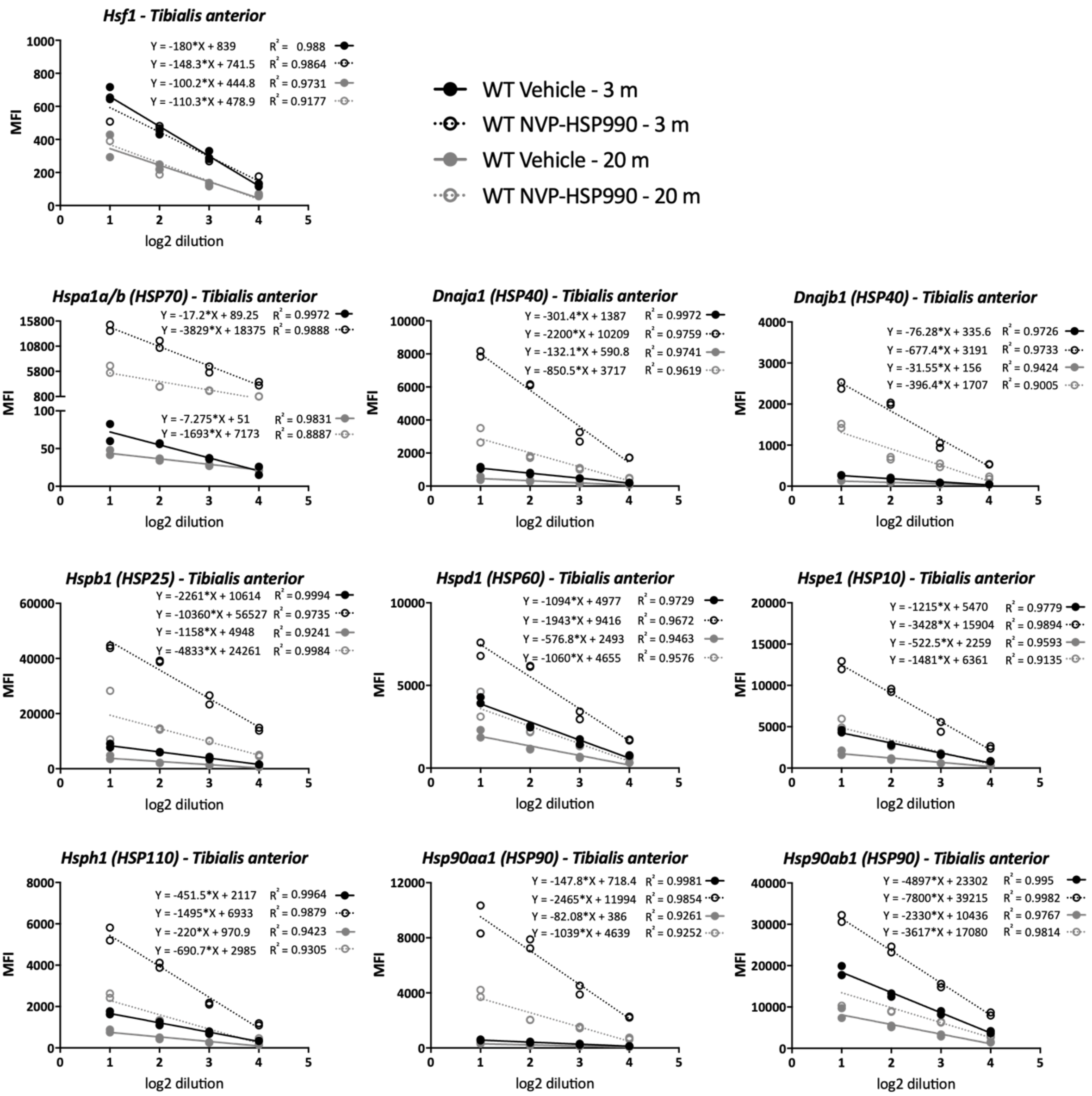

Supplementary Figure S11. Optimisation of QuantiGene 16-plex assay based on the MFI signals of the heat shock genes for use with zQ175 and wild-type tibialis anterior at 3 months and 20 months of age

Tibialis anterior samples were pooled (*n* = 6 / treatment / age) and subjected to a two-fold serial dilution and analysed in duplicate. The MFI signals for the corresponding muscle lysate dilutions from vehicle or NVP-HSP990 treated zQ175 and wild-type mice are shown. It was possible to observe the differences in signal between samples in which the heat shock response had, or had not, been induced. MFI = median fluorescent intensity, WT = wild-type; m = months.

Supplementary Figure S12.

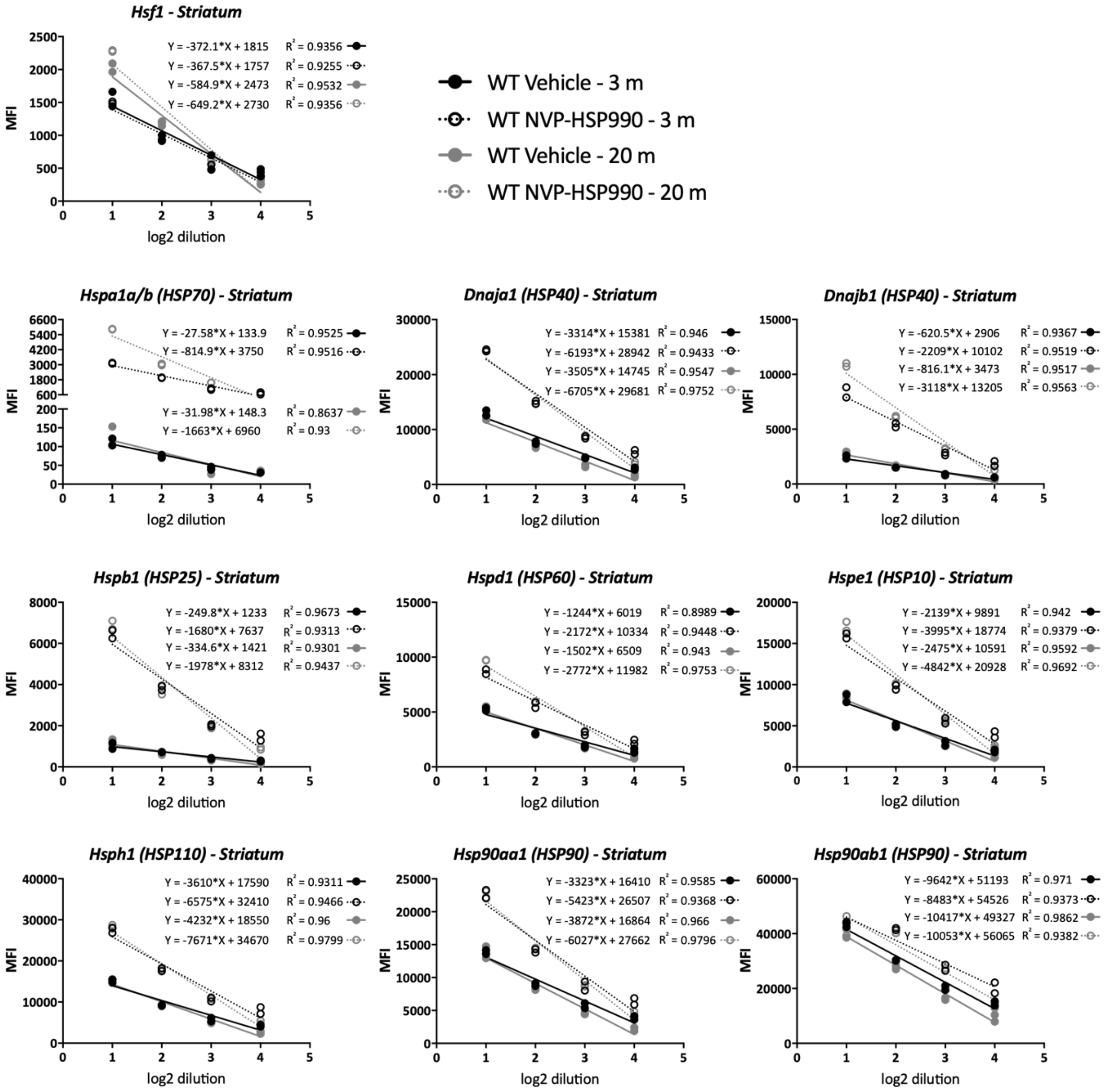

Supplementary Figure S12. Optimisation of QuantiGene 16-plex assay based on the MFI signals of the heat shock genes for use with zQ175 and wild-type striatum at 3 months and 20 months of age

Striatal samples were pooled (*n* = 6 / treatment / age) and subjected to a two-fold serial dilution and analysed in duplicate. The MFI signals for the corresponding striatal lysate dilutions from vehicle or NVP-HSP990 treated wild-type mice are shown. It was possible to observe the differences in signal between samples in which the heat shock response had, or had not, been induced. MFI = median fluorescent intensity, WT = wild-type; m = months.

Supplementary Figure S13.

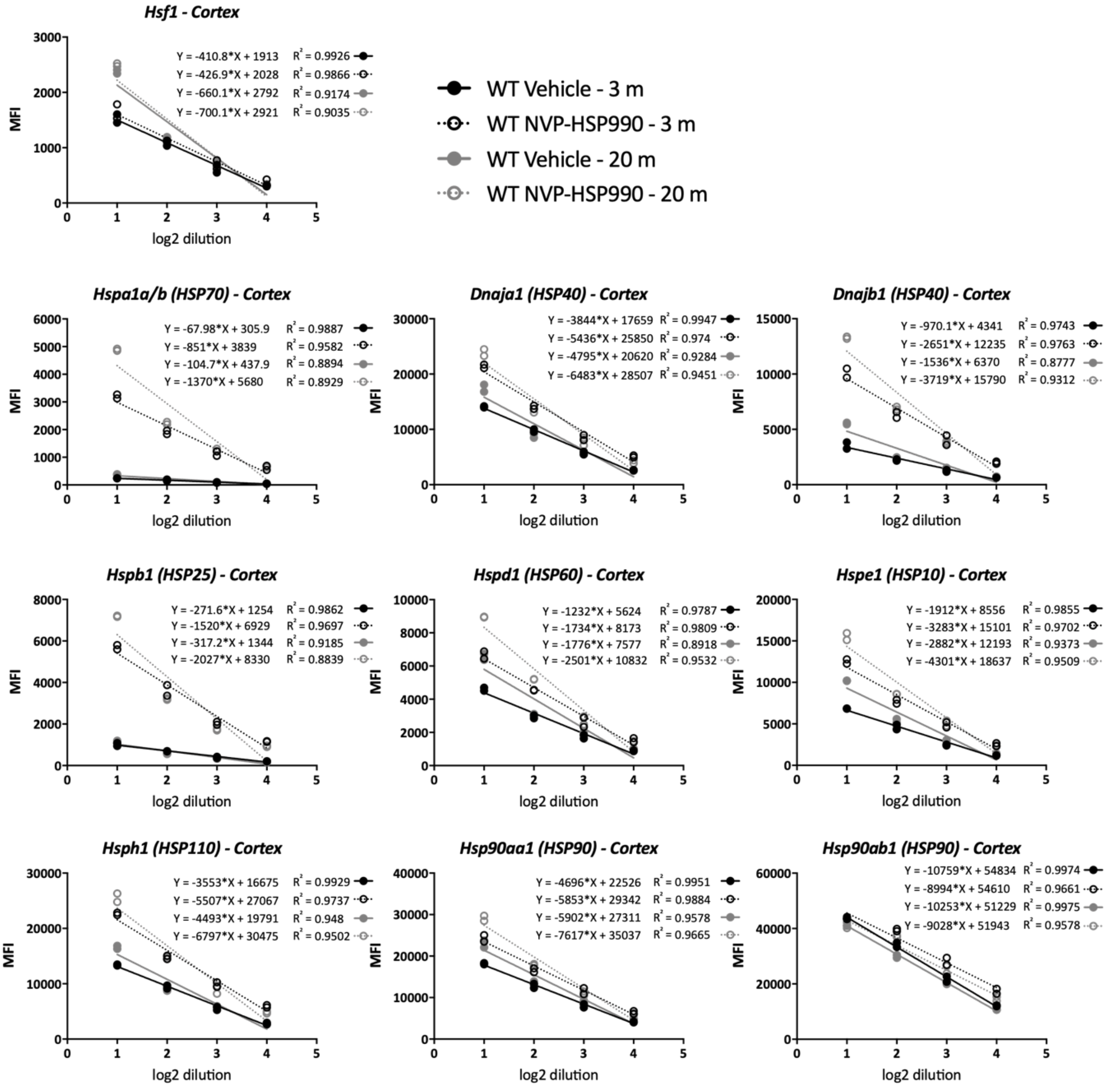

Supplementary Figure S13. Optimisation of QuantiGene 16-plex assay based on the MFI signals of the heat shock genes for use with zQ175 and wild-type cortex at 3 months and 20 months of age

Cortical samples were pooled (*n* = 6 / treatment / age) and subjected to a two-fold serial dilution and analysed in duplicate. The MFI signals for the corresponding cortical lysate dilutions from vehicle or NVP-HSP990 treated wild-type mice are shown. It was possible to observe the differences in signal between samples in which the heat shock response had, or had not, been induced. MFI = median fluorescent intensity, WT = wild-type; m = months.

Supplementary Figure S14. Analysis of the heat shock response in the cortex of zQ175 mice with disease progression

The expression of heat shock genes was measured using the QuantiGene 16-plex assay in the cortex of wild-type and zQ175 mice at 3, 12 and 20 months of age that had been treated with vehicle or NVP-HSP990. The NVP-HSP990 treated samples were normalised to the corresponding age-matched wild type vehicle treated samples. For simplicity, the wild-type vehicle samples and the zQ175 vehicle samples from the three ages were combined. Five NVP-HSP990 treated samples were excluded from the analysis, because the tibialis anterior heat shock gene expression levels were comparable to those treated with vehicle. These were: at 3 months, two wild type; at 12 months, one wild type; at 20 months, one wild type and one zQ175. N = 4-6 / genotype / treatment / age. Statistical analysis was by two-way ANOVA with Bonferroni correction for multiple comparisons. Mean ± SEM. *** $p \leq 0.001$. Test statistical values can be found in Supplementary Table S11. WT = wild-type; m = months.

Supplementary Figure S14.

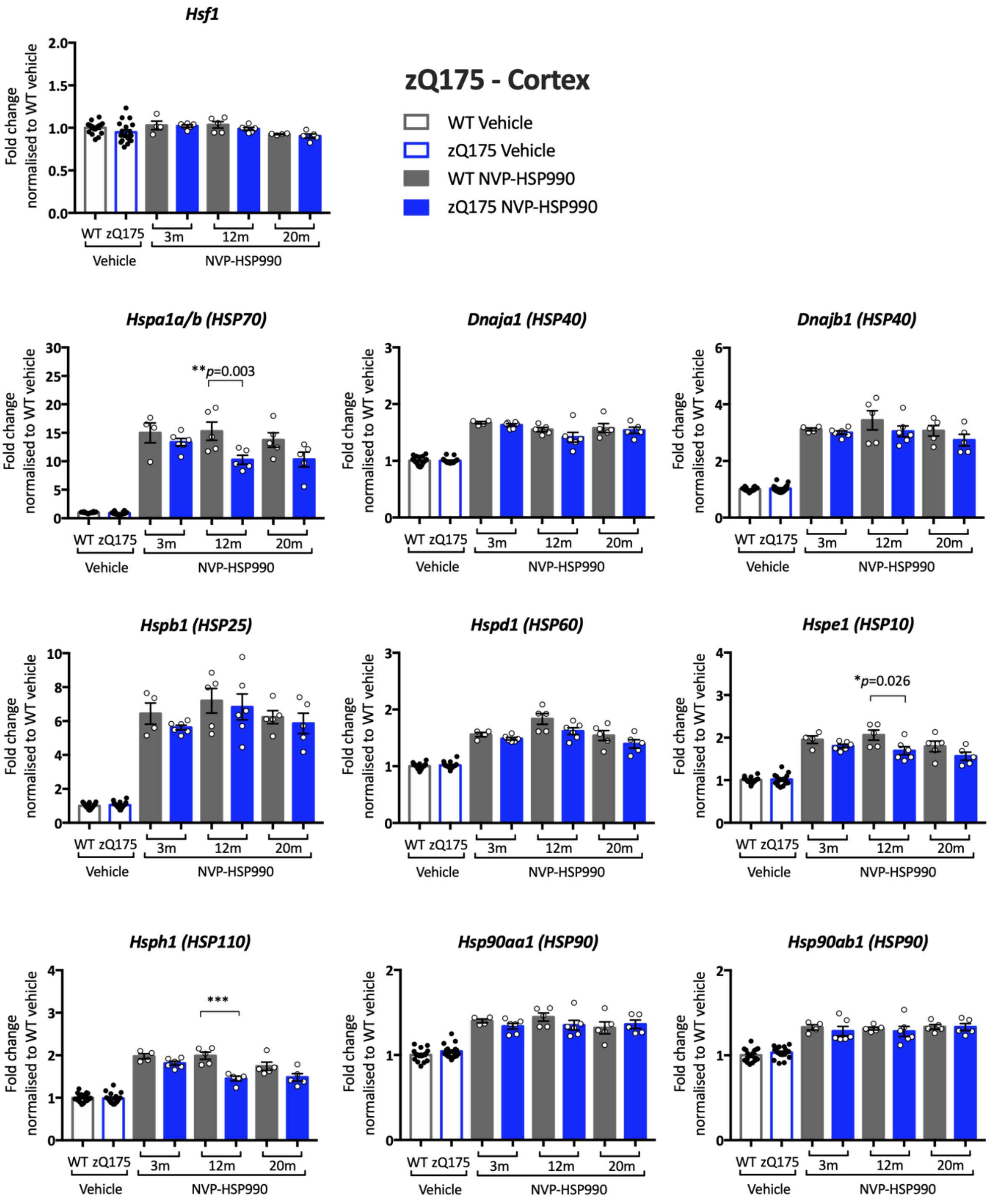

Supplementary Figure S15.

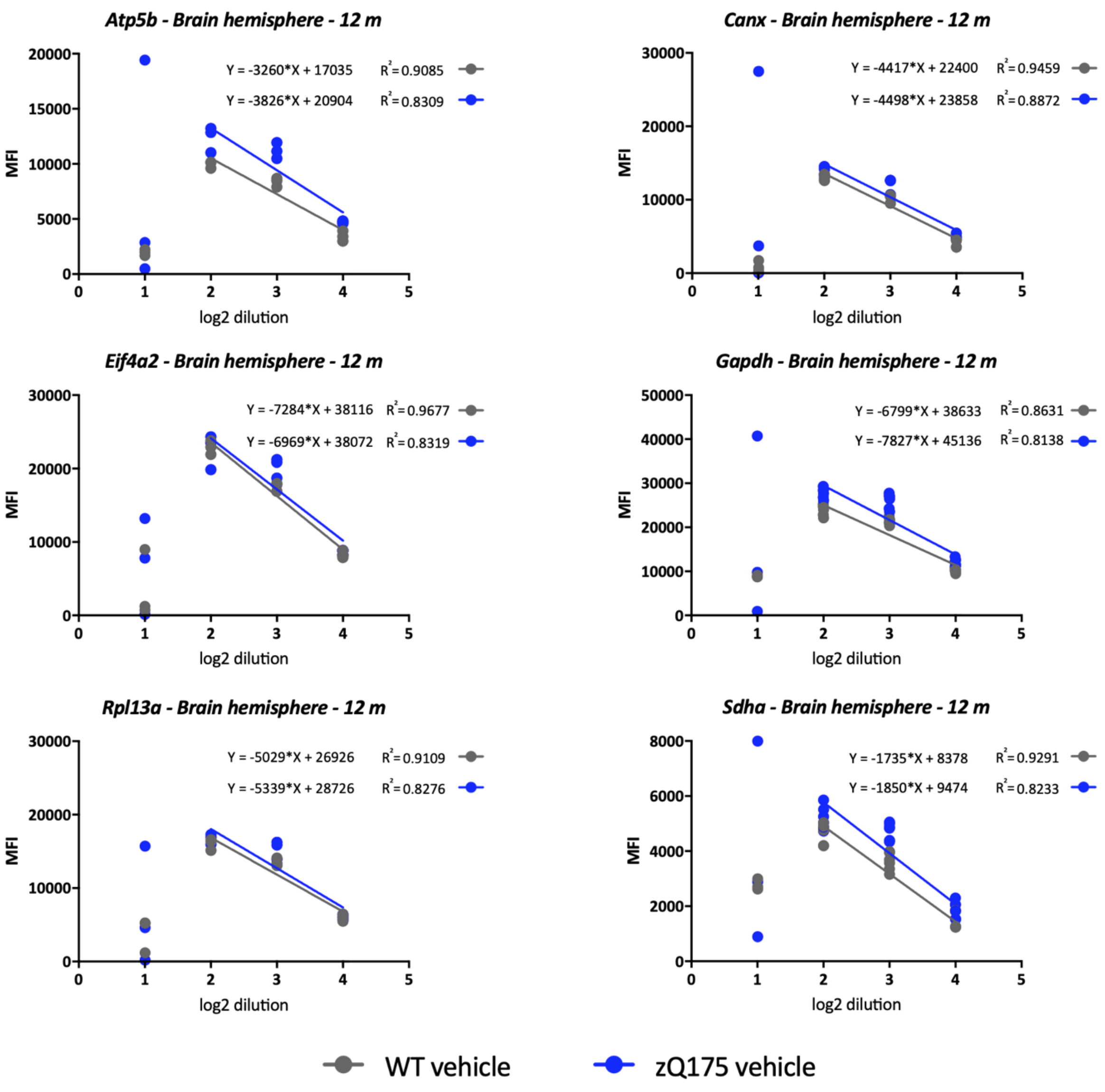

Supplementary Figure S15. Optimisation of the QuantiGene 16-plex assay based on the MFI signals of the housekeeping genes for use with zQ175 and wild-type brain hemispheres at 12 months of age

Brain samples were pooled (*n* = 8 / genotype) and subjected to a two-fold serial dilution and analysed in triplicate. The MFI signals for the corresponding brain lysate dilutions from vehicle or NVP-HSP990-treated zQ175 and wild-type mice are shown. MFI = median fluorescent intensity, WT = wild-type; m = months.

Supplementary Figure S16.

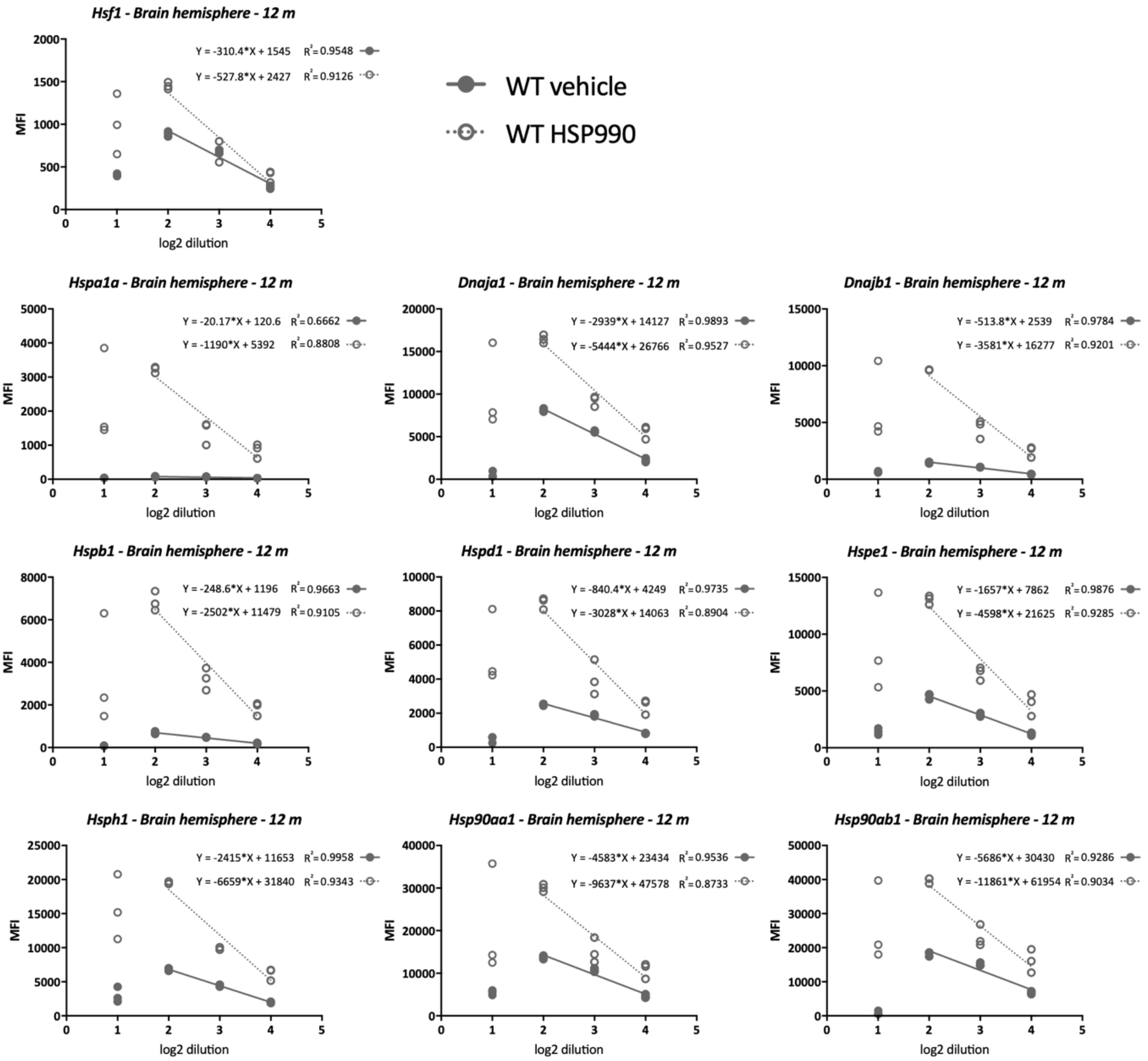

Supplementary Figure S16. Optimisation of QuantiGene 16-plex assay based on the MFI signals of the heat shock genes for use with zQ175 and wild-type brain hemisphere at 12 months of age

Brain hemisphere samples were pooled (*n* = 8 / treatment) and subjected to a two-fold serial dilution and analysed in triplicate. The MFI signals for the corresponding brain hemisphere lysate dilutions from vehicle or NVP-HSP990 treated wild-type mice are shown. It was possible to observe the differences in signal between samples in which the heat shock response had, or had not, been induced. MFI = median fluorescent intensity, WT = wild-type; m = months.

Supplementary Fig. S17

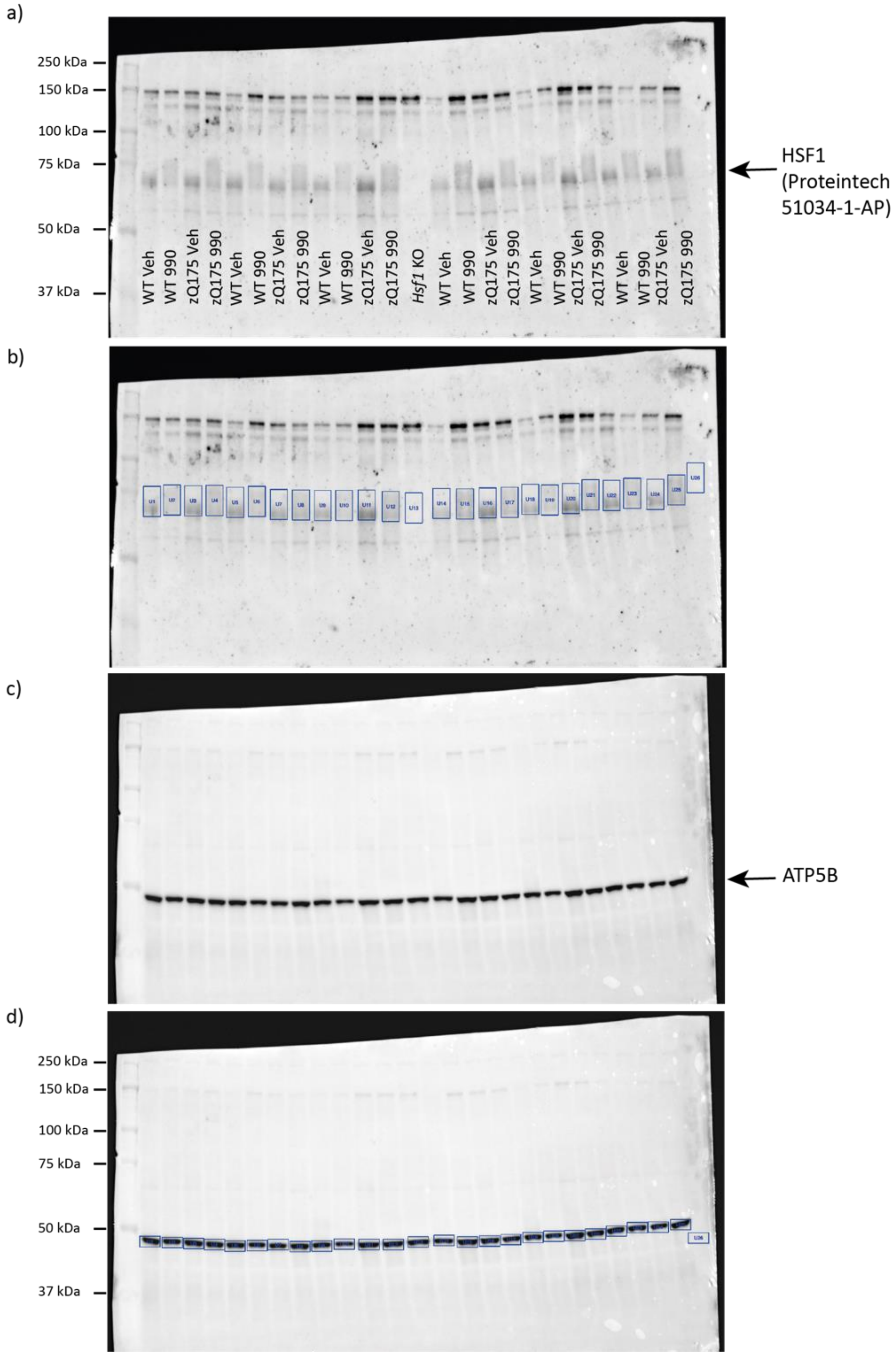

Supplementary Figure S17. Full-length western blots of HSF1 in brain hemispheres
Western blots (a) immunoprobed with HSF1 Proteintech 51034-1-AP antibody and (b) regions selected for quantification from the HSF1 antibody probed blot; (c) immunoprobed with ATP5B and (d) regions selected for quantification from the ATP5B probed blot. Veh = vehicle, 990 = NVP-HSP990; WT = wild-type.

Supplementary Figure S18.

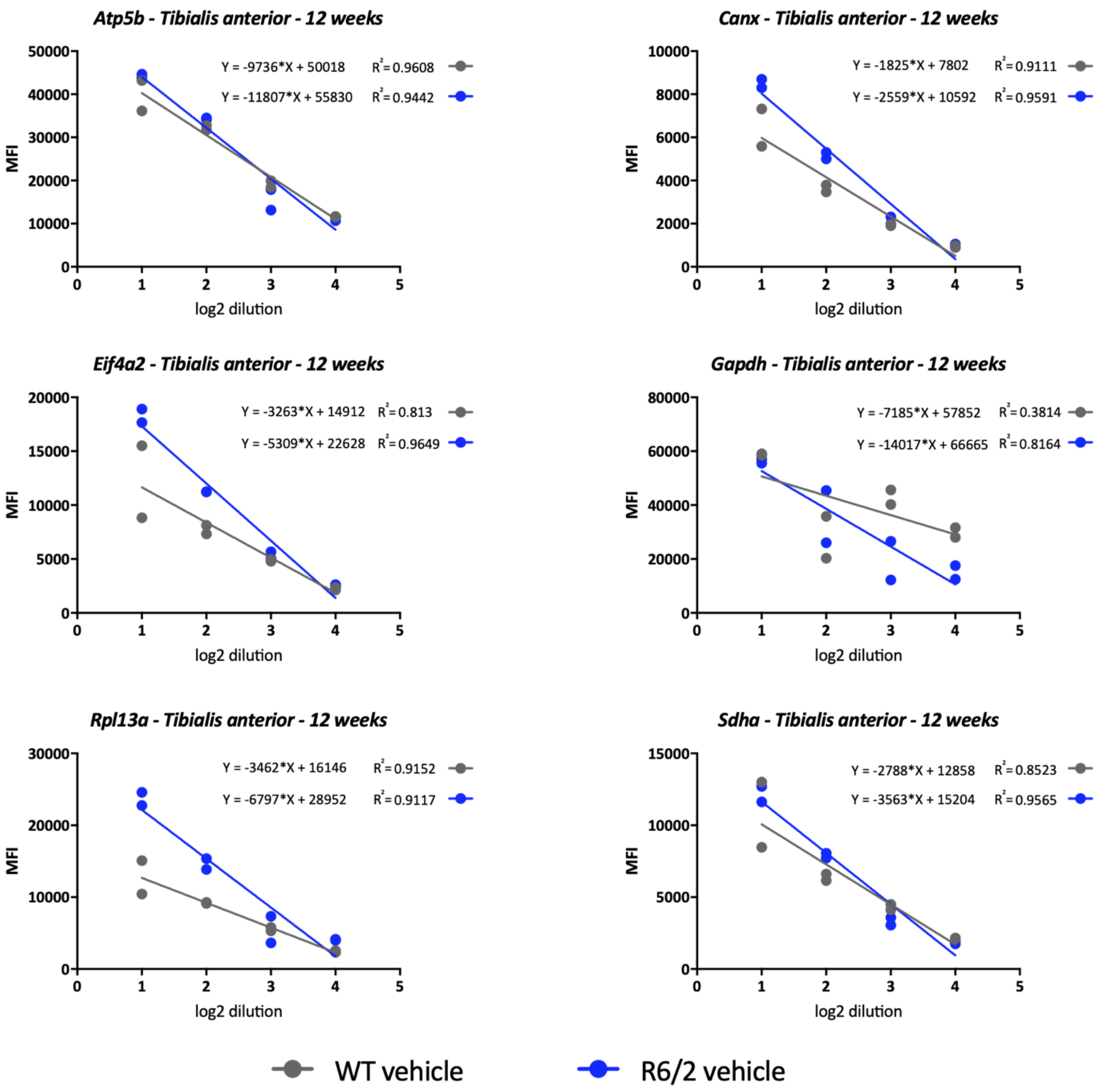

Supplementary Figure S18. Optimisation of the QuantiGene 16-plex assay based on the MFI signals of the housekeeping genes for use with R6/2 and wild-type tibialis anterior at 12 weeks of age

Tibialis anterior samples were pooled (*n* = 7-8 / genotype) and subjected to a two-fold serial dilution and analysed in duplicate. The MFI signals for the corresponding muscle lysate dilutions from vehicle or NVP-HSP990-treated R6/2 and wild-type mice are shown. MFI = median fluorescent intensity, WT = wild-type.

Supplementary Figure S19.

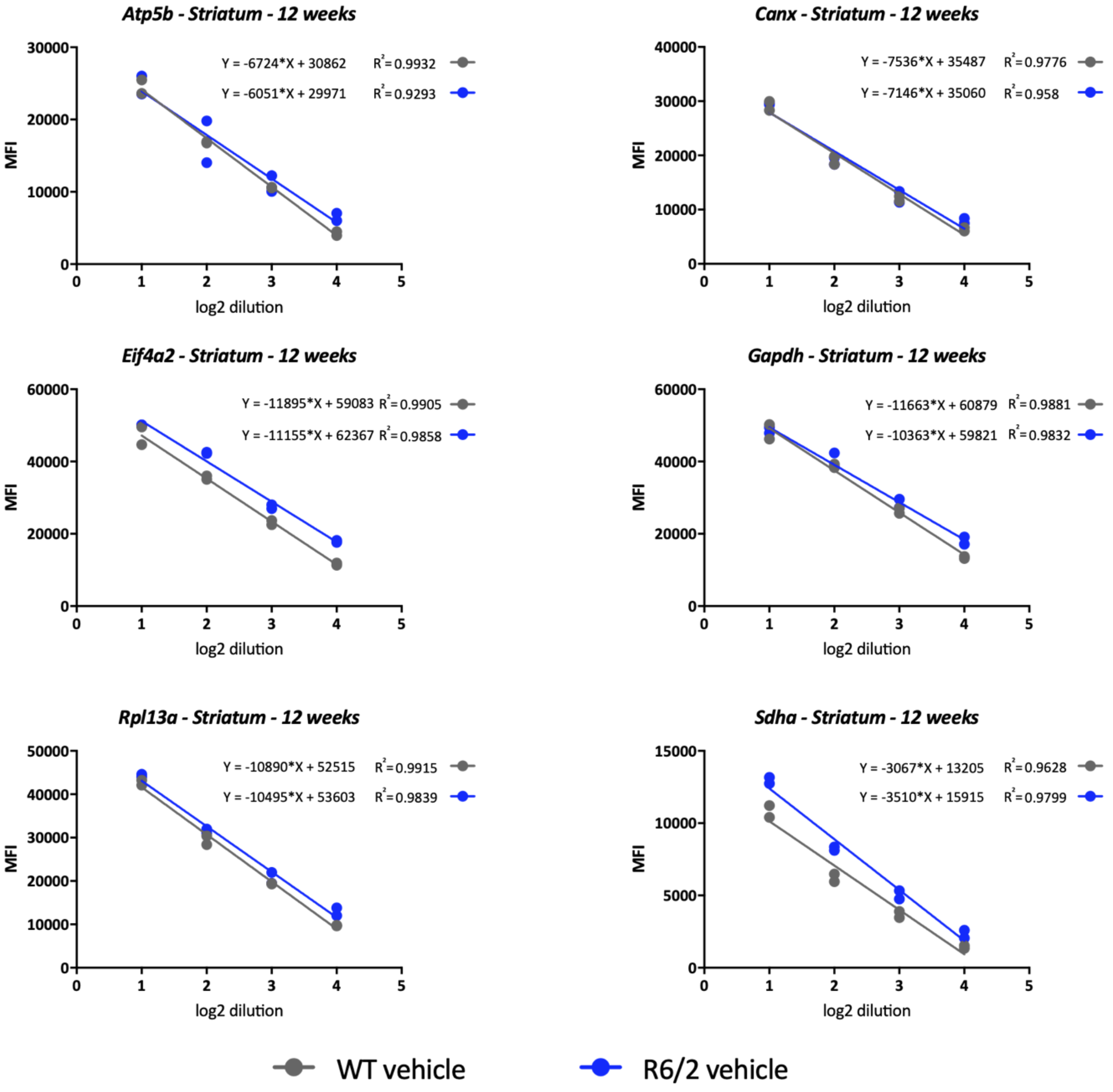

Supplementary Figure S19. Optimisation of the QuantiGene 16-plex assay based on the MFI signals of the housekeeping genes for use with R6/2 and wild-type striatum at 12 weeks of age

Striatal samples were pooled (*n* = 7-8 / genotype) and subjected to a two-fold serial dilution and analysed in duplicate. The MFI signals for the corresponding striatal lysate dilutions from vehicle or NVP-HSP990-treated R6/2 and wild-type mice are shown. MFI = median fluorescent intensity, WT = wild-type.

Supplementary Figure S20.

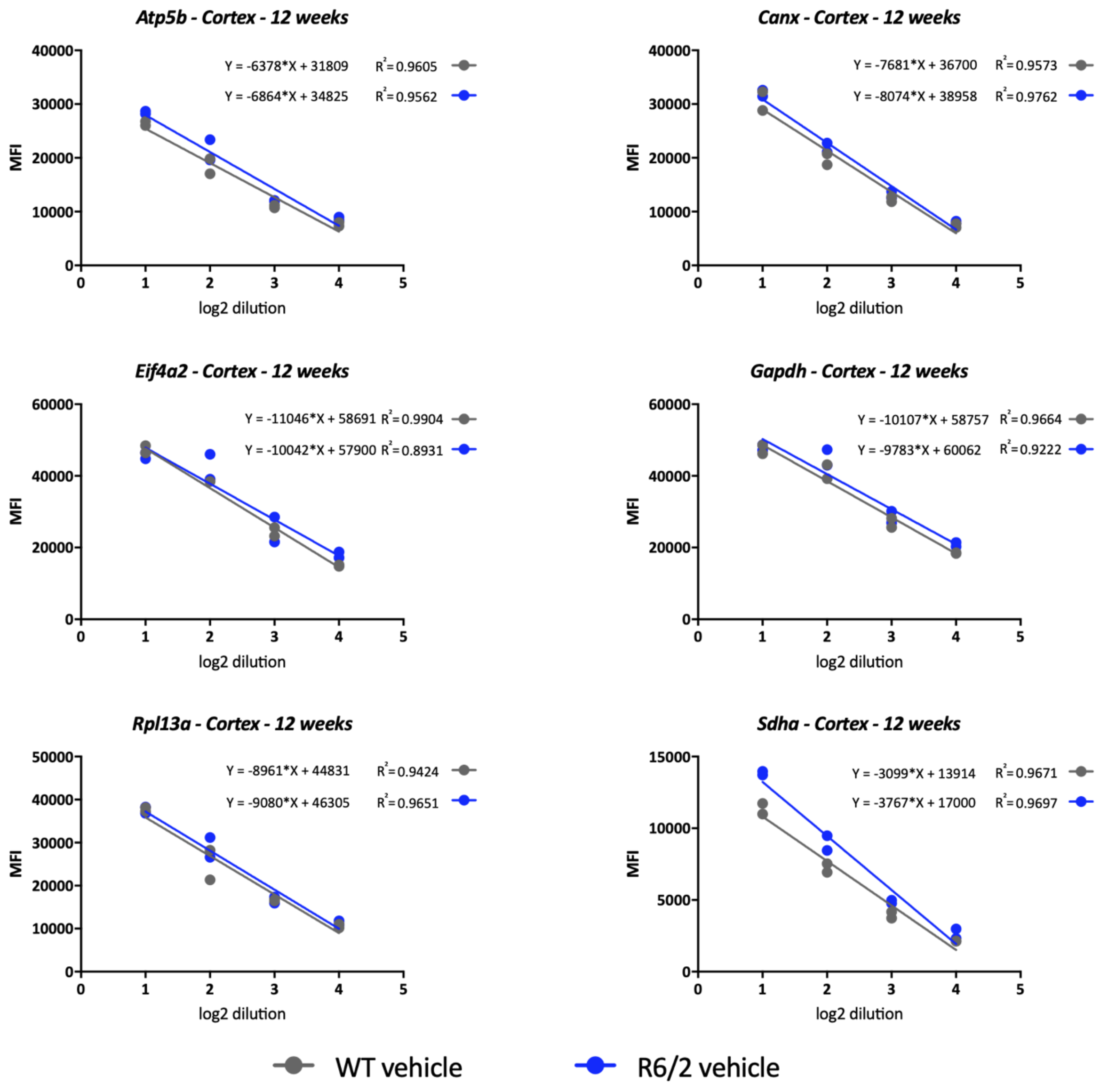

Supplementary Figure S20. Optimisation of the QuantiGene 16-plex assay based on the MFI signals of the housekeeping genes for use with R6/2 and wild-type cortex at 12 weeks of age

Cortical samples were pooled (*n* = 7-8 / genotype) and subjected to a two-fold serial dilution and analysed in duplicate. The MFI signals for the corresponding cortical lysate dilutions from vehicle or NVP-HSP990-treated zQ175 and wild-type mice are shown. MFI = median fluorescent intensity, WT = wild-type.

Supplementary Figure S21.

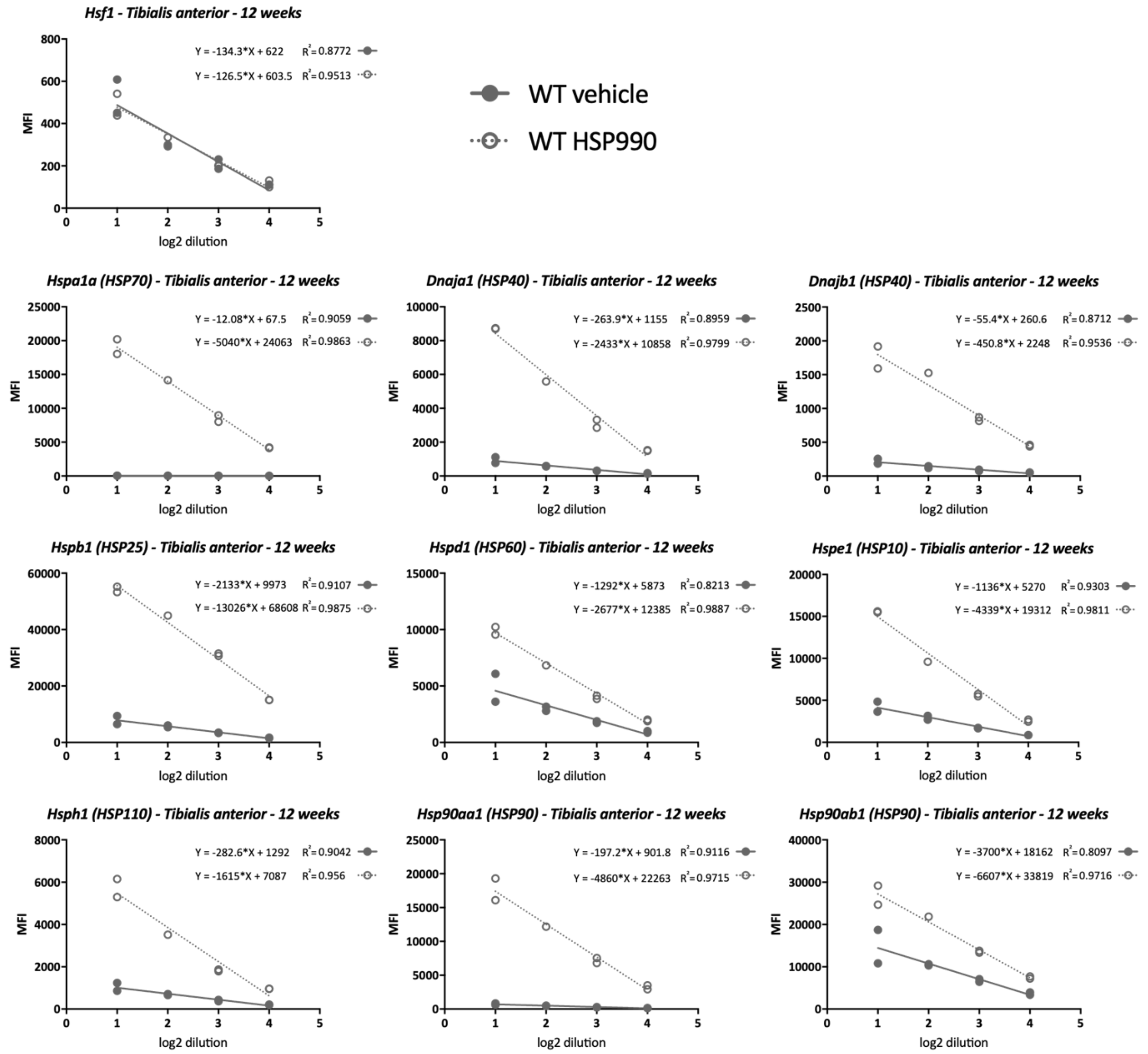

Supplementary Figure S21. Optimisation of QuantiGene 16-plex assay based on the MFI signals of the heat shock genes for use with R6/2 and wild-type cortex at 12 weeks of age Tibialis anterior samples were pooled (*n* = 7-9 / treatment) and subjected to a two-fold serial dilution and analysed in duplicate. The MFI signals for the corresponding muscle lysate dilutions from vehicle or NVP-HSP990 treated wild-type mice are shown. It was possible to observe the differences in signal between samples in which the heat shock response had, or had not, been induced. MFI = median fluorescent intensity, WT = wild-type.

Supplementary Figure S22.

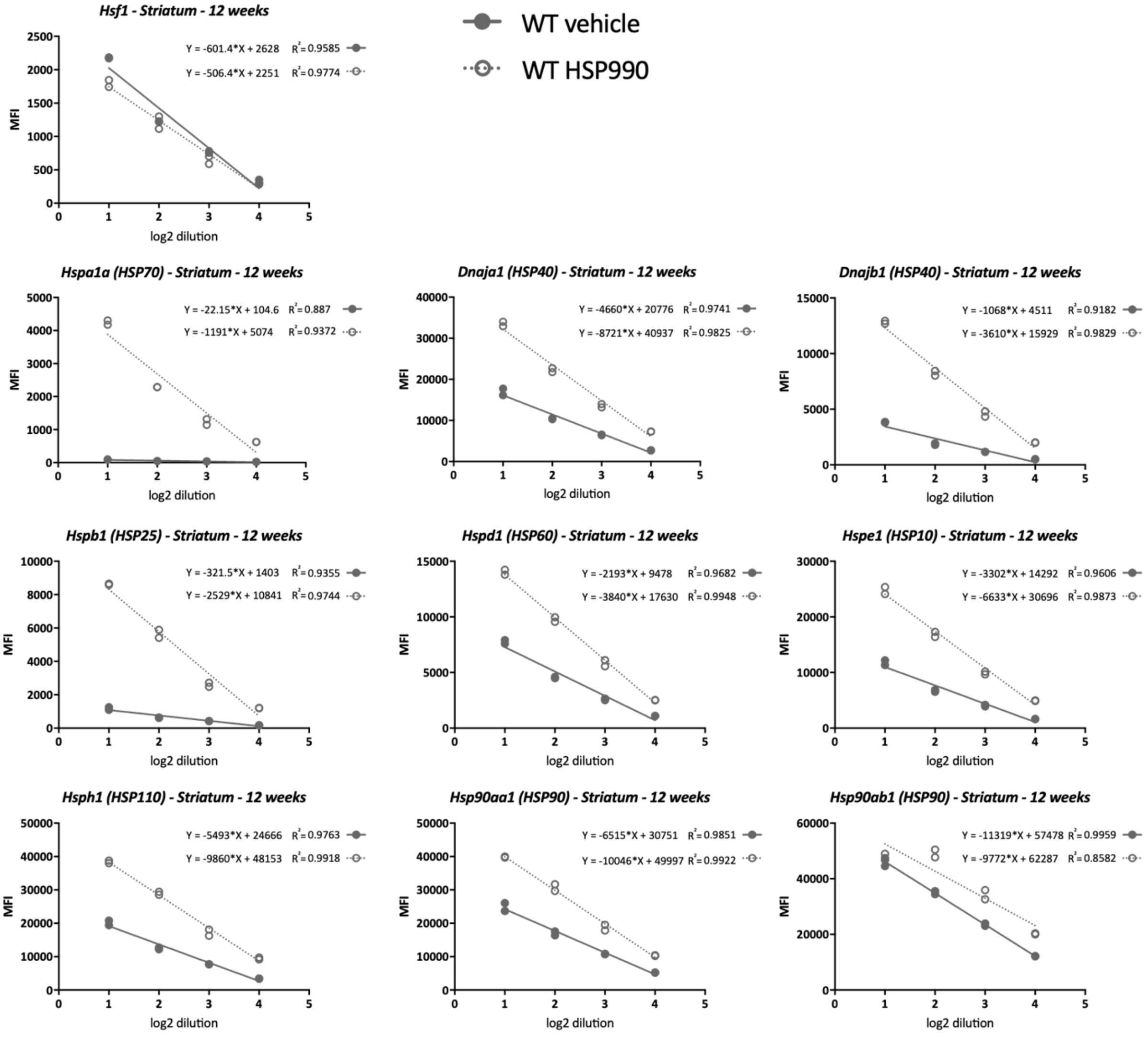

Supplementary Figure S22. Optimisation of QuantiGene 16-plex assay based on the MFI signals of the heat shock genes for use with R6/2 and wild-type striatum at 12 weeks of age Striatal samples were pooled (*n* = 7-9 / treatment) and subjected to a two-fold serial dilution and analysed in duplicate. The MFI signals for the corresponding cortical lysate dilutions from vehicle or NVP-HSP990 treated wild-type mice are shown. It was possible to observe the differences in signal between samples in which the heat shock response had, or had not, been induced. MFI = median fluorescent intensity, WT = wild-type.

Supplementary Figure S23.

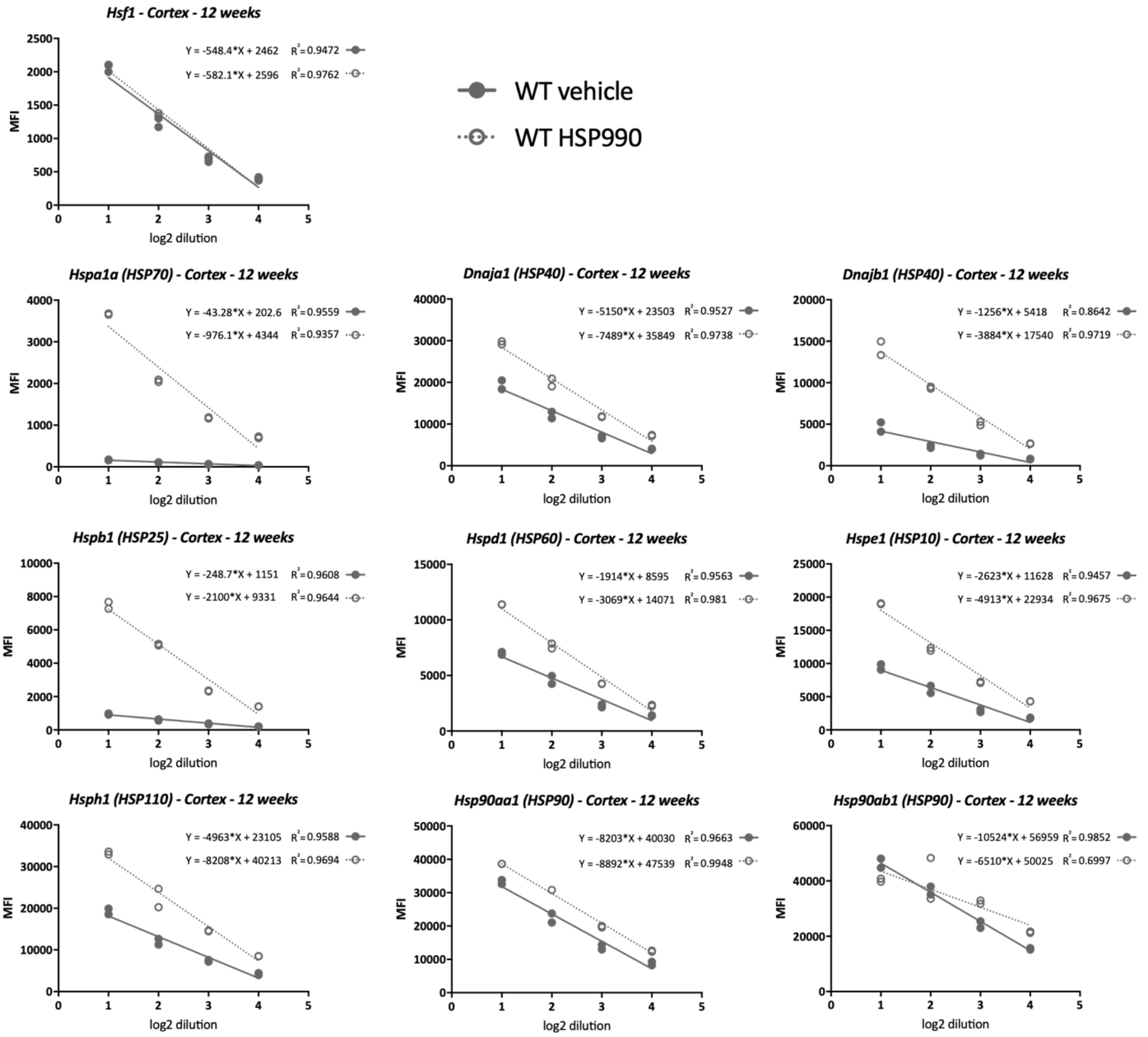

Supplementary Figure S23. Optimisation of QuantiGene 16-plex assay based on the MFI signals of the heat shock genes for use with R6/2 and wild-type cortex at 12 weeks of age
Cortical samples were pooled (*n* = 7-9 / treatment) and subjected to a two-fold serial dilution and analysed in duplicate. The MFI signals for the corresponding cortical lysate dilutions from vehicle or NVP-HSP990 treated wild type mice are shown. It was possible to observe the differences in signal between samples in which the heat shock response had, or had not, been induced. MFI = median fluorescent intensity, WT = wild-type.

Supplementary Figure S24. Analysis of the heat shock response in the cortex of R6/2 mice at 12 weeks of age

The expression of heat shock genes was measured using the QuantiGene 16-plex assay in the cortex of wild-type and R6/2 mice at 11-12 weeks of age that had been treated with vehicle or NVP-HSP990. The NVP-HSP990 treated samples were normalised to the corresponding wild-type vehicle treated samples. N = 6-9 / genotype / treatment. Statistical analysis was by two-way ANOVA with Bonferroni correction for multiple comparisons. Mean ± SEM. *** $p \leq 0.001$. Test statistical values can be found in Supplementary Table S16. WT = wild-type.

Supplementary Figure S24.

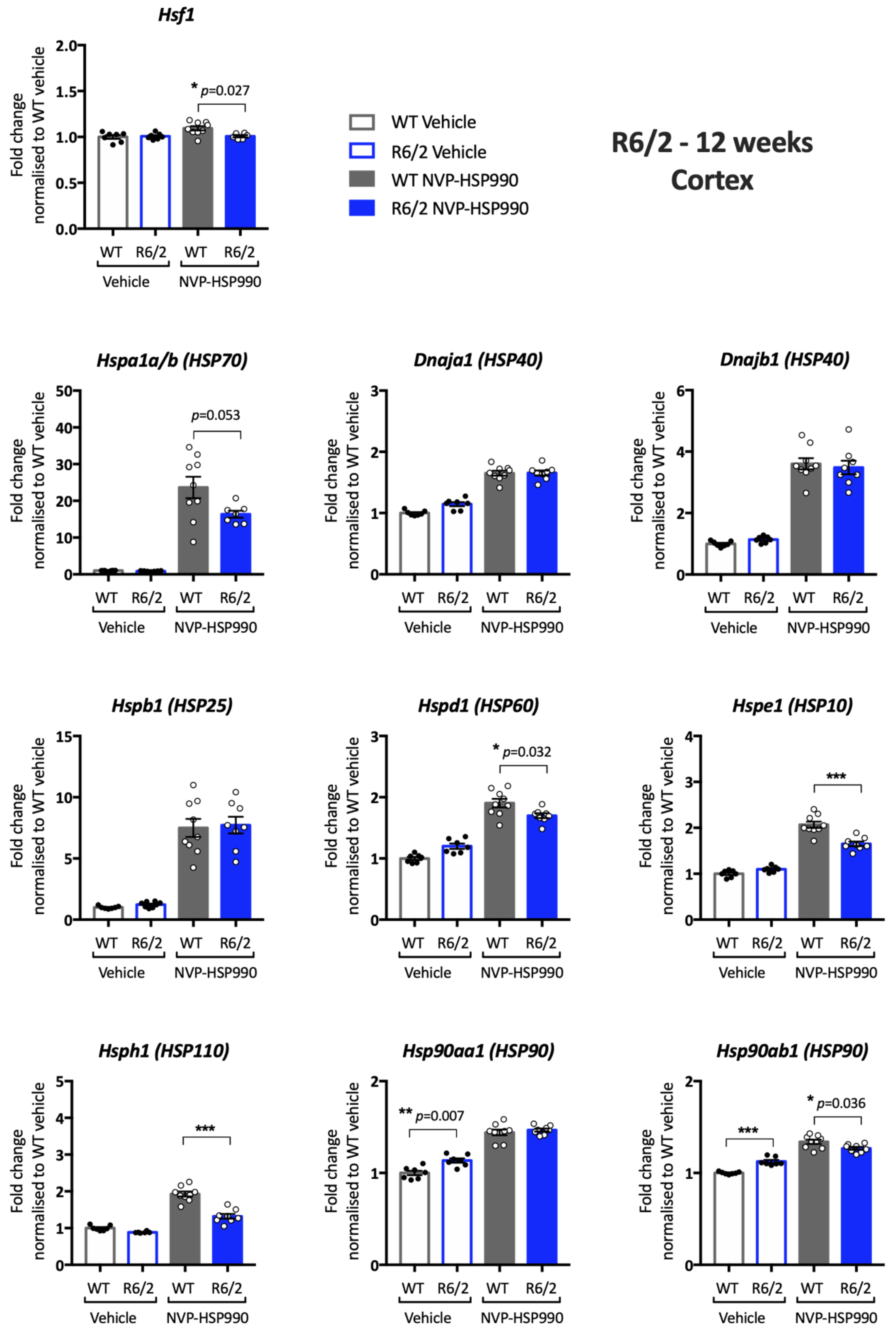

Supplementary Table S6. Statistical information for Fig. 1

Treatment group	Mouse tissue	Statistical values
Vehicle	Tibialis anterior	$t(9) = -1.671$, $p = 0.129$
	Striatum	$t(10) = -0.497$, $p = 0.630$
	Cortex	$t(10) = -0.094$, $p = 0.927$
NVP-HSP990	Tibialis anterior	$t(8) = 0.983$, $p = 0.354$
	Striatum	$t(8) = 0.194$, $p = 0.851$
	Cortex	$t(8) = 1.054$, $p = 0.323$

Supplementary Table S7. Statistical information for Fig. 2

Assay	Tissue	Gene	Statistical values
QuantiGene	Brain hemisphere	*Hsf1*	$t(9) = -1.367$, $p = 0.205$
		Hspa1a/b	$t(9) = -8.045$, $p < 0.001$
		Hspb1	$t(9) = -8.881$, $p < 0.001$
		Dnajb1	$t(9) = -13.939$, $p < 0.001$
	Tibialis anterior	*Hsf1*	$t(8) = 1.277$, $p = 0.237$
		Hspa1a/b	$t(8) = -10.056$, $p < 0.001$
		Hspb1	$t(9) = -11.840$, $p < 0.001$
		Dnajb1	$t(9) = -6.348$, $p < 0.001$
qPCR	Brain hemisphere	*Hsf1*	$t(8) = 0.430$, $p = 0.679$
		Hspa1a/b	$t(8) = -12.267$, $p < 0.001$
		Hspb1	$t(8) = -8.326$, $p < 0.001$
		Dnajb1	$t(8) = -11.905$, $p < 0.001$
	Tibialis anterior	*Hsf1*	$t(8) = -0.166$, $p = 0.872$
		Hspa1a/b	$t(9) = -13.998$, $p < 0.001$
		Hspb1	$t(8) = -15.341$, $p < 0.001$
		Dnajb1	$t(9) = -6.700$, $p < 0.001$

Supplementary Table S8. Statistical information for Supplementary Fig. S7

Gene	Time point	Statistic
Hsf1	4 hours	$t(6) = 0.083$, $p = 0.937$
	8 hours	$t(6) = 0.735$, $p = 0.490$
	12 hours	$t(6) = -0.437$, $p = 0.677$
	16 hours	$t(6) = 1.054$, $p = 0.332$
	20 hours	$t(6) = -0.725$, $p = 0.496$
Hspa1a/b	4 hours	$t(6) = -5.661$, $p = 0.001$
	8 hours	$t(6) = -17.741$, $p < 0.001$
	12 hours	$t(6) = -2.874$, $p = 0.028$
	16 hours	$t(5) = 1.096$, $p = 0.323$
	20 hours	$t(5) = -0.947$, $p = 0.387$
Hspb1	4 hours	$t(5) = -62.330$, $p < 0.001$
	8 hours	$t(6) = -13.408$, $p < 0.001$
	12 hours	$t(6) = -0.368$, $p = 0.725$
	16 hours	$t(6) = 1.184$, $p = 0.281$
	20 hours	$t(6) = -1.981$, $p = 0.095$
Dnajb1	4 hours	$t(6) = -12.755$, $p < 0.001$
	8 hours	$t(6) = -23.873$, $p < 0.001$
	12 hours	$t(5) = -1.878$, $p = 0.119$
	16 hours	$t(5) = -1.006$, $p = 0.361$
	20 hours	$t(6) = 0.491$, $p = 0.641$

Supplementary Table S9. Statistical information for Fig. 3

Gene	Age	Factor	Statistical values
Hsf1	3 months	Genotype	$F(1,17) = 0.011, p = 0.917$
		Treatment	$F(1,17) = 7.259, p = 0.015$
		Interaction	$F(1,17) = 0.113, p = 0.740$
	12 months	Genotype	$F(1,19) = 7.028, p = 0.016$
		Treatment	$F(1,19) = 0.152, p = 0.701$
		Interaction	$F(1,19) = 2.539, p = 0.128$
	20 months	Genotype	$F(1,18) = 0.248, p = 0.625$
		Treatment	$F(1,18) = 0.482, p = 0.496$
		Interaction	$F(1,18) = 1.360, p = 0.259$
Hspa1a/b	3 months	Genotype	$F(1,18) = 1.324, p = 0.265$
		Treatment	$F(1,18) = 6188.503, p < 0.001$
		Interaction	$F(1,18) = 2.427, p = 0.137$
	12 months	Genotype	$F(1,18) = 28.175, p < 0.001$
		Treatment	$F(1,18) = 1284.923, p < 0.001$
		Interaction	$F(1,18) = 29.612, p < 0.001$
	20 months	Genotype	$F(1,18) = 6.091, p = 0.024$
		Treatment	$F(1,18) = 130.390, p < 0.001$
		Interaction	$F(1,18) = 6.131, p = 0.023$
Dnaja1	3 months	Genotype	$F(1,17) = 4.989, p = 0.039$
		Treatment	$F(1,17) = 417.314, p < 0.001$
		Interaction	$F(1,17) = 7.930, p = 0.012$
	12 months	Genotype	$F(1,19) = 8.512, p = 0.009$
		Treatment	$F(1,19) = 334.655, p < 0.001$
		Interaction	$F(1,19) = 8.900, p = 0.008$
	20 months	Genotype	$F(1,18) = 0.786, p = 0.387$
		Treatment	$F(1,18) = 264.200, p < 0.001$
		Interaction	$F(1,18) = 1.725, p = 0.206$
Dnajb1	3 months	Genotype	$F(1,17) = 0.278, p = 0.605$
		Treatment	$F(1,17) = 563.463, p < 0.001$
		Interaction	$F(1,17) = 0.163, p = 0.692$
	12 months	Genotype	$F(1,19) = 13.568, p = 0.002$
		Treatment	$F(1,19) = 280.140, p < 0.001$
		Interaction	$F(1,19) = 14.155, p = 0.001$
	20 months	Genotype	$F(1,18) = 7.460, p = 0.014$
		Treatment	$F(1,18) = 132.255, p < 0.001$
		Interaction	$F(1,18) = 9.977, p = 0.005$
Hspb1	3 months	Genotype	$F(1,17) = 0.006, p = 0.941$
		Treatment	$F(1,17) = 1090.227, p < 0.001$
		Interaction	$F(1,17) = 0.210, p = 0.653$
	12 months	Genotype	$F(1,18) = 3.986, p = 0.061$
		Treatment	$F(1,18) = 307.950, p < 0.001$
		Interaction	$F(1,18) = 7.650, p = 0.013$
	20 months	Genotype	$F(1,18) = 4.742, p = 0.043$
		Treatment	$F(1,18) = 235.318, p < 0.001$
		Interaction	$F(1,18) = 5.283, p = 0.034$

Hspd1	3 months	Genotype	$F(1,18) = 0.615$, $p = 0.443$
		Treatment	$F(1,18) = 103.303$, $p < 0.001$
		Interaction	$F(1,18) = 1.314$, $p = 0.267$
	12 months	Genotype	$F(1,19) = 13.632$, $p = 0.002$
		Treatment	$F(1,19) = 211.436$, $p < 0.001$
		Interaction	$F(1,19) = 7.027$, $p = 0.016$
	20 months	Genotype	$F(1,18) = 23.362$, $p < 0.001$
		Treatment	$F(1,18) = 116.739$, $p < 0.001$
		Interaction	$F(1,18) = 15.051$, $p = 0.001$
Hspe1	3 months	Genotype	$F(1,18) = 1.364$, $p = 0.258$
		Treatment	$F(1,18) = 817.998$, $p < 0.001$
		Interaction	$F(1,18) = 5.270$, $p = 0.034$
	12 months	Genotype	$F(1,18) = 46.456$, $p < 0.001$
		Treatment	$F(1,18) = 505.187$, $p < 0.001$
		Interaction	$F(1,18) = 30.365$, $p < 0.001$
	20 months	Genotype	$F(1,18) = 16.120$, $p = 0.001$
		Treatment	$F(1,18) = 132.626$, $p < 0.001$
		Interaction	$F(1,18) = 14.178$, $p = 0.001$
Hsph1	3 months	Genotype	$F(1,18) = 0.064$, $p = 0.802$
		Treatment	$F(1,18) = 209.714$, $p < 0.001$
		Interaction	$F(1,18) = 0.735$, $p = 0.403$
	12 months	Genotype	$F(1,18) = 9.463$, $p = 0.007$
		Treatment	$F(1,18) = 235.832$, $p < 0.001$
		Interaction	$F(1,18) = 4.857$, $p = 0.041$
	20 months	Genotype	$F(1,18) = 9.678$, $p = 0.006$
		Treatment	$F(1,18) = 155.044$, $p < 0.001$
		Interaction	$F(1,18) = 2.365$, $p = 0.141$
Hsp90aa1	3 months	Genotype	$F(1,18) = 0.622$, $p = 0.441$
		Treatment	$F(1,18) = 739.225$, $p < 0.001$
		Interaction	$F(1,18) = 1.583$, $p = 0.224$
	12 months	Genotype	$F(1,19) = 6.952$, $p = 0.016$
		Treatment	$F(1,19) = 262.594$, $p < 0.001$
		Interaction	$F(1,19) = 7.698$, $p = 0.012$
	20 months	Genotype	$F(1,18) = 2.413$, $p = 0.138$
		Treatment	$F(1,18) = 116.920$, $p < 0.001$
		Interaction	$F(1,18) = 2.450$, $p = 0.135$
Hsp90ab1	3 months	Genotype	$F(1,18) = 0.005$, $p = 0.944$
		Treatment	$F(1,18) = 197.706$, $p < 0.001$
		Interaction	$F(1,18) = 2.259$, $p = 0.150$
	12 months	Genotype	$F(1,19) = 0.654$, $p = 0.429$
		Treatment	$F(1,19) = 166.941$, $p < 0.001$
		Interaction	$F(1,19) = 1.974$, $p = 0.176$
	20 months	Genotype	$F(1,18) = 0.130$, $p = 0.723$
		Treatment	$F(1,18) = 130.056$, $p < 0.001$
		Interaction	$F(1,18) = 0.610$, $p = 0.445$

Supplementary Table S10. Statistical information for Fig. 4

Gene	Age	Factor	Statistical values
Hsf1	3 months	Genotype	$F(1,17) = 0.013$, $p = 0.912$
		Treatment	$F(1,17) = 0.011$, $p = 0.918$
		Interaction	$F(1,17) = 5.114$, $p = 0.037$
	12 months	Genotype	$F(1,19) = 1.486$, $p = 0.238$
		Treatment	$F(1,19) = 1.348$, $p = 0.260$
		Interaction	$F(1,19) = 3.315$, $p = 0.084$
	20 months	Genotype	$F(1,16) = 0.988$, $p = 0.335$
		Treatment	$F(1,16) = 0.157$, $p = 0.697$
		Interaction	$F(1,16) = 0.245$, $p = 0.627$
Hspa1a/b	3 months	Genotype	$F(1,17) = 10.345$, $p = 0.005$
		Treatment	$F(1,17) = 792.711$, $p < 0.001$
		Interaction	$F(1,17) =11.926$, $p = 0.003$
	12 months	Genotype	$F(1,19) = 18.114$, $p < 0.001$
		Treatment	$F(1,19) = 295.583$, $p < 0.001$
		Interaction	$F(1,19) = 18.452$, $p < 0.001$
	20 months	Genotype	$F(1,18) = 0.881$, $p = 0.360$
		Treatment	$F(1,18) = 84.871$, $p < 0.001$
		Interaction	$F(1,18) = 1.107$, $p = 0.307$
Dnaja1	3 months	Genotype	$F(1,18) = 31.199$, $p < 0.001$
		Treatment	$F(1,18) = 812.910$, $p < 0.001$
		Interaction	$F(1,18) = 45.854$, $p < 0.001$
	12 months	Genotype	$F(1,19) = 9.404$, $p = 0.006$
		Treatment	$F(1,19) = 365.618$, $p < 0.001$
		Interaction	$F(1,19) = 16.200$, $p = 0.001$
	20 months	Genotype	$F(1,17) = 1.273$, $p = 0.275$
		Treatment	$F(1,17) = 114.185$, $p < 0.001$
		Interaction	$F(1,17) = 1.179$, $p = 0.293$
Dnajb1	3 months	Genotype	$F(1,18) = 1.791$, $p = 0.197$
		Treatment	$F(1,18) = 1250.723$, $p < 0.001$
		Interaction	$F(1,18) = 4.694$, $p = 0.044$
	12 months	Genotype	$F(1,19) = 2.525$, $p = 0.129$
		Treatment	$F(1,19) = 392.861$, $p < 0.001$
		Interaction	$F(1,19) = 5.170$, $p = 0.035$
	20 months	Genotype	$F(1,17) = 0.089$, $p = 0.769$
		Treatment	$F(1,17) = 384.243$, $p < 0.001$
		Interaction	$F(1,17) = 0.491$, $p = 0.493$
Hspb1	3 months	Genotype	$F(1,18) = 2.929$, $p = 0.104$
		Treatment	$F(1,18) = 326.304$, $p < 0.001$
		Interaction	$F(1,18) = 4.749$, $p = 0.043$
	12 months	Genotype	$F(1,18) = 6.835$, $p = 0.018$
		Treatment	$F(1,18) = 102.423$, $p < 0.001$
		Interaction	$F(1,18) = 6.126$, $p = 0.023$
	20 months	Genotype	$F(1,17) = 0.056$, $p = 0.815$
		Treatment	$F(1,17) = 83.720$, $p < 0.001$
		Interaction	$F(1,17) = 0.042$, $p = 0.840$

Hspd1	3 months	Genotype	*F*(1,18) = 22.980, *p* < 0.001
		Treatment	*F*(1,18) = 641.459, *p* < 0.001
		Interaction	*F*(1,18) = 30.531, *p* < 0.001
	12 months	Genotype	*F*(1,19) =8.776, *p* = 0.008
		Treatment	*F*(1,19) = 236.250, *p* < 0.001
		Interaction	*F*(1,19) = 22.115, *p* < 0.001
	20 months	Genotype	*F*(1,17) = 1.848, *p* = 0.192
		Treatment	*F*(1,17) = 126.059, *p* < 0.001
		Interaction	*F*(1,17) = 5.013, *p* = 0.039

Hspe1	3 months	Genotype	*F*(1,18) = 3.905, *p* = 0.064
		Treatment	*F*(1,18) = 260.323, *p* < 0.001
		Interaction	*F*(1,18) = 9.443, *p* = 0.007
	12 months	Genotype	*F*(1,19) = 44.670, *p* < 0.001
		Treatment	*F*(1,19) = 422.790, *p* < 0.001
		Interaction	*F*(1,19) = 39.547, *p* < 0.001
	20 months	Genotype	*F*(1,18) = 3.964, *p* = 0.062
		Treatment	*F*(1,18) = 79.258, *p* < 0.001
		Interaction	*F*(1,18) = 6.976, *p* = 0.017

Hsph1	3 months	Genotype	*F*(1,18) = 19.269, *p* < 0.001
		Treatment	*F*(1,18) = 378.433, *p* < 0.001
		Interaction	*F*(1,18) = 29.624, *p* < 0.001
	12 months	Genotype	*F*(1,19) = 26.121, *p* < 0.001
		Treatment	*F*(1,19) = 248.110, *p* < 0.001
		Interaction	*F*(1,19) = 17.953, *p* < 0.001
	20 months	Genotype	*F*(1,17) = 12.549, *p* = 0.003
		Treatment	*F*(1,17) = 90.902, *p* < 0.001
		Interaction	*F*(1,17) = 5.311, *p* = 0.034

Hsp90aa1	3 months	Genotype	*F*(1,18) = 1.541, *p* = 0.230
		Treatment	*F*(1,18) = 219.136, *p* < 0.001
		Interaction	*F*(1,18) = 12.921, *p* = 0.002
	12 months	Genotype	*F*(1,19) = 0.007, *p* = 0.933
		Treatment	*F*(1,19) = 238.029, *p* < 0.001
		Interaction	*F*(1,19) = 10.168, *p* = 0.005
	20 months	Genotype	*F*(1,18) = 2.343, *p* = 0.143
		Treatment	*F*(1,18) = 54.138, *p* < 0.001
		Interaction	*F*(1,18) = 5.448, *p* = 0.031

Hsp90ab1	3 months	Genotype	*F*(1,17) = 2.001, *p* = 0.175
		Treatment	*F*(1,17) = 84.216, *p* < 0.001
		Interaction	*F*(1,17) = 0.025, *p* = 0.876
	12 months	Genotype	*F*(1,19) = 1.466, *p* = 0.241
		Treatment	*F*(1,19) = 240.415, *p* < 0.001
		Interaction	*F*(1,19) = 13.131, *p* = 0.002
	20 months	Genotype	*F*(1,17) = 0.135, *p* = 0.718
		Treatment	*F*(1,17) = 27.662, *p* < 0.001
		Interaction	*F*(1,17) = 6.139, *p* = 0.024

Supplementary Table S11. Statistical information for Fig. S14

Gene	Age	Factor	Statistical values
Hsf1	3 months	Genotype	$F(1,18) = 1.377, p = 0.256$
		Treatment	$F(1,18) = 0.274, p = 0.607$
		Interaction	$F(1,18) = 1.967, p = 0.178$
	12 months	Genotype	$F(1,19) = 6.704, p = 0.018$
		Treatment	$F(1,19) = 4.548, p = 0.046$
		Interaction	$F(1,19) = 0.510, p = 0.484$
	20 months	Genotype	$F(1,17) = 11.584, p = 0.003$
		Treatment	$F(1,17) = 0.043, p = 0.838$
		Interaction	$F(1,17) = 6.731, p = 0.019$
Hspa1a/b	3 months	Genotype	$F(1,18) = 1.131, p = 0.302$
		Treatment	$F(1,18) = 336.750, p < 0.001$
		Interaction	$F(1,18) = 1.428, p = 0.248$
	12 months	Genotype	$F(1,18) = 10.202, p = 0.005$
		Treatment	$F(1,18) = 214.260, p < 0.001$
		Interaction	$F(1,18) = 9.054, p = 0.008$
	20 months	Genotype	$F(1,18) = 5.126, p = 0.036$
		Treatment	$F(1,18) = 184.634, p < 0.001$
		Interaction	$F(1,18) = 3.563, p = 0.075$
Dnaja1	3 months	Genotype	$F(1,16) = 3.127, p = 0.096$
		Treatment	$F(1,16) = 1015.339, p < 0.001$
		Interaction	$F(1,16) = 0.022, p = 0.883$
	12 months	Genotype	$F(1,18) = 1.464, p = 0.242$
		Treatment	$F(1,18) = 77.915, p < 0.001$
		Interaction	$F(1,18) = 1.637, p = 0.217$
	20 months	Genotype	$F(1,18) = 0.020, p = 0.889$
		Treatment	$F(1,18) = 116.744, p < 0.001$
		Interaction	$F(1,18) = 0.382, p = 0.544$
Dnajb1	3 months	Genotype	$F(1,18) = 0.226, p = 0.640$
		Treatment	$F(1,18) = 1345.032, p < 0.001$
		Interaction	$F(1,18) = 2.575, p = 0.126$
	12 months	Genotype	$F(1,19) = 1.203, p = 0.286$
		Treatment	$F(1,19) = 157.290, p < 0.001$
		Interaction	$F(1,19) = 1.159, p = 0.295$
	20 months	Genotype	$F(1,18) = 1.409, p = 0.251$
		Treatment	$F(1,18) = 208.133, p < 0.001$
		Interaction	$F(1,18) = 1.811, p = 0.195$
Hspb1	3 months	Genotype	$F(1,18) = 2.429, p = 0.137$
		Treatment	$F(1,18) = 419.764, p < 0.001$
		Interaction	$F(1,18) = 3.208, p = 0.090$
	12 months	Genotype	$F(1,19) = 0.137, p = 0.716$
		Treatment	$F(1,19) = 139.292, p < 0.001$
		Interaction	$F(1,19) = 0.122, p = 0.731$
	20 months	Genotype	$F(1,18) = 0.190, p = 0.668$
		Treatment	$F(1,18) = 237.214, p < 0.001$
		Interaction	$F(1,18) = 0.470, p = 0.502$

Hspd1	3 months	Genotype	$F(1,18) = 0.991$, $p = 0.333$
		Treatment	$F(1,18) = 269.323$, $p < 0.001$
		Interaction	$F(1,18) = 1.730$, $p = 0.205$
	12 months	Genotype	$F(1,19) = 3.295$, $p = 0.085$
		Treatment	$F(1,19) = 189.211$, $p < 0.001$
		Interaction	$F(1,19) = 5.354$, $p = 0.032$
	20 months	Genotype	$F(1,18) = 1.508$, $p = 0.235$
		Treatment	$F(1,18) = 71.289$, $p < 0.001$
		Interaction	$F(1,18) = 2.174$, $p = 0.158$

Hspe1	3 months	Genotype	$F(1,18) = 0.364$, $p = 0.554$
		Treatment	$F(1,18) = 235.230$, $p < 0.001$
		Interaction	$F(1,18) = 4.652$, $p = 0.045$
	12 months	Genotype	$F(1,17) = 5.946$, $p = 0.026$
		Treatment	$F(1,17) = 114.926$, $p < 0.001$
		Interaction	$F(1,17) = 4.232$, $p = 0.055$
	20 months	Genotype	$F(1,18) = 2.695$, $p = 0.118$
		Treatment	$F(1,18) = 81.100$, $p < 0.001$
		Interaction	$F(1,18) = 1.879$, $p = 0.187$

Hsph1	3 months	Genotype	$F(1,18) = 0.616$, $p = 0.443$
		Treatment	$F(1,18) = 244.607$, $p < 0.001$
		Interaction	$F(1,18) = 4.913$, $p = 0.040$
	12 months	Genotype	$F(1,18) = 28.066$, $p < 0.001$
		Treatment	$F(1,18) = 187.601$, $p < 0.001$
		Interaction	$F(1,18) = 21.848$, $p < 0.001$
	20 months	Genotype	$F(1,18) = 7.065$, $p = 0.016$
		Treatment	$F(1,18) = 99.961$, $p < 0.001$
		Interaction	$F(1,18) = 1.812$, $p = 0.195$

Hsp90aa1	3 months	Genotype	$F(1,18) = 0.009$, $p = 0.926$
		Treatment	$F(1,18) = 80.684$, $p < 0.001$
		Interaction	$F(1,18) = 3.197$, $p = 0.091$
	12 months	Genotype	$F(1,19) = 0.256$, $p = 0.619$
		Treatment	$F(1,19) = 69.578$, $p < 0.001$
		Interaction	$F(1,19) = 2.662$, $p = 0.119$
	20 months	Genotype	$F(1,17) = 0.322$, $p = 0.578$
		Treatment	$F(1,17) = 66.389$, $p < 0.001$
		Interaction	$F(1,17) = 0.194$, $p = 0.665$

Hsp90ab1	3 months	Genotype	$F(1,18) = 0.966$, $p = 0.339$
		Treatment	$F(1,18) = 60.376$, $p < 0.001$
		Interaction	$F(1,18) = 0.006$, $p = 0.938$
	12 months	Genotype	$F(1,19) = 0.359$, $p = 0.556$
		Treatment	$F(1,19) = 52.139$, $p < 0.001$
		Interaction	$F(1,19) = 2.428$, $p = 0.136$
	20 months	Genotype	$F(1,18) = 0.940$, $p = 0.345$
		Treatment	$F(1,18) = 125.056$, $p < 0.001$
		Interaction	$F(1,18) = 1.077$, $p = 0.313$

Supplementary Table S12. Statistical information for Fig. 5

Gene	Age	Factor	Statistical values
Hsf1	12 months	Genotype	$F(1,30) = 25.033$, $p < 0.001$
		Treatment	$F(1,30) = 17.697$, $p < 0.001$
		Interaction	$F(1,30) = 1.471$, $p = 0.235$
Hspa1a/b	12 months	Genotype	$F(1,29) = 43.716$, $p < 0.001$
		Treatment	$F(1,29) = 531.466$, $p < 0.001$
		Interaction	$F(1,29) = 40.976$, $p < 0.001$
Dnaja1	12 months	Genotype	$F(1,28) = 24.561$, $p < 0.001$
		Treatment	$F(1,28) = 72.571$, $p < 0.001$
		Interaction	$F(1,28) = 1.675$, $p = 0.206$
Dnajb1	12 months	Genotype	$F(1,30) = 13.675$, $p = 0.001$
		Treatment	$F(1,30) = 505.847$, $p < 0.001$
		Interaction	$F(1,30) = 15.707$, $p < 0.001$
Hspb1	12 months	Genotype	$F(1,29) = 7.569$, $p = 0.010$
		Treatment	$F(1,29) = 262.787$, $p < 0.001$
		Interaction	$F(1,29) = 8.580$, $p = 0.007$
Hspd1	12 months	Genotype	$F(1,29) = 1.102$, $p = 0.302$
		Treatment	$F(1,29) = 185.967$, $p < 0.001$
		Interaction	$F(1,29) = 2.417$, $p = 0.131$
Hspe1	12 months	Genotype	$F(1,29) = 70.997$ $p < 0.001$
		Treatment	$F(1,29) = 526.960$, $p < 0.001$
		Interaction	$F(1,29) = 33.991$, $p < 0.001$
Hsph1	12 months	Genotype	$F(1,26) = 46.124$, $p < 0.001$
		Treatment	$F(1,26) = 420.368$, $p < 0.001$
		Interaction	$F(1,26) = 15.070$, $p = 0.001$
Hsp90aa1	12 months	Genotype	$F(1,30) = 24.948$, $p < 0.001$
		Treatment	$F(1,30) = 91.149$, $p < 0.001$
		Interaction	$F(1,30) = 13.740$, $p = 0.001$
Hsp90ab1	12 months	Genotype	$F(1,30) = 5.113$, $p = 0.031$
		Treatment	$F(1,30) = 644.472$, $p < 0.001$
		Interaction	$F(1,30) = 12.634$, $p = 0.001$

Supplementary Table S13. Statistical information for Fig. 6

Treatment group	Mouse tissue	Statistical values
Vehicle	Brain hemisphere	$t(10) = -3.421$, $p = 0.007$
NVP-HSP990	Brain hemisphere	$t(9) = -1.899$, $p = 0.090$

Supplementary Table S14. Statistical information for Fig. 7

Gene	Age	Factor	Statistical values
Hsf1	12 weeks	Genotype	$F(1,27) = 3.023$, $p = 0.093$
		Treatment	$F(1,27) = 0.875$, $p = 0.358$
		Interaction	$F(1,27) = 3.595$, $p = 0.069$
Hspa1a/b	12 weeks	Genotype	$F(1,27) = 40.237$, $p < 0.001$
		Treatment	$F(1,27) = 96.673$, $p < 0.001$
		Interaction	$F(1,27) = 40.620$, $p < 0.001$
Dnaja1	12 weeks	Genotype	$F(1,28) = 1.045$, $p = 0.315$
		Treatment	$F(1,28) = 119.803$, $p < 0.001$
		Interaction	$F(1,28) = 4.844$, $p = 0.036$
Dnajb1	12 weeks	Genotype	$F(1,28) = 1.618$, $p = 0.214$
		Treatment	$F(1,28) = 68.676$, $p < 0.001$
		Interaction	$F(1,28) = 4.034$, $p = 0.054$
Hspb1	12 weeks	Genotype	$F(1,27) = 38.328$, $p < 0.001$
		Treatment	$F(1,27) = 254.154$, $p < 0.001$
		Interaction	$F(1,27) = 40.653$, $p < 0.001$
Hspd1	12 weeks	Genotype	$F(1,28) = 17.904$, $p < 0.001$
		Treatment	$F(1,28) = 57.393$, $p < 0.001$
		Interaction	$F(1,28) = 15.517$, $p < 0.001$
Hspe1	12 weeks	Genotype	$F(1,26) = 47.986$, $p < 0.001$
		Treatment	$F(1,26) = 128.540$, $p < 0.001$
		Interaction	$F(1,26) = 43.680$, $p < 0.001$
Hsph1	12 weeks	Genotype	$F(1,27) = 2.229$, $p = 0.147$
		Treatment	$F(1,27) = 176.747$, $p < 0.001$
		Interaction	$F(1,27) = 18.554$, $p < 0.001$
Hsp90aa1	12 weeks	Genotype	$F(1,27) = 21.099$, $p < 0.001$
		Treatment	$F(1,27) = 300.208$, $p < 0.001$
		Interaction	$F(1,27) = 29.162$, $p < 0.001$
Hsp90ab1	12 weeks	Genotype	$F(1,26) = 0.843$, $p = 0.367$
		Treatment	$F(1,26) = 33.104$, $p < 0.001$
		Interaction	$F(1,26) = 9.201$, $p = 0.005$

Supplementary Table S15. Statistical information for Fig. 8

Gene	Age	Factor	Statistical values
Hsf1	12 weeks	Genotype	$F(1,28) = 3.829$, $p = 0.060$
		Treatment	$F(1,28) = 1.443$, $p = 0.240$
		Interaction	$F(1,28) = 0.644$, $p = 0.429$
Hspa1a/b	12 weeks	Genotype	$F(1,26) = 3.210$, $p = 0.085$
		Treatment	$F(1,26) = 64.512$, $p < 0.001$
		Interaction	$F(1,26) = 3.430$, $p = 0.075$
Dnaja1	12 weeks	Genotype	$F(1,25) = 19.481$, $p < 0.001$
		Treatment	$F(1,25) = 907.665$, $p < 0.001$
		Interaction	$F(1,25) = 66.948$, $p < 0.001$
Dnajb1	12 weeks	Genotype	$F(1,28) = 1.795$, $p = 0.191$
		Treatment	$F(1,28) = 490.711$, $p < 0.001$
		Interaction	$F(1,28) = 0.196$, $p = 0.662$
Hspb1	12 weeks	Genotype	$F(1,28) = 2.832$, $p = 0.103$
		Treatment	$F(1,28) = 128.038$, $p < 0.001$
		Interaction	$F(1,28) = 2.362$, $p = 0.136$
Hspd1	12 weeks	Genotype	$F(1,28) = 38.722$, $p < 0.001$
		Treatment	$F(1,28) = 381.932$, $p < 0.001$
		Interaction	$F(1,28) = 62.718$, $p < 0.001$
Hspe1	12 weeks	Genotype	$F(1,28) = 62.823$, $p < 0.001$
		Treatment	$F(1,28) = 293.532$, $p < 0.001$
		Interaction	$F(1,28) = 43.593$, $p < 0.001$
Hsph1	12 weeks	Genotype	$F(1,28) = 92.308$, $p < 0.001$
		Treatment	$F(1,28) = 225.196$, $p < 0.001$
		Interaction	$F(1,28) = 45.256$, $p < 0.001$
Hsp90aa1	12 weeks	Genotype	$F(1,28) = 0.801$, $p = 0.379$
		Treatment	$F(1,28) = 275.159$, $p < 0.001$
		Interaction	$F(1,28) = 6.611$, $p = 0.016$
Hsp90ab1	12 weeks	Genotype	$F(1,28) = 11.596$, $p = 0.002$
		Treatment	$F(1,28) = 296.043$, $p < 0.001$
		Interaction	$F(1,28) = 38.812$, $p < 0.001$

Supplementary Table S16. Statistical information for Fig. S24

Gene	Age	Factor	Statistical values
Hsf1	12 weeks	Genotype	$F(1,25) = 4.078$, $p = 0.054$
		Treatment	$F(1,25) = 5.837$, $p = 0.023$
		Interaction	$F(1,25) = 5.578$, $p = 0.026$
Hspa1a/b	12 weeks	Genotype	$F(1,25) = 3.760$, $p = 0.064$
		Treatment	$F(1,25) = 99.392$, $p < 0.001$
		Interaction	$F(1,25) = 3.508$, $p = 0.073$
Dnaja1	12 weeks	Genotype	$F(1,27) = 4.468$, $p = 0.044$
		Treatment	$F(1,27) = 262.882$, $p < 0.001$
		Interaction	$F(1,27) = 3.929$, $p = 0.058$
Dnajb1	12 weeks	Genotype	$F(1,27) = 0.003$, $p = 0.958$
		Treatment	$F(1,27) = 242.233$, $p < 0.001$
		Interaction	$F(1,27) = 0.666$, $p = 0.421$
Hspb1	12 weeks	Genotype	$F(1,28) = 0.184$, $p = 0.672$
		Treatment	$F(1,28) = 141.843$, $p < 0.001$
		Interaction	$F(1,28) = 0.000$, $p = 0.998$
Hspd1	12 weeks	Genotype	$F(1,27) = 0.003$, $p = 0.956$
		Treatment	$F(1,27) = 186.423$, $p < 0.001$
		Interaction	$F(1,27) = 15.658$, $p < 0.001$
Hspe1	12 weeks	Genotype	$F(1,27) = 9.496$, $p = 0.005$
		Treatment	$F(1,27) = 250.461$, $p < 0.001$
		Interaction	$F(1,27) = 25.337$, $p < 0.001$
Hsph1	12 weeks	Genotype	$F(1,26) = 41.914$, $p < 0.001$
		Treatment	$F(1,26) = 149.306$, $p < 0.001$
		Interaction	$F(1,26) = 19.535$, $p < 0.001$
Hsp90aa1	12 weeks	Genotype	$F(1,27) = 10.201$, $p = 0.004$
		Treatment	$F(1,27) = 230.901$, $p < 0.001$
		Interaction	$F(1,27) = 4.709$, $p = 0.039$
Hsp90ab1	12 weeks	Genotype	$F(1,27) = 2.420$, $p = 0.131$
		Treatment	$F(1,27) = 185.282$, $p < 0.001$
		Interaction	$F(1,27) = 31.017$, $p < 0.001$
